# Exploring health in the UK Biobank: associations with sociodemographic characteristics, psychosocial factors, lifestyle and environmental exposures

**DOI:** 10.1186/s12916-021-02097-z

**Published:** 2021-10-11

**Authors:** Julian Mutz, Charlotte J. Roscoe, Cathryn M. Lewis

**Affiliations:** 1 Social, Genetic and Developmental Psychiatry Centre, Institute of Psychiatry, Psychology & Neuroscience, King’s College London, Memory Lane, London, SE5 8AF UK; 2 MRC Centre for Environment and Health, School of Public Health, Imperial College London, Norfolk Place, London, W2 1PG UK; 3 Harvard T.H. Chan School of Public Health, Harvard University, Landmark Center, 401 Park Drive, Boston, MA 02215 USA; 4 Department of Medical & Molecular Genetics, Faculty of Life Sciences & Medicine, King’s College London, Great Maze Pond, London, SE1 9RT UK

**Keywords:** Health status, Self-rated health, Long-standing illness, Lifestyle, Environmental exposures, Sociodemographic characteristics, Psychosocial factors, UK Biobank, Epidemiology, Public health

## Abstract

**Background:**

A greater understanding of the factors that are associated with favourable health may help increase longevity and healthy life expectancy. We examined sociodemographic, psychosocial, lifestyle and environmental exposures associated with multiple health indicators.

**Methods:**

UK Biobank recruited > 500,000 participants, aged 37–73, between 2006 and 2010. Health indicators examined were 81 cancer and 443 non-cancer illnesses used to classify participants' health status; long-standing illness; and self-rated health. Exposures were sociodemographic (age, sex, ethnicity, education, income and deprivation), psychosocial (loneliness and social isolation), lifestyle (smoking, alcohol intake, sleep duration, BMI, physical activity and stair climbing) and environmental (air pollution, noise and residential greenspace) factors. Associations were estimated using logistic and ordinal logistic regression.

**Results:**

In total, 307,378 participants (mean age = 56.1 years [SD = 8.07], 51.9% female) were selected for cross-sectional analyses. Low income, being male, neighbourhood deprivation, loneliness, social isolation, short or long sleep duration, low or high BMI and smoking were associated with poor health. Walking, vigorous-intensity physical activity and more frequent alcohol intake were associated with good health. There was some evidence that airborne pollutants (PM_2.5_, PM_10_ and NO_2_) and noise (L_den_) were associated with poor health, though findings were not consistent across all models.

**Conclusions:**

Our findings highlight the multifactorial nature of health, the importance of non-medical factors, such as loneliness, healthy lifestyle behaviours and weight management, and the need to examine efforts to improve the health outcomes of individuals on low incomes.

**Supplementary Information:**

The online version contains supplementary material available at 10.1186/s12916-021-02097-z.

## Background

Substantial improvements in human health and significant increases in average life expectancy are major achievements of civilization over the past two centuries [1]. Life expectancy at birth in England and Wales has doubled from about 40 years in 1850 to more than 80 years in 2013 [2]. Future life expectancy is projected to increase across 35 industrialised nations, with a median increase in life expectancy at birth of approximately 3 years for women and 4 years for men in the UK between 2010 and 2030 [3].

Many adults, however, spend a substantial portion of their lives with late-life morbidities and decreased sensory, motor and cognitive functioning, which may reduce their quality of life [4, 5]. More than 80% of the population of England and Wales aged 85 and above report having a disability and 50% require care or help with daily activities [6]. Recent data suggest that healthy life expectancy in the UK has decreased between 2008 and 2016 [7]. Nevertheless, some individuals experience very little functional decline in old age and rate their health as good or excellent [8]. A greater understanding of the factors associated with good health may help increase longevity and healthspan, i.e. the length of time that a person lives healthy [9].

The health effects of lifestyle factors such as smoking [10], diet [11], excessive alcohol intake [12] and physical activity [13] are well documented. However, the number and type of covariates that have been adjusted for in previous analyses vary widely [14], making a systematic comparison of risk factor associations across studies difficult. Few studies have jointly examined sociodemographic, psychosocial, lifestyle and environmental factors. A review of multivariable models examining determinants of self-reported health concluded that most factors except for age, sex and education were examined in only one or a few studies [15]. In addition, research has often focused on predicting mortality or disease incidence instead of overall health status, despite its potential to provide insights into the factors associated with increased healthspan.

The UK Biobank study provides an unprecedented data resource to investigate determinants of health and ageing trajectories. One of its strengths is that the data collection was not merely focused on established predictors of health and disease, such as lifestyle, but also included relevant non-medical factors such as social isolation and loneliness, as well as road traffic noise and ambient air pollution [16, 17].

The aim of this study was to explore predictors of health status from a wide range of potential risk factor mechanisms. More specifically, we examined how health status was associated with (i) sociodemographic characteristics and psychosocial factors, (ii) lifestyle factors, and (iii) environmental exposures, both independently and jointly. We also examined, as a secondary aim, whether there was evidence of similar associations between these factors and (i) reporting a long-standing illness and (ii) self-rated health.

## Methods

### Study population

The UK Biobank is a prospective study of over 500,000 UK residents aged 37–73 at baseline, recruited between 2006 and 2010. Details of the study rationale and design have been reported elsewhere [18]. Briefly, individuals registered with the UK National Health Service (NHS) and living within a 25-mile (~ 40 km) radius of one of 22 assessment centres were invited to participate (9,238,453 postal invitations sent). At the baseline assessment, participants completed questionnaires and were interviewed by nurses to provide data on sociodemographic characteristics, health behaviours and their medical history. A small subset of participants completed repeat measurements: first revisit of participants living within a 35-km radius of the assessment centre at Stockport, England, between 2012 and 2013 (20,344 participants); second revisit as part of the UK Biobank Imaging Study [19] between 2014 and 2019 (43,190 participants at the time of this analysis). Participants consented to use of their de-identified data. See Additional file 1: Table S1 for the UK Biobank data fields used.

### Exposures

Multiple explanatory variables were considered based on their likely relevance to public health in terms of modifiability and potential for risk stratification, representing a wide range of potential risk factor mechanisms.

#### Sociodemographic characteristics

We considered individual-level sociodemographic characteristics including age at baseline assessment, sex, ethnicity (White, Asian, Black, Chinese, Mixed-race or other), highest educational or professional qualification (four categories, reflecting similar years of education [20]: (1) College/University Degree; (2) Education to age 18 or above, but not reaching degree level: General Certificate of Education Advanced Level (A levels) / General Certificate of Education Advanced Subsidiary Level (AS levels) or equivalent, National Vocational Qualification (NVQ) / Higher National Diploma (HND) / Higher National Certificate (HNC) or equivalent, other professional qualifications; (3) Education to age 16 qualifications: General Certificate of Education Ordinary Level (O levels) / General Certificate of Secondary Education (GCSEs) or equivalent, Certificate of Secondary Education (CSEs) or equivalent; (4) No qualifications) and gross annual household income (< £18,000, £18,000–£30,999, £31,000–£51,999, £52,000–£100,000 or > £100,000).

We also included the Index of Multiple Deprivation for England which is a small-area level measure derived by government from data on income, employment, health and disability, education skills and training, barriers to housing and services, living environment and crime [21]. Higher values on the index reflect greater deprivation.

#### Psychosocial factors

Loneliness was assessed using two questions: “Do you often feel lonely?” (no = 0 / yes = 1) and “How often are you able to confide in someone close to you?” (almost daily to about once a month = 0 / once every few months to never or almost never = 1). Individuals who received a sum score of 2 were classified as lonely [22].

Social isolation was assessed using three questions: “Including yourself, how many people are living together in your household?” (living alone = 1), “How often do you visit friends or family or have them visit you?” (less than once a month = 1) and “Which of the following [leisure/social activities] do you attend once a week or more often?” (none of the above = 1). Individuals who received a sum score of 2 or 3 were classified as socially isolated [22].

#### Lifestyle factors

Smoking status was assessed using two questions summarising current and past smoking behaviour. Individuals who responded “Yes, on most or all days” or “Only occasionally” to current tobacco smoking were coded as “current”. Individuals who responded “Smoked on most or all days” or “Smoked occasionally” to past tobacco smoking were coded as “former”. Individuals who responded “No” to current tobacco smoking and “Just tried once or twice” or “I have never smoked” to past tobacco smoking were coded as “never”.

Alcohol intake frequency was assessed using one question: “About how often do you drink alcohol?”. Response options included “Daily or almost daily”, “Three or four times a week”, “Once or twice a week”, “One to three times a month”, “Special occasions only” and “Never”.

Sleep duration was assessed using one question: “About how many hours sleep do you get in every 24 hours? (please include naps)”. Values below 1 h or above 23 h were rejected, and UK Biobank asked participants to confirm values below 3 h or above 12 h.

Body mass index (BMI) was calculated as weight divided by height squared (kg/m^2^). Weight measurements were obtained with a Tanita BC-418 MA body composition analyser. Standing height measurements were obtained using a Seca 202 height measure.

Physical activity was assessed using the International Physical Activity Questionnaire (IPAQ) short form [23]. Specifically, we included data on the number of days per week spent walking, engaging in moderate-intensity physical activity (e.g. “carrying light loads, cycling at normal pace”) or engaging in vigorous-intensity physical activity (i.e. “activities that make you sweat or breathe hard such as fast cycling, aerobics, heavy lifting”) for ≥ 10 min continuously.

Daily frequency of stair climbing was assessed using one question: “At home, during the last 4 weeks, about how many times a day do you climb a flight of stairs? (approx. 10 steps)”. These data were only collected from individuals who indicated that they were able to walk. Response options included “None”, “1–5 times a day”, “6–10 times a day”, “11–15 times a day”, “16–20 times a day” and “More than 20 times a day”.

#### Environmental exposures

Exposure to airborne pollutants was estimated using a Land Use Regression model developed as part of the European Study of Cohorts for Air Pollution Effects (ESCAPE) project [24, 25]. We included annual average concentration of particulate matter with an aerodynamic diameter of < 2.5 μm (PM_2.5_) and < 10 μm (PM_10_), as well as nitrogen dioxide (NO_2_) modelled at participants’ residential addresses for the year 2010.

Residential road traffic noise was modelled for the year 2009 using the Common Noise Assessment Methods (CNOSSOS-EU) algorithm [26, 27]. We used L_den_ (day-evening-night noise level) which is an annual average 24-h sound pressure level in decibels with a 10-decibel penalty added between 11 pm and 7 am. This penalty has previously been added in epidemiological analyses to account for annoyance / sleep disruption at night [28].

The percentage of the home location classed as greenspace, as a proportion of all land use types, was modelled using 2005 data from the Generalized Land Use Database for England (GLUD) [29] for the 2001 Census Output Areas in England. Each residential address was allocated a circular distance buffer of 1000 m, representing wider-area greenspace.

### Health status

Data on 81 cancer and 443 non-cancer illnesses (past and current) were ascertained through touchscreen self-report questionnaire and confirmed during a verbal interview by a trained nurse. In order to provide a single health indicator (“health status”) based on a previously defined algorithm, we used a classification developed by the Reinsurance Group of America (RGA) in which an experienced underwriter classified each illness according to whether it was “likely acceptable for standard life insurance” [30]. Participants were thus classified as healthy or unhealthy based on their reported cancer and non-cancer illnesses (Additional file 1: Table S1; Additional file 2). In developing this algorithm, the main determinant of whether to classify specific illnesses as healthy or unhealthy was their corresponding all-cause mortality risk. This classification does not account for the number of illnesses or temporality of diseases. In a separate analysis, we have shown that UK Biobank participants classified as unhealthy had a twofold increase in their risk of all-cause mortality compared to participants who were classified as healthy [31].

Two secondary health outcomes were assessed. Firstly, whether patients had a long-standing illness, disability or infirmity was assessed using the question “Do you have any long-standing illness, disability or infirmity?” to which individuals could respond “Yes” or “No”. Secondly, participants’ perceived health was assessed using the question “In general how would you rate your overall health?”. Response options included “Poor”, “Fair”, “Good” and “Excellent”. These health outcomes will be termed “long-standing illness” and “self-rated health”.

### Exclusion criteria

Women who were pregnant at the time of assessment were excluded from the analysis based on the assumption that lifestyle patterns change during pregnancy [32] (0.0003% of participants were excluded for this reason). Participants for whom their genetic sex, inferred from the relative intensity of biological markers on the Y and X chromosomes, and self-reported sex did not match were also excluded as this may reflect poor data quality (0.0007% of participants were excluded for this reason).

### Statistical analyses

Participants with baseline data on all explanatory variables and health indicators were selected for cross-sectional analyses. Participants with missing data or who responded “do not know” or “prefer not to answer” were excluded.

Logistic regression analyses were used to estimate associations between sociodemographic characteristics, psychosocial factors, lifestyle factors and environmental exposures with health status and long-standing illness. Ordinal logistic regression analyses were used to estimate associations between these factors and self-rated health (four categories). Across these outcomes, poor health was the reference group. In determining the reference groups for categorical explanatory variables, we focused on interpretability of the results for middle-aged and older UK residents.

For each explanatory variable, we fitted incrementally adjusted models using the baseline UK Biobank data: Model 1 included only individual explanatory variables; Model 2 included individual explanatory variables plus age and sex; Model 3 included all explanatory variables within a given domain (sociodemographic, psychosocial, lifestyle or environmental) plus age and sex; Model 4 was a full multivariable model that included all explanatory variables. All models were fitted in the analytical sample without missing data to ensure that any differences between models were not due to inclusion of different participants. We calculated odds ratios and Bonferroni-adjusted (~ 99.9%) confidence intervals. Adjusted *p* values were calculated to account for multiple testing within each model (i.e. typically for 39 tests). Two methods were used: (1) Bonferroni and (2) Benjamini & Hochberg [33], all two-tailed with *α* = .05, and false discovery rate of 5%, respectively. We report Bonferroni-adjusted *p* values for most results. For the interaction analyses (see below), we report in-text the most conservative correction at which statistical significance was reached (i.e. either Bonferroni or Benjamini & Hochberg) and present both sets of adjusted *p* values in Additional File 1. Multicollinearity was assessed using generalised variance inflation factors [34].

To compare the magnitudes of association between the explanatory variables and health indicators, we calculated standardised regression coefficients by rescaling all variables included in Model 4 to have a mean of zero and by dividing the coefficients of numeric variables with more than two values by twice their standard deviation [35].

To assess whether any associations between explanatory variables and health indicators were modified by sex or age, we stratified analyses by sex and by age at baseline assessment (< 65 years and ≥ 65 years) and assessed potential age and sex interactions by adding cross-product terms to Model 4. The choice of age strata was based on the current UK retirement age.

To assess whether the explanatory variables assessed at baseline predicted self-rated health at follow-up, we applied the same modelling strategy (ordinal logistic regression analyses) to the subsets of participants with repeat assessment data. We additionally adjusted for the number of days between the baseline and follow-up assessments in these analyses. This additional adjustment was included to account for the possibility that individuals who had their first or second revisit (t1 and t2, respectively) closer to the baseline assessment would be less likely to have had changes in their self-rated health.

As variable selection and classification might impact results, we conducted multiple additional analyses to examine related variables, categorised continuous variables according to health guidelines or previously determined cut-offs and tested the robustness of our findings to additional exclusion criteria. We repeated the main analysis (i) with the Townsend deprivation index instead of the Index of Multiple Deprivation for England as measure of neighbourhood deprivation; (ii) examining each individual component of the Index of Multiple Deprivation for England separately; (iii) with sleep duration as categorical variable (< 7, 7–8 [reference] and ≥ 9 h of sleep/day) [36]; (iv) with BMI as categorical variable (< 18.5, 18.5–24.9 [reference] and > 24.9 kg/m^2^); (v) with body fat percentage (estimated by electrical bio-impedance measurement) instead of BMI as measure of body composition; (vi) excluding individuals who reported that they stopped drinking alcohol because of “Illness or ill health” or “Doctor’s advice”; (vii) sub-dividing individuals who reported that they never drink alcohol into current and lifetime abstainers; (viii) with current tobacco smoking only (three categories: “Yes, on most or all days”, “Only occasionally” and “No”); (ix) with Metabolic Equivalent Task (MET) minutes per week for walking, moderate physical activity and vigorous physical activity [37] instead of the number of days per week spent engaging in these activities for ≥ 10 min; (x) reducing the greenspace percentage circular distance buffer from 1000 to 300 m to examine nearby greenspace; (xi) with air pollution and noise exposure estimates dichotomised according to WHO recommendation thresholds [38, 39]: PM_2.5_ ≤ 10 μg/m^3^, PM_10_ ≤ 20 μg/m^3^, NO_2_ ≤ 40 μg/m^3^ and L_den_ ≤ 53 decibels; (xii) restricting analyses to participants assessed after 31 December 2008 (regarding noise estimates) and restricting analyses to participants assessed after 31 December 2009 (regarding air pollution estimates); (xiii) restricting analyses to those individuals who had lived at their current address for at least 10 years; (xiv) truncating continuous explanatory variables at the 1st and 99th percentile of the distribution.

Statistical analyses were conducted using R (version 3.6.0).

## Results

### Study population

Of 502,521 UK Biobank participants, 5.32% (*n* = 26,757) had missing health data and 33.51% (*n* = 168,386) had missing data on explanatory variables or did not meet our inclusion criteria. Hence our analytical sample included *n* = 307,378 adults. Missing data for each explanatory variable are described in Additional file 1: Fig. S1.

Descriptive statistics are presented in Additional file 1: Tables S2–S5. There were few differences between the full and analytical sample, with the analytical sample slightly healthier and more educated. Of the participants in the analytical sample, 8.18% (*n* = 25,142) reported at least one cancer and 73.30% (*n* = 225,312) at least one non-cancer illness (Additional file 1: Fig. S2 and S3). Approximately two thirds of participants (69.04%, *n* = 212,201) were classified as healthy, while 30.96% (*n* = 95,177) were unhealthy. A similar percentage of participants (30.5%, *n* = 93,757) reported having a long-standing illness (72.9% agreement with health status). Finally, 3.60% (*n* = 11,066) rated their health as poor, while 19.25% (*n* = 59,169), 59.44% (*n* = 182,699) and 17.71% (*n* = 54,444) rated their health as fair, good and excellent, respectively.

### Regression analyses

We found weak correlations between most continuous explanatory variables and moderate to strong correlations between the environmental exposures (Additional file 1: Fig. S4). Generalised variance inflation factor (VIF) values for Model 4 were between 1.02 and 2.62 for 19/21 explanatory variables. The VIFs for NO_2_ and PM_2.5_ were 6.08 and 4.27, respectively. Excluding NO_2_ from the model reduced the highest VIF to 2.42 (for PM_2.5_). Fitting Model 4 without NO_2_ or with each air pollutant separately had little impact on their associations with health status. As such, we report the results for Model 4 that included all explanatory variables. Unless indicated otherwise, results presented below correspond to multivariable-adjusted odds ratios and Bonferroni-adjusted (~ 99.9%) confidence intervals from Model 4. A simplified overview of our findings is presented in Additional file 1: Tables S6–S9.

Increased age was associated with lower odds of favourable health status (OR = 0.953, 99.9% CI 0.951–0.955, *p*_Bonf._ < 0.001). Men had lower odds of being healthy (OR = 0.88, 99.9% CI 0.86–0.91, *p*_Bonf._ < 0.001). Individuals with a high income had higher odds of being healthy (OR = 1.05, 99.9% CI 1.01–1.09, *p*_Bonf._ = 0.003 [£52,000–£100,000 vs £31,000–£51,999]), while those with lower levels of income had lower odds of being healthy (e.g. OR = 0.74, 99.9% CI 0.71–0.78, *p*_Bonf._ < 0.001 [< £18,000 vs £31,000–£51,999]). Increased neighbourhood deprivation was associated with lower odds of being healthy (OR = 0.995, 99.9% CI 0.994–0.996, *p*_Bonf._ < 0.001). Compared to Whites, individuals of Black, Chinese, Mixed-race, and “other” ethnic background had higher odds of being healthy in Model 1, but only Chinese ethnicity was associated with higher odds of being healthy across all models (Model 4: OR = 1.83, 99.9% CI 1.36–2.51, *p*_Bonf._ < 0.001). Compared to individuals without educational or professional qualification, participants with any qualification had higher odds of being healthy in Model 1, after adjustment for age and sex (Model 2) and, except for A levels or equivalent education level, in Model 3 that included all sociodemographic characteristics. However, there was only limited evidence of associations between participants’ qualifications and health in Model 4 (Table 1).

**Table 1:** Sociodemographic characteristics and psychosocial factors associated with health status

	Model 1			Model 2			Model 3			Model 4		
Term	OR	Bonferroni-corrected CI		OR	Bonferroni-corrected CI		OR	Bonferroni-corrected CI		OR	Bonferroni-corrected CI	
*Sociodemographic characteristics*												
**Household income**^1^												
Very low	0.5161	0.4978	0.5351	0.6298	0.6066	0.6538	0.6825	0.6558	0.7102	0.7447	0.7148	0.7759
Low	0.7548	0.7288	0.7818	0.8728	0.8419	0.9048	0.8963	0.8643	0.9295	0.9196	0.8864	0.9541
Middle	Ref	–	–	Ref	–	–	Ref	–	–	Ref	–	–
High	1.2084	1.1626	1.2561	1.1016	1.0591	1.1458	1.0774	1.0354	1.1212	1.0510	1.0096	1.0941
Very high	1.2867	1.2088	1.3703	1.1483	1.0778	1.2239	1.1151	1.0457	1.1897	1.0472	0.9812	1.1181
**Sex**												
Female	Ref	–	–	Ref	–	–	Ref	–	–	Ref	–	–
Male	0.8642	0.8428	0.8862	NA	NA	NA	0.8810	0.8584	0.9043	0.8847	0.8610	0.9090
**Age**	0.9462	0.9446	0.9478	NA	NA	NA	0.9527	0.9510	0.9545	0.9528	0.9510	0.9546
**Multiple deprivation**	0.9912	0.9903	0.9921	0.9885	0.9876	0.9894	0.9923	0.9913	0.9933	0.9952	0.9941	0.9963
**Ethnicity**												
White	Ref	–	–	Ref	–	–	Ref	–	–	Ref	–	–
Mixed-race	1.2991	1.0925	1.5524	0.9743	0.8163	1.1684	1.0443	0.8742	1.2533	1.1037	0.9221	1.3273
Asian	1.0196	0.9213	1.1298	0.8560	0.7714	0.9511	0.9252	0.8333	1.0285	1.0188	0.9149	1.1359
Black	1.2994	1.1612	1.4571	0.9831	0.8764	1.1050	1.1929	1.0619	1.3426	1.3125	1.1657	1.4805
Chinese	2.1528	1.6132	2.9286	1.7413	1.2994	2.3777	1.7988	1.3413	2.4580	1.8339	1.3646	2.5106
Other	1.2077	1.0369	1.4115	0.9952	0.8519	1.1667	1.1096	0.9491	1.3018	1.2570	1.0727	1.4779
**Highest qualification**												
None	Ref	–	–	Ref	–	–	Ref	–	–	Ref	–	–
O levels/GCSEs/CSEs	1.6519	1.5859	1.7207	1.2266	1.1757	1.2796	1.0692	1.0236	1.1168	1.0315	0.9870	1.0779
A levels/NVQ/HND/HNC^2^	1.5125	1.4508	1.5769	1.2102	1.1595	1.2631	1.0270	0.9825	1.0735	0.9879	0.9445	1.0332
Degree	1.7949	1.7257	1.8667	1.3373	1.2838	1.3929	1.0469	1.0018	1.0940	0.9558	0.9137	0.9997
*Psychosocial factors*												
**Loneliness**												
Not lonely	Ref	–	–	Ref	–	–	Ref	–	–	Ref	–	–
Lonely	0.7283	0.6915	0.7672	0.6947	0.6588	0.7326	0.7207	0.6832	0.7604	0.8129	0.7697	0.8587
**Social isolation**												
Not isolated	Ref	–	–	Ref	–	–	Ref	–	–	Ref	–	–
Isolated	0.7544	0.7226	0.7877	0.7542	0.7218	0.7882	0.7795	0.7457	0.8149	0.9496	0.9067	0.9947

*Note:* Bonferroni-adjusted (~ 99.9%) confidence intervals. *OR* odds ratio, *CI* confidence interval, *GCSEs* general certificate of secondary education, *CSE* certificate of secondary education, *NVQ* national vocational qualification, *HND* higher national diploma, *HNC* higher national certificate, *NA* not applicable. ^1^Annual household income groups: very low (< £18,000), low (£18,000–£30,999), middle (£31,000–£51,999), high (£52,000–£100,000) and very high (> £100,000). ^2^also includes “other professional qualifications”

Model 1—only individual explanatory variables

Model 2—adjusted for age and sex

Model 3—age, sex and all sociodemographic characteristics / age, sex and all psychosocial factors

Model 4—all explanatory variables

Loneliness was associated with lower odds of favourable health status (OR = 0.81, 99.9% CI 0.77–0.86, *p*_Bonf._ < 0.001). Socially isolated individuals also had lower odds of being healthy, but the strength of association was weaker than for loneliness in Model 4 (OR = 0.95, 99.9% CI 0.91–0.99, *p*_Bonf._ = 0.01) (Table 1).

Longer sleep duration was associated with lower odds of favourable health status (OR = 0.97, 99.9% CI 0.96–0.99, *p*_Bonf._ < 0.001). Walking frequently and engaging in frequent vigorous physical activity was associated with higher odds of being healthy across most analyses (OR = 1.010, 99.9% CI 1.003–1.018, *p*_Bonf._ < 0.001 and OR = 1.03, 99.9% CI 1.02–1.04, *p*_Bonf._ < 0.001, respectively). Although moderate physical activity was associated with higher odds of being healthy in Models 1 and 2, we did not find evidence of an association in Models 3 and 4 (OR = 1.003, 99.9% CI 0.996–1.010, *p*_Bonf_ > 0.99, Model 4). Frequent daily stair climbing was associated with higher odds of being healthy (ranging from OR = 1.07, 99.9% CI 1.01–1.13, *p*_Bonf._ = 0.002 to OR = 1.19, 99.9% CI 1.11–1.27, *p*_Bonf._ < 0.001 [1–5 times/day and > 20 times/day vs none, respectively]). We found some evidence that frequent alcohol intake was associated with higher odds of being healthy (e.g. OR = 1.06, 99.9% CI 1.02–1.10, *p*_Bonf._ < 0.001 [3–4 vs 1–2 times/week]), although for daily/almost daily alcohol drinking only in Models 2 and 3 (OR = 1.06, 99.9% CI 1.02–1.10, *p*_Bonf._ < 0.001, Model 2), while infrequent alcohol intake was associated with lower odds of being healthy (ranging from OR = 0.91, 99.9% CI 0.87–0.96, *p*_Bonf._ < 0.001 to OR = 0.63, 99.9% CI 0.59–0.66, *p*_Bonf._ < 0.001 [1–3 times/month and never vs 1–2 times/week, respectively]). Higher BMI was associated with lower odds of being healthy (OR = 0.968, 99.9% CI 0.966–0.971, *p*_Bonf._ < 0.001). Past and current tobacco smoking was associated with lower odds of being healthy (OR = 0.79, 99.9% CI 0.77–0.82, *p*_Bonf._ < 0.001 and OR = 0.75, 99.9% CI 0.72–0.79, *p*_Bonf._ < 0.001, respectively) (Table 2).

**Table 2:** Lifestyle factors associated with health status

	Model 1			Model 2			Model 3			Model 4		
Term	OR	Bonferroni-corrected CI		OR	Bonferroni-corrected CI		OR	Bonferroni-corrected CI		OR	Bonferroni-corrected CI	
**Sleep duration** (hours/day)	0.9574	0.9460	0.9688	0.9817	0.9699	0.9937	0.9743	0.9626	0.9862	0.9748	0.9631	0.9868
**Physical activity** (days/week)^1^												
Walking	1.0099	1.0034	1.0164	1.0258	1.0190	1.0326	1.0059	0.9987	1.0131	1.0105	1.0032	1.0178
Moderate activity	1.0095	1.0040	1.0149	1.0253	1.0197	1.0310	0.9995	0.9928	1.0062	1.0032	0.9964	1.0100
Vigorous activity	1.0571	1.0502	1.0642	1.0502	1.0431	1.0573	1.0347	1.0267	1.0429	1.0319	1.0238	1.0400
**Stair climbing frequency**												
None	Ref	–	–	Ref	–	–	Ref	–	–	Ref	–	–
1–5/day	1.3009	1.2362	1.3688	1.0980	1.0422	1.1568	1.1045	1.0477	1.1643	1.0704	1.0148	1.1289
6–10/day	1.5529	1.4813	1.6278	1.3354	1.2725	1.4013	1.2467	1.1872	1.3091	1.1756	1.1188	1.2352
11–15/day	1.6271	1.5457	1.7128	1.3923	1.3211	1.4672	1.2503	1.1855	1.3186	1.1714	1.1098	1.2363
16–20/day	1.6494	1.5530	1.7520	1.4123	1.3280	1.5020	1.2433	1.1681	1.3235	1.1650	1.0937	1.2410
20+/day	1.7494	1.6390	1.8675	1.4402	1.3474	1.5396	1.2632	1.1806	1.3518	1.1881	1.1097	1.2723
**Alcohol intake frequency**												
Never	0.5651	0.5364	0.5954	0.5849	0.5545	0.6171	0.5944	0.5632	0.6275	0.6285	0.5946	0.6644
Special occasions	0.7015	0.6705	0.7341	0.7109	0.6785	0.7448	0.7450	0.7107	0.7809	0.7792	0.7429	0.8174
1–3/month	0.9082	0.8678	0.9505	0.8730	0.8333	0.9146	0.8947	0.8537	0.9378	0.9129	0.8708	0.9570
1–2/week	Ref	–	–	Ref	–	–	Ref	–	–	Ref	–	–
3–4/week	1.0652	1.0271	1.1047	1.1059	1.0656	1.1478	1.0882	1.0481	1.1298	1.0590	1.0198	1.0998
Daily/almost daily	0.9293	0.8957	0.9643	1.0627	1.0232	1.1037	1.0744	1.0339	1.1166	1.0334	0.9939	1.0745
**BMI** (kg/m^2^)	0.9561	0.9536	0.9587	0.9580	0.9554	0.9606	0.9659	0.9632	0.9687	0.9683	0.9656	0.9711
**Smoking status**												
Never	Ref	–	–	Ref	–	–	Ref	–	–	Ref	–	–
Former	0.6977	0.6792	0.7168	0.7879	0.7663	0.8100	0.7765	0.7548	0.7989	0.7922	0.7698	0.8152
Current	0.7309	0.7003	0.7629	0.6850	0.6556	0.7158	0.6882	0.6583	0.7196	0.7518	0.7183	0.7869

*Note:* Bonferroni-adjusted (~ 99.9%) confidence intervals. *OR* odds ratio, *CI* confidence interval, *BMI* body mass index. ^1^number of days per week engaging in these activities for 10+ min continuously

Model 1—only individual explanatory variables

Model 2—adjusted for age and sex

Model 3—age, sex and all lifestyle factors

Model 4—all explanatory variables

In our analyses of environmental exposures, higher PM_2.5_ concentration was associated with lower odds of favourable health status (OR = 0.97, 99.9% CI 0.94–0.99, *p*_Bonf._ < 0.001). PM_10_ was also associated with lower odds of being healthy in Models 1 and 2, but there was no evidence of an association between PM_10_ and health status in Models 3 and 4 (OR = 1.00, 99.9% CI 0.99–1.01, *p*_Bonf._ > 0.99, Model 4). NO_2_ was associated with lower odds of being healthy in Models 1 and 2. However, in Models 3 and 4, there was no evidence that NO_2_ was associated with health status after Bonferroni correction (OR = 1.004, 99.9% CI 0.999–1.008, *p*_Bonf._ = 0.27, Model 4). Ambient sound level was associated with lower odds of being healthy in Models 1 and 2, but there was no evidence of an association with health status in Models 3 and 4 (OR = 0.998, 99.9% CI 0.995–1.002, *p*_Bonf._ > 0.99, Model 4). Finally, we did not find consistent evidence of an association between percentage greenspace within a 1000 m circular distance buffer and health status (Table 3).

**Table 3:** Environmental exposures associated with health status

	Model 1			Model 2			Model 3			Model 4		
Term	OR	Bonferroni-corrected CI		OR	Bonferroni-corrected CI		OR	Bonferroni-corrected CI		OR	Bonferroni-corrected CI	
**PM**_**2.5**_	0.9640	0.9525	0.9757	0.9357	0.9243	0.9473	0.9159	0.8933	0.9392	0.9656	0.9411	0.9908
**PM**_**10**_	0.9917	0.9851	0.9983	0.9842	0.9775	0.9909	1.0048	0.9965	1.0132	1.0019	0.9935	1.0104
**NO**_**2**_	0.9972	0.9955	0.9988	0.9929	0.9912	0.9945	1.0000	0.9959	1.0041	1.0036	0.9993	1.0078
**L**_**den**_	0.9967	0.9938	0.9996	0.9945	0.9916	0.9975	1.0002	0.9967	1.0038	0.9981	0.9945	1.0017
**Greenspace** 1000 m	1.0001	0.9995	1.0006	1.0013	1.0007	1.0019	0.9988	0.9978	0.9997	0.9995	0.9985	1.0004

*Note:* Bonferroni-adjusted (~ 99.9%) confidence intervals. *OR* odds ratio, *CI* confidence interval, *PM* particulate matter, *NO*_*2*_ nitrogen dioxide, *L*_*den*_ day-evening-night noise level

Model 1—only individual explanatory variables

Model 2—adjusted for age and sex

Model 3—age, sex and all environmental exposures

Model 4—all explanatory variables

**Fig. 1:**
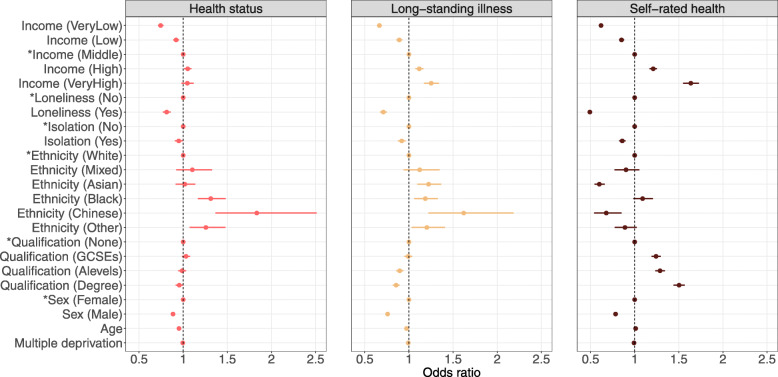
Sociodemographic characteristics and psychosocial factors associated with health indicators. Confidence interval plot (odds ratio ± Bonferroni-adjusted (~ 99.9%) confidence intervals) for Model 4 (i.e. including all explanatory variables). GCSEs = general certificate of secondary education. ^*^Indicates reference group for categorical explanatory variables. For categorical explanatory variables the odds ratios for self-rated health indicate the changes in odds of reporting better self-rated health associated with the explanatory variable group relative to the reference group. Odds ratios for continuous explanatory variables for self-rated health indicate proportional odds ratios for a 1-unit increase in the explanatory variable on level of self-rated health. Annual household income groups: very low (< £18,000), low (£18,000–£30,999), middle (£31,000–£51,999), high (£52,000–£100,000) and very high (> £100 000). “GCSEs” also includes O levels and certificate of secondary education (CSE). “A levels” also includes national vocational qualification (NVQ), higher national diploma (HND), higher national certificate (HNC) and “other professional qualifications”

**Fig. 2:**
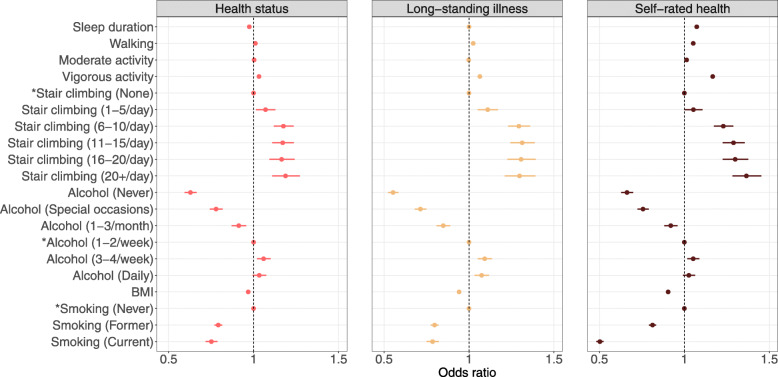
Lifestyle factors associated with health indicators. Confidence interval plot (odds ratio ± Bonferroni-adjusted (~ 99.9%) confidence intervals) for Model 4 (i.e. including all explanatory variables). BMI = body mass index. ^*^Indicates reference group for categorical explanatory variables. For categorical explanatory variables the odds ratios for self-rated health indicate the changes in odds of reporting better self-rated health associated with the explanatory variable group relative to the reference group. Odds ratios for continuous explanatory variables for self-rated health indicate proportional odds ratios for a 1-unit increase in the explanatory variable on level of self-rated health

**Fig. 3:**
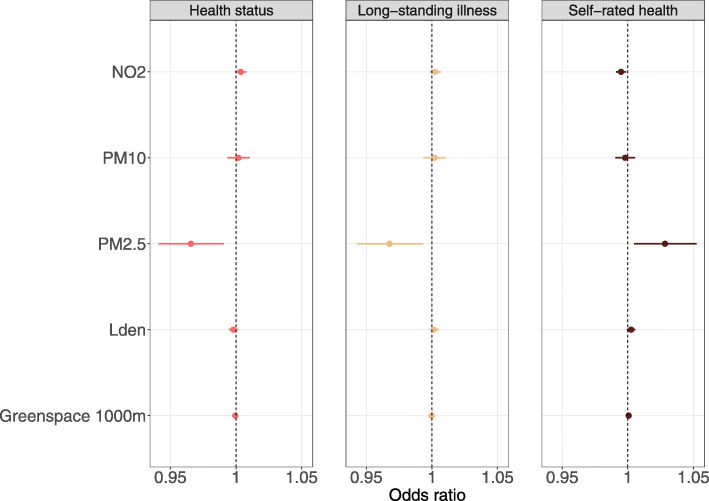
Environmental exposures associated with health indicators. Confidence interval plot (odds ratio ± Bonferroni-adjusted (~ 99.9%) confidence intervals) for Model 4 (i.e. including all explanatory variables). PM = particulate matter; NO_2_ = nitrogen dioxide; L_den_ = day-evening-night noise level. Odds ratios for self-rated health indicate proportional odds ratios for a 1-unit increase in the explanatory variable on level of self-rated health

Full results for long-standing illness and self-rated health are presented in Additional file 1: Tables S10–S21. Model 4 results across health indicators are presented in Figs. 1, 2 and 3.

Findings regarding income, sex and neighbourhood deprivation were mostly consistent across health indicators, although with some variation in the magnitude of associations. Compared to Whites, participants of Chinese ethnicity had lower odds of favourable self-rated health (OR = 0.68, 99.9% CI 0.54–0.85, *p*_Bonf._ < 0.001) but had higher odds of being classified healthy (OR = 1.83, 99.9% CI 1.36–2.51, *p*_Bonf._ < 0.001) and being free from long-standing illness (OR = 1.62, 99.9% CI 1.22–2.18, *p*_Bonf._ < 0.001). Individuals of non-White ethnic backgrounds tended to rate their health less favourable, but there was some evidence that they had higher odds of being free from long-standing illness, especially in the full multivariable model. Individuals with any qualification had higher odds of rating their health more favourable (e.g. OR = 1.50, 99.9% CI 1.44–1.57, *p*_Bonf._ < 0.001 [university/college degree vs no qualification]), while results were less consistent for health status and long-standing illness, especially in Models 3 and 4. Finally, older individuals tended to rate their health more favourable (OR = 1.011, 99.9% CI 1.010–1.013, *p*_Bonf._ < 0.001).

Findings regarding psychosocial factors were consistent across health indicators, but the association was strongest for loneliness and self-rated health (OR = 0.49, 99.9% CI 0.47–0.52, *p*_Bonf._ < 0.001).

Findings regarding lifestyle factors were consistent across health indicators, except that there were some inconsistencies in the associations between daily/almost daily alcohol intake and health. More frequent moderate physical activity was associated with better self-rated health also in Model 4 (OR = 1.01, 99.9% CI 1.01–1.02, *p*_Bonf._ < 0.001). Longer sleep duration was associated with better self-rated health (OR = 1.07, 99.9% CI 1.06–1.09, *p*_Bonf._ < 0.001), but there was little evidence of an association with having a long-standing illness.

Higher levels of PM_2.5_ were also associated with lower odds of being free from long-standing illness (OR = 0.97, 99.9% CI 0.94–0.99, *p*_Bonf._ = 0.002). While we found similar associations with self-rated health in Models 1–3, PM_2.5_ was associated with better self-rated health in Model 4 (OR = 1.028, 99.9% CI 1.005–1.052, *p*_Bonf._ = 0.004). There was no evidence of an association between PM_10_ and any health indicator in Models 3 and 4, although PM_10_ was associated with poor health across outcomes in Models 1 and 2. Higher NO_2_ concentration was associated with less favourable self-rated health (OR = 0.995, 99.9% CI 0.991–0.999, *p*_Bonf._ < 0.001), while there was no evidence of an association with health status and having a long-standing illness after adjusting for covariates in Models 3 and 4. Higher L_den_ was associated with poor health in Models 1 and 2, but we found no evidence of an association in Model 4. For long-standing illness and self-rated health, there was some evidence that higher L_den_ was associated with better health in Model 3. Percentage greenspace was associated with better self-rated health and higher odds of being free from long-standing illness in Models 1 and 2. Results from Model 3 suggested that greenspace was associated with less favourable health across all health indicators, but we found no evidence of any associations in Model 4 after Bonferroni correction.

### Magnitude of associations

**Fig. 4:**
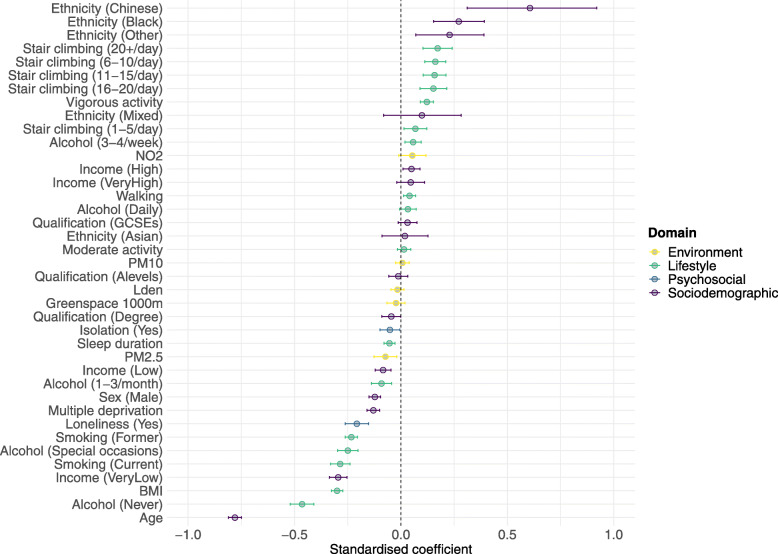
Confidence interval plot for health status. Standardised *β* estimates and Bonferroni-adjusted (~ 99.9%) confidence intervals. Model 4 regression coefficients were rescaled to have a mean equal to zero and, for numeric variables with more than two values, divided by two standard deviations

Standardised regression coefficients for health status are presented in Fig. 4. Most notably, the magnitude of association between BMI (*β* = *−*0.30, 99.9% CI −0.33 to −0.27) or being on a very low income (*β* = *−*0.29, 99.9% CI −0.34 to −0.25) and health status were comparable to that of current smoking (*β* = *−*0.29, 99.9% CI −0.33 to −0.24). There was also a substantial difference in the magnitude of association with health status between the very low and low income groups (*β* = *−*0.29, 99.9% CI −0.34 to −0.25, and *β* = *−*0.08, 99.9% CI −0.12 to −0.05, respectively). The magnitude of association between loneliness and health status was substantially larger than that of social isolation (*β* = *−*0.21, 99.9% CI −0.26 to −0.15, and *β* = *−*0.05, 99.9% CI −0.10 to −0.01, respectively). Finally, associations between the environmental exposures and health status were relatively small compared to those observed for the sociodemographic, psychosocial and lifestyle factors (Additional file 1: Tables S22).

Associations with long-standing illness and self-rated health compared to health status were stronger for household income, sex, neighbourhood deprivation, loneliness, social isolation, walking frequency, vigorous physical activity, BMI and stair climbing frequency (Additional file 1: Tables S23 and S24, Fig. S5 and S6).

### Stratified and interaction analyses

Descriptive statistics of the analytical sample stratified by sex and age group are presented in Additional file 1: Tables S25 and S26. Findings presented below correspond to results for which the stratified and interaction analyses provided consistent conclusions. Full results and effect sizes are presented in Additional file 1: Tables S27–S50, Fig. S7–S24.

The odds of being healthy were lower for men than for women in the lowest income group (*p*_Bonf.(interaction)_ < 0.001). The association between increased age and lower odds of being healthy was stronger in men (*p*_Bonf.(interaction)_ < 0.001). Asian women had higher odds of being healthy (*p*_BH(interaction)_ = 0.007). The association between neighbourhood deprivation and lower odds of being healthy was stronger in men (*p*_Bonf.(interaction)_ < 0.001). Men who were lonely had lower odds of being healthy (*p*_Bonf.(interaction)_ = 0.008) and social isolation was associated with lower odds of being healthy only in men (*p*_BH(interaction)_ = 0.006). Longer sleep duration was associated with lower odds of being healthy only in men (*p*_Bonf.(interaction)_ < 0.001). Reporting frequent alcohol intake was associated with higher odds of being healthy only in men (*p*_BH(interaction)_ = 0.023 for 3–4 times/week and *p*_BH(interaction)_ = 0.039 for daily/almost daily). The association between higher BMI and lower odds of being healthy was stronger in men (*p*_Bonf.(interaction)_ < 0.001). The association between former smoking and health status was stronger in men (*p*_Bonf.(interaction)_ < 0.001), while the association between current smoking and health status was stronger in women (*p*_BH(interaction)_ = 0.005). PM_2.5_ was associated with lower odds of being healthy only in men (*p*_BH(interaction)_ = 0.011). Finally, we found that NO_2_ was associated with higher odds of favourable health status only in women (*p*_BH(interaction)_ = 0.024) (Additional file 1: Tables S27–S30, Fig. S7–S9).

The association between being on a very low income and lower odds of being healthy was stronger in participants below the age of 65 than in participants aged 65 and above (*p*_Bonf.(interaction)_ < 0.001). Reporting an income of £52,000–£100,000 was associated with higher odds of being healthy only in participants younger than 65 (*p*_BH(interaction)_ = 0.016). Men aged 65 and above had lower odds of being healthy (*p*_Bonf.(interaction)_ < 0.001). Loneliness and social isolation were associated with lower odds of being healthy only in individuals younger than 65 (*p*_BH(interaction)_ = 0.024 and *p*_Bonf.(interaction)_ = 0.011, respectively). The association between walking frequency and higher odds of being healthy was stronger in individuals aged 65 and above (*p*_Bonf.(interaction)_ < 0.001). Climbing stairs 1–5 times/day was associated with favourable health status only in individuals younger than 65 (*p*_Bonf.(interaction)_ < 0.001). There was some evidence that the association between never drinking alcohol and poor health status was stronger in individuals younger than 65 (*p*_Bonf.(interaction)_ = 0.002). The association between higher BMI and lower odds of being healthy was stronger in individuals aged 65 and above (*p*_BH(interaction)_ < 0.001). Finally, the association between current smoking and poor health status was stronger in individuals younger than 65 (*p*_BH(interaction)_ = 0.038) (Additional file 1: Tables S31–S34, Fig. S10–S12).

Although there were some differences, stratified and interaction analyses of long-standing illness and self-rated health indicated a large degree of consistency with the results observed for health status (Additional file 1: Tables S35–S50, Fig. S13–S24).

### Longitudinal analyses

We repeated our analyses of self-rated health in participants with follow-up data collected between 2012 and 2013 (*n* = 16,058; mean follow-up = 4.26 years, SD = 0.87) and between 2014 and 2019 (*n* = 32,617; mean follow-up = 8.57 years, SD = 1.64). Descriptive statistics and full results are presented in Additional file 1: Tables S51–S59. The findings were fully consistent with cross-sectional analyses for very low, high and very high levels of income, sex, loneliness, sleep duration, walking frequency, vigorous physical activity, infrequent alcohol intake, BMI and smoking status. Although many results were consistent across timepoints, we found some differences between cross-sectional and longitudinal analyses for low income, age, neighbourhood deprivation, ethnicity, highest qualification, social isolation, moderate physical activity, stair climbing frequency, regular alcohol intake and the environmental exposures.

### Additional analyses

Additional descriptive statistics are presented in Additional file 1: Table S60.

Repeating the main analysis with the Townsend deprivation index as measure of neighbourhood deprivation that does not include a health dimension led to similar conclusions as for the Index of Multiple Deprivation for England (OR = 0.974, 99.9% CI 0.969–0.980, *p*_Bonf._ < 0.001). We also found that higher scores on each individual component of the Index of Multiple Deprivation for England, except for the housing dimension, were associated with lower odds of favourable health status (results not shown).

For sleep duration and BMI, we found evidence of non-linearity in the association with health status (Additional file 1: Fig. S25). Hence, we also examined these explanatory variables as categorical variables. Compared to individuals with optimal sleep duration (7–8 h/day), those who slept < 7 or ≥ 9 h/day had lower odds of being healthy (OR = 0.90, 99.9% CI 0.87–0.92, *p*_Bonf._ < 0.001 and OR = 0.70, 99.9% CI 0.67–0.74, *p*_Bonf._ < 0.001, respectively). We also found that low (< 18.5 kg/m^2^) and high (> 24.9 kg/m^2^) BMI was associated with lower odds of being healthy, compared to a BMI of 18.5–24.9 kg/m^2^ (OR = 0.70, 99.9% CI 0.58–0.85, *p*_Bonf._ < 0.001 and OR = 0.85, 99.9% CI 0.83–0.88, *p*_Bonf._ < 0.001, respectively). There was no evidence of substantial departure from a linear association with health status for all other continuous variables (Additional file 1: Fig. S25). Repeating the main analysis with body fat percentage as measure of body composition led to similar conclusions as for BMI: higher fat percentage was associated with lower odds of being healthy (*n* = 307,202; OR = 0.978, 99.9% CI 0.975–0.980, *p*_Bonf._ < 0.001). Excluding individuals who reported that they had stopped drinking alcohol due to illness or their doctor’s advice (*n* = 2790) attenuated the association between never drinking alcohol and health status (*n* = 304,588; OR = 0.74, 99.9% CI 0.70–0.79, *p*_Bonf._ < 0.001). When sub-dividing individuals who reported that they never drink alcohol into current and lifetime abstainers, the association with health status was stronger in current abstainers (*n* = 307,346; OR = 0.53, 99.9% CI 0.49–0.57, *p*_Bonf._ < 0.001 and OR = 0.75, 99.9% CI 0.70–0.81, *p*_Bonf._ < 0.001, respectively). Restricting analyses to current smoking, we found that regular smoking and smoking only occasionally was associated with lower odds of being healthy, although the magnitude of association was stronger for regular smoking (*n* = 307,372; OR = 0.81, 99.9% CI 0.77–0.85, *p*_Bonf._ < 0.001 and OR = 0.90, 99.9% CI 0.83–0.98, *p*_Bonf._ = 0.002, respectively). Examining MET minutes instead of the number of days per week spent walking, engaging in moderate or vigorous physical activity led to similar results (*n* = 268,674; data not shown).

When we examined air pollution and noise exposure estimates dichotomised according to WHO recommendation thresholds, we found no evidence that being exposed to ≤ 10 μg/m^3^ PM_2.5_ on average annually was associated with higher odds of being healthy after Bonferroni correction (OR = 1.029, 99.9% CI 0.997–1.062, *p*_Bonf._ = 0.15). There was also no evidence of an association with health status for exposures to ≤ 20 μg/m^3^ PM_10_, ≤ 40 μg/m^3^ NO_2_ or ≤ 53 decibels L_den_ in Model 4. We also did not find evidence of a threshold in the association between PM_10_ or NO_2_ and health status when including quintiles of these exposures in Model 4 (data not shown). Restricting analyses to participants assessed after 2008 (regarding noise estimates; *n* = 182,342) or 2009 (regarding air pollution estimates; *n* = 60,521) or to those who had lived at their current address for at least 10 years (*n* = 209,239) did not lead to different conclusions regarding the associations between environmental exposures and health status. There was no evidence that percentage greenspace within a 300-m circular distance buffer was associated with health status after Bonferroni correction (*n* = 307,378; OR = 0.9993, 99.9% CI 0.9985–1.0001, *p*_Bonf._ = 0.29). Truncating continuous explanatory variables at the 1st and 99th percentile did not materially change our results (*n* = 278,065; data not shown).

## Discussion

Increased age was strongly associated with unfavourable health status and having a long-standing illness. However, older participants generally rated their health positively, which is consistent with several smaller studies [40, 41], though not all [42, 43]. Ageing might be the single most important factor underlying disease [44], with an almost universally accepted expectation of declining health as people get older. As attainable health states shift with age [45], older participants might evaluate their health more favourable, despite higher rates of illness and disability.

Although women, on average, report more illnesses, disabilities and limitations in daily life [46–48], one of the most robust findings in human biology is that they live longer than men [49]. Our findings are consistent with results from the Newcastle 85+ cohort study in which women rated their health more favourably than men [46]. However, other studies reported that women rated their health less favourably than men [50–52], or did not find evidence of sex differences in self-rated health [53, 54]. Sex differences in health status could result from differences in the frequency of specific illnesses, sex-specific reporting patterns, or biological and social factors [52]. Discrepancies in findings between studies might reflect differences in age group or socio-cultural factors.

High income and low levels of neighbourhood deprivation were associated with better health, which is broadly consistent with previous studies [55–57]. Notably, we found only a small difference in the strength of association with health status and having a long-standing illness between the high-income groups. The difference between the low-income groups, however, was substantial, supporting previous findings, which suggested a non-linear association between family income and mortality [58]. For self-rated health, we also found evidence of substantial differences between the high-income groups. A possible explanation for the observation that there was less evidence of associations between household income and health in individuals aged 65 and over than in the younger age group is that more individuals in this age group received a pension income. Future studies could examine associations between health and related socioeconomic variables such as family income or household income per capita.

Our study provides limited evidence that education was independently associated with favourable health status after accounting for other factors, although higher levels of qualification remained associated with better self-rated health, consistent with previous research [42]. A recent UK Biobank analysis showed that remaining longer in school causally reduced participants’ risk of diabetes and mortality [59]. A potential explanation for why we did not find a consistent pattern in the full model is that most differences in health status result from educated individuals engaging in healthier lifestyle behaviours [60] that we had accounted for, or they could be due to socioeconomic or genomic factors.

Social isolation and loneliness were associated with poor health. The strength of association was greater for loneliness, particularly in men and in individuals below the age of 65. Social isolation and loneliness were not always correlated [61, 62] and represent different aspects of social relations (scarcity of contact with others and discrepancies between the need for, and the fulfilment of, social interaction, respectively). In a meta-analysis of 70 studies, social isolation, loneliness and living alone were associated with a 26–32% increased mortality risk [16]. There was no evidence of differences in mortality between these measures, although the strength of association was greater in individuals below the age of 65, consistent with our findings. A recent UK Biobank analysis found that socially isolated and lonely individuals had an increased risk of death, but only social isolation predicted all-cause mortality in a joint model [22]. The discrepancy with our finding (loneliness was more strongly associated with poor health) might reflect differences in outcome measures (general health in the present study vs mortality in previous investigations).

Long sleep duration, high BMI and past and current smoking were associated with poor health. Sleeping less than 7 h/day was also associated with poor health, consistent with a meta-analysis that provided evidence of a U-shaped association between sleep duration and all-cause mortality [36]. A BMI outside the optimal range of 18.5–24.9 kg/m^2^ was also associated with poor health, consistent with previous research that examined all-cause mortality [60].

Physical activity is a key lifestyle factor recommended for primary and secondary prevention of chronic health conditions [63] and is associated with lower mortality risk [64]. In this study, walking frequency, especially in individuals aged 65 and above, stair climbing, and engaging in vigorous physical activity was associated with good health. Moderate physical activity was associated with better self-rated health, especially in men. A study in middle-aged British men found evidence of an association between vigorous, but not moderate, physical activity and reduced mortality [65]. Reviews of the literature report mixed findings on the relative contributions of moderate and vigorous physical activity [66, 67], with some evidence suggesting stronger associations for vigorous activity [68].

A more frequent drinking pattern was associated with better health in this study. Alcohol drinking often occurs in a social context and might therefore constitute a proxy for social wellness, supported by the finding that non-drinkers tend to be characterised by poor psychosocial health and low socioeconomic status [69]. Moderate drinkers also perform more sports than lifelong abstainers [70]. Drinking less frequent than 1–2 times/week was associated with poor health and could not be fully accounted for by excluding individuals who had discontinued alcohol intake for health reasons or because of their doctor’s advice. However, the association with health status was stronger for current abstainers, suggesting that some individuals are non-drinkers in later life due to illness [71]. Current abstainers who quit drinking for health reasons could exaggerate poor health outcomes associated with not drinking alcohol [72]. Those who never drink might also differ from current drinkers in other characteristics [69].

Road traffic noise and ambient air pollution are environmental risk factors for health. While we found some evidence that higher levels of all airborne pollutants were associated with poor health, only PM_2.5_ was associated with unfavourable health status and having a long-standing illness after adjustment for other factors. Higher levels of NO_2_ were associated with less favourable self-rated health. A joint analysis of UK Biobank, HUNT, and EPIC-Oxford data did not find evidence of an association between NO_2_ and incident cardiovascular disease, although both PM_2.5_ and PM_10_ were associated with higher incident cardiovascular disease [73]. Adjustment for physical activity and neighbourhood deprivation in the present study could have contributed to differences in findings between studies. Few individuals in our study were exposed to levels of PM_10_ and NO_2_ above recommended guidelines, which might explain why we did not find strong associations between these pollutants and health. The only exception was PM_2.5_, for which 45% of our sample were exposed to levels above the WHO recommendation threshold of ≤ 10 μg/m^3^. Positive associations between PM_2.5_ and self-rated health might represent spurious associations or statistical artefacts; we are not aware of any mechanisms linking higher levels of pollution to good health. While higher levels of residential noise were associated with poor health in Models 1 and 2, there was no evidence of an association after adjustment for other factors. Our findings are consistent with a previous analysis that did not find evidence of an independent association between L_den_ and cardiovascular disease, ischemic heart disease or cerebrovascular disease [73].

We found some evidence that greenspace was associated with better self-rated health, consistent with a recent meta-analysis [74], although not after adjustment for other factors. Several previous studies examining associations between greenspace and health did not adjust for physical activity and most did not include other environmental exposures such as air pollution or noise [74]. These differences could partially explain why we did not find consistent associations in this study.

### Strengths and limitations

The large sample size enabled high precision in the estimation of associations, and it allowed us to explore a wide range of explanatory variables, sex and age-specific associations, interactions and additional analyses with further classification of explanatory variables. Many of the findings from previous studies that we replicated in our analyses were conducted in smaller populations and with one exposure at a time, often providing limited insight into the multifactorial nature of health. One of the contributions of this study is that it allows for systematic comparisons of a broad range of factors associated with health. We examined three health indicators with slightly different ascertainment. While our findings were fairly consistent across these outcomes, we also identified differences between objective and subjective health. Overall health is arguably what matters most to people, and exploring factors associated with health indicators that transcend traditional disease-boundaries could represent an effective strategy to increase longevity and improve healthy life expectancy. Previous studies have often used composite scores of healthy lifestyles [14] in which individual behaviours were weighted equally, even though the strength of their respective associations with health might differ. In the present study, we estimated associations separately for each lifestyle factor.

Nevertheless, our study has limitations. Cross-sectional analyses prevent examination of temporal sequences. Reverse causality such as poor health and disability leading to an unhealthy lifestyle, less favourable socioeconomic outcomes, fewer social interactions or living in more deprived and polluted areas needs to be considered, except for fixed characteristics such as sex and ethnicity. Associations reported here cannot be interpreted as causal effects and there are model-specific limitations that need to be considered when interpreting these results. Residual confounding needs to be considered when examining associations from Model 1 and Model 2, while associations from Model 4 might be over-adjusted and there is the possibility that some variables included in Model 4 could lie on the causal pathway of other variables. The intermediate Model 3 in which we examined only variables from each broad group of explanatory variables plus age and sex partially addresses this concern and provided mostly consistent results. Although we have focussed on associations that were consistent across Models 1–4 in the main body of the text and highlighted inconsistencies, there was a large degree of consistency in direction of association across models and health indicators. As noted above, the notable exception to this pattern was education, for which we did not find consistent associations with favourable health in the full multivariable model. This is likely due to other explanatory variables (e.g. lifestyle) being on the causal pathway from education, which is determined fairly early in life, to health in middle to old age. Indeed, a potential limitation of using a standard set of covariates across models is that this approach does not fully consider the specific role each covariate may have for a given exposure-outcome pair.

As many explanatory variables were self-reported, we cannot exclude the possibility that social desirability might have influenced responses. For example, previous research has shown that up to half of participants who claim to never have had an alcoholic drink reported alcohol intake during previous surveys [75]. For most participants, explanatory variables were measured at a single time point, which could increase the potential for measurement error and did not allow us to model changes across the life course. However, there is evidence that lifestyle is fairly stable in this age group, with few people newly adopting a healthy lifestyle in middle age [76]. Future research should explore predictors of changes in health status over time using longitudinal data, for example using linked patient records. We did not include a measure of diet quality or quantity in our analyses, despite it being a key health-related behaviour, as detailed information on diet at the baseline assessment was only available for about 13% of participants (prior to applying exclusion criteria) [77]; examining the frequency of specific food consumption available for most of the UK Biobank sample was beyond the scope of this study. There might be some misclassification in the reporting of medical illnesses. However, participants were asked to report illnesses that had been diagnosed by a doctor and these were confirmed during the nurse-led interview.

### Generalisability

The overall response rate of the UK Biobank was low (5.5%) and compared with non-responders, participants were older, more likely to be female and lived in less deprived neighbourhoods. Participants were also less likely to be obese, to smoke, to never drink or to drink alcohol daily/almost daily and they reported fewer health conditions compared with data from a nationally representative survey [78]. While the UK Biobank states that “valid assessment of exposure-disease relationships are nonetheless widely generalizable and do not require participants to be representative of the population at large” [79], concordant with the finding that there is little evidence of considerable bias due to non-participation in epidemiological research [80], the magnitude of exposure-disease associations may depend on the prevalence of effect modifiers [81]. A recent empirical investigation comparing the UK Biobank with data from 18 prospective cohort studies with conventional response rates showed that the direction of risk factor associations were similar, although with differences in magnitude [82].

## Conclusion

Our findings shed light on some of the behavioural, psychosocial and environmental pathways to health and are thus relevant to public health policies aimed at promoting health in later life. Public health and medicine could put greater focus on non-medical factors such as loneliness, further encourage healthy lifestyle behaviours and weight management and examine efforts to improve the health outcomes of individuals on a low income. Our findings support the view that promoting individual responsibility for one’s health (e.g. engaging in healthy lifestyle behaviours) and policy-level commitments (e.g. reducing long-term exposure to environmental pollutants [83] or improving neighbourhoods) both need to be considered in population health. While associations between lifestyle and health might reflect a lifelong commitment to healthy behaviours, it is not too late to newly adopt healthy behaviours later in life [76]. Prevention, in addition to drug discovery and disease treatment, should be the top priority for health policy.

## Supplementary Information

**Additional file 1: Table S1.** Data fields. **Figure S1.** Study flowchart. **Table S2.** Baseline characteristics. **Figures S2-S3.** Self-reported illnesses. **Figure S4.** Correlation matrix of continuous explanatory variables. **Tables S3-S5.** Descriptive statistics stratified by health indicators. **Tables S6-S9.** Visual summary of findings. **Tables S10-S13.** Regression tables long-standing illness. **Tables S14-S17.** Regression tables self-rated health. **Tables S18-S21.** Regression tables health indicators. **Tables S22-S24.** Standardised regression coefficients tables. **Figures S5-S6.** Standardised regression coefficients plots. **Table S25.** Baseline characteristics stratified by sex. **Table S26.** Baseline characteristics stratified by age. **Table S27-S30.** Regression tables health status stratified by sex. **Figures S7-S9.** Confidence interval plots health status stratified by sex. **Tables S31-S34.** Regression tables health status stratified by age. **Figures S10-S12.** Confidence interval plots health status stratified by age. **Tables S35-S38.** Regression tables long-standing illness stratified by sex. **Figures S13-S15.** Confidence interval plots long-standing illness stratified by sex. **Tables S39-S42.** Regression tables long-standing illness stratified by age. **Figures S16-S18.** Confidence interval plots long-standing illness stratified by age. **Tables S43-S46.** Regression tables self-rated health stratified by sex. **Figures S19-S21.** Confidence interval plots self-rated health stratified by sex. **Tables S47-S50.** Regression tables self-rated health stratified by age. **Figures S22-S24.** Confidence interval plots self-rated health stratified by age. **Table S51.** Baseline characteristics longitudinal samples. **Tables S52-S55.** Regression tables self-rated health t1. **Tables S56-S59.** Regression tables self-rated health t2. **Table S60.** Descriptive statistics additional analyses. **Figure S25.** Generalised additive models.

**Additional file 2.** Health status classification.

## Data Availability

The data used in the present study are available to all bona fide researchers for health-related research that is in the public interest, subject to an application process and approval criteria. Study materials are publicly available online at http://www.ukbiobank.ac.uk.
