# Supplementary material for: Exploring health in the UK Biobank: associations with sociodemographic characteristics, psychosocial factors, lifestyle and environmental exposures
Source: BMC Med. 2021 Oct 11;19:240. doi: 10.1186/s12916-021-02097-z (PMC8504077; doi:10.1186/s12916-021-02097-z)
Supplement: Supplementary file 1 — Additional file 1: Table S1. Data fields. Figure S1. Study flowchart. Table S2. Baseline characteristics. Figures S2-S3. Self-reported illnesses. Figure S4. Correlation matrix of continuous explanatory variables. Tables S3-S5. Descriptive statistics stratified by health indicators. Tables S6-S9. Visual summary of findings. Tables S10-S13. Regression tables long-standing illness. Tables S14-S17. Regression tables self-rated health. Tables S18-S21. Regression tables health indicators. Tables S22-S24. Standardised regression coefficients tables. Figures S5-S6. Standardised regression coefficients plots. Table S25. Baseline characteristics stratified by sex. Table S26. Baseline characteristics stratified by age. Table S27-S30. Regression tables health status stratified by sex. Figures S7-S9. Confidence interval plots health status stratified by sex. Tables S31-S34. Regression tables health status stratified by age. Figures S10-S12. Confidence interval plots health status stratified by age. Tables S35-S38. Regression tables long-standing illness stratified by sex. Figures S13-S15. Confidence interval plots long-standing illness stratified by sex. Tables S39-S42. Regression tables long-standing illness stratified by age. Figures S16-S18. Confidence interval plots long-standing illness stratified by age. Tables S43-S46. Regression tables self-rated health stratified by sex. Figures S19-S21. Confidence interval plots self-rated health stratified by sex. Tables S47-S50. Regression tables self-rated health stratified by age. Figures S22-S24. Confidence interval plots self-rated health stratified by age. Table S51. Baseline characteristics longitudinal samples. Tables S52-S55. Regression tables self-rated health t1. Tables S56-S59. Regression tables self-rated health t2. Table S60. Descriptive statistics additional analyses. Figure S25. Generalised additive models. [file 12916_2021_2097_MOESM1_ESM.pdf]

Supplementary material

The following material accompanies the article

Exploring health in the UK Biobank: associations with sociodemographic characteristics, psychosocial factors, lifestyle and environmental exposures

by Julian Mutz, Charlotte J Roscoe and Cathryn M Lewis

published in BMC Medicine

Table of contents

TABLE S1. DATA FIELDS **4**
FIGURE S1. STUDY FLOWCHART **5**
TABLE S2. BASELINE CHARACTERISTICS **6**
FIGURES S2-S3. SELF-REPORTED ILLNESSES **9**
Cancer illnesses 9
Non-cancer illnesses 10
FIGURE S4. CORRELATION MATRIX CONTINUOUS VARIABLES **11**
TABLES S3-S5. DESCRIPTIVE STATISTICS STRATIFIED BY HEALTH INDICATORS **12**
Stratified by health status 12
Stratified by long-standing illness 14
Baseline characteristics stratified by self-rated health 16
TABLES S6-S9. VISUAL SUMMARY OF FINDINGS **18**
Lifestyle factors 18
Environmental exposures 19
Psychosocial factors 20
Sociodemographic characteristics 21
TABLES S10-S13. REGRESSION TABLES LONG-STANDING ILLNESS **22**
Sociodemographic characteristics 22
Psychosocial factors 23
Lifestyle factors 24
Environmental exposures 25
TABLES S14-S17. REGRESSION TABLES SELF-RATED HEALTH **26**
Sociodemographic characteristics 26
Psychosocial factors 27
Lifestyle factors 28
Environmental exposures 29
TABLES S18-S21. REGRESSION TABLES HEALTH INDICATORS **30**
Sociodemographic characteristics 30
Psychosocial factors 31
Lifestyle factors 32
Environmental exposures 33
TABLES S22-S24. STANDARDISED REGRESSION COEFFICIENTS TABLES **34**
Health status 34
Long-standing illness 35
Self-rated health 36
FIGURES S5-S6. STANDARDISED REGRESSION COEFFICIENTS PLOTS **37**
Long-standing illness 37
Self-rated health 38
TABLE S25. BASELINE CHARACTERISTICS STRATIFIED BY SEX **39**
TABLE S26. BASELINE CHARACTERISTICS STRATIFIED BY AGE **41**
TABLE S27-S30. REGRESSION TABLES HEALTH STATUS STRATIFIED BY SEX **43**
Sociodemographic characteristics 43
Psychosocial factors 44
Lifestyle factors 45
Environmental exposures 46
FIGURES S7-S9. CONFIDENCE INTERVAL PLOTS HEALTH STATUS STRATIFIED BY SEX **47**
Sociodemographic and psychosocial factors 47
Lifestyle factors 48
Environmental exposures 49
TABLES S31-S34. REGRESSION TABLES HEALTH STATUS STRATIFIED BY AGE **50**
Sociodemographic characteristics 50
Psychosocial factors 51
Lifestyle factors 52
Environmental exposures 53
FIGURES S10-S12. CONFIDENCE INTERVAL PLOTS HEALTH STATUS STRATIFIED BY AGE **54**
Sociodemographic and psychosocial factors 54
Lifestyle factors 55
Environmental exposures 56
TABLES S35-S38. REGRESSION TABLES LONG-STANDING ILLNESS STRATIFIED BY SEX **57**
Sociodemographic characteristics 57
Psychosocial factors 58
Lifestyle factors 59
Environmental exposures 60
FIGURES S13-S15. CONFIDENCE INTERVAL PLOTS LONG-STANDING ILLNESS STRATIFIED BY SEX **61**
Sociodemographic and psychosocial factors 61
Lifestyle factors 62
Environmental exposures 63
TABLES S39-S42. REGRESSION TABLES LONG-STANDING ILLNESS STRATIFIED BY AGE **64**
Sociodemographic characteristics 64
Psychosocial factors 65
Lifestyle factors 66
Environmental exposures 67
FIGURES S16-S18. CONFIDENCE INTERVAL PLOTS LONG-STANDING ILLNESS STRATIFIED BY AGE **68**

Sociodemographic and psychosocial factors 68
Lifestyle factors 69
Environmental exposures 70
TABLES S43-S46. REGRESSION TABLES SELF-RATED HEALTH STRATIFIED BY SEX 71
Sociodemographic characteristics 71
Psychosocial factors 72
Lifestyle factors 73
Environmental exposures 74
FIGURES S19-S21. CONFIDENCE INTERVAL PLOTS SELF-RATED HEALTH STRATIFIED BY SEX 75
Sociodemographic and psychosocial factors 75
Lifestyle factors 76
Environmental exposures 77
TABLES S47-S50. REGRESSION TABLES SELF-RATED HEALTH STRATIFIED BY AGE 78
Sociodemographic characteristics 78
Psychosocial factors 79
Lifestyle factors 80
Environmental exposures 81
FIGURES S22-S24. CONFIDENCE INTERVAL PLOTS SELF-RATED HEALTH STRATIFIED BY AGE 82
Sociodemographic and psychosocial factors 82
Lifestyle factors 83
Environmental exposures 84
TABLE S51. BASELINE CHARACTERISTICS LONGITUDINAL SAMPLES 85
TABLES S52-S55. REGRESSION TABLES SELF-RATED HEALTH T1 87
Sociodemographic characteristics 87
Psychosocial factors 88
Lifestyle factors 89
Environmental exposures 90
TABLES S56-S59. REGRESSION TABLES SELF-RATED HEALTH T2 91
Sociodemographic characteristics 91
Psychosocial factors 92
Lifestyle factors 93
Environmental exposures 94
TABLE S60. DESCRIPTIVE STATISTICS ADDITIONAL ANALYSES 95
Descriptive statistics 95
FIGURE S25. GENERALISED ADDITIVE MODELS 96

Table S1. Data fields

Table S1. **Data fields used in the present study.**

**UK Biobank data field**	**Variable name**
**Sociodemographic characteristics**	
31	Sex
738	Average total household income before tax
6138[1]	Qualifications
21000[1]	Ethnic background
21003	Age when attended assessment centre
22001	Genetic sex
26410	Index of Multiple Deprivation (England)
**Psychosocial factors**	
709[2]	Number in household
1031[2]	Frequency of friend/family visits
6160[2]	Leisure/social activities
2020[3]	Loneliness, isolation
2110[3]	Able to confide
**Lifestyle factors**	
864	Number of days per week walked 10+ minutes
884	Number of days per week of moderate physical activity 10+ minutes
904	Number of days per week of vigorous physical activity 10+ minutes
943	Frequency of stair climbing in last 4 weeks
1160	Sleep duration
1239	Current tobacco smoking
1558	Alcohol intake frequency
3731	Former alcohol drinker
3859	Reason former drinker stopped drinking alcohol
20116	Smoking status
22037	MET minutes per week for walking
22038	MET minutes per week for moderate activity
22039	MET minutes per week for vigorous activity
23099	Body fat percentage
23104	Body mass index (BMI)
**Environmental exposures**	
24003	Nitrogen dioxide air pollution; 2010
24005	Particulate matter air pollution (pm10); 2010
24006	Particulate matter air pollution (pm2.5); 2010
24024	Average 24-hour sound level of noise pollution
24500	Greenspace percentage, buffer 1000m
24503	Greenspace percentage, buffer 300m
**Health indicators**	
2178	Overall health rating
2188	Long-standing illness, disability of infirmity
20001[4]	Cancer code, self-reported
20002[4]	Non-cancer illness code, self-reported
**Other**	
53	Date of attending assessment centre
699	Length of time at current address
3140	Pregnant

Note: [1] these variables have been further processed and categories used in the present study are described in the main body of the text. [2] variables used to derive social isolation index. [3] variables used to derive loneliness index. [4] variables used to derive health status.

Loneliness index:

1. If the response to one question was missing, “don’t know” or “prefer not to answer” and the response to the second question was scored 0, individuals were classified as not lonely.
2. If the response to one question was missing, “don’t know” or “prefer not to answer” and the response to the second question was scored 1, individuals were classified as missing data on loneliness.
3. If responses to both questions were missing, “don’t know” or “prefer not to answer”, individuals were classified as missing data on loneliness.

Social isolation index:

1. If the response to two questions were scored 1 and the response to the third question was missing, “don’t know” or “prefer not to answer”, individuals were classified as socially isolated.
2. If two or more responses to questions were missing, “don’t know” or “prefer not to answer”, individuals were classified as missing data on social isolation.
3. If the response to two questions were scored 0 and the response to the third question was missing, “don’t know” or “prefer not to answer”, individuals were classified as not socially isolated.
4. If the response to one question was scored 0, the response to one question was scored 1 and the response to the third question was missing, “don’t know” or “prefer not to answer”, individuals were classified as missing data on social isolation.

Figure S1. Study flowchart

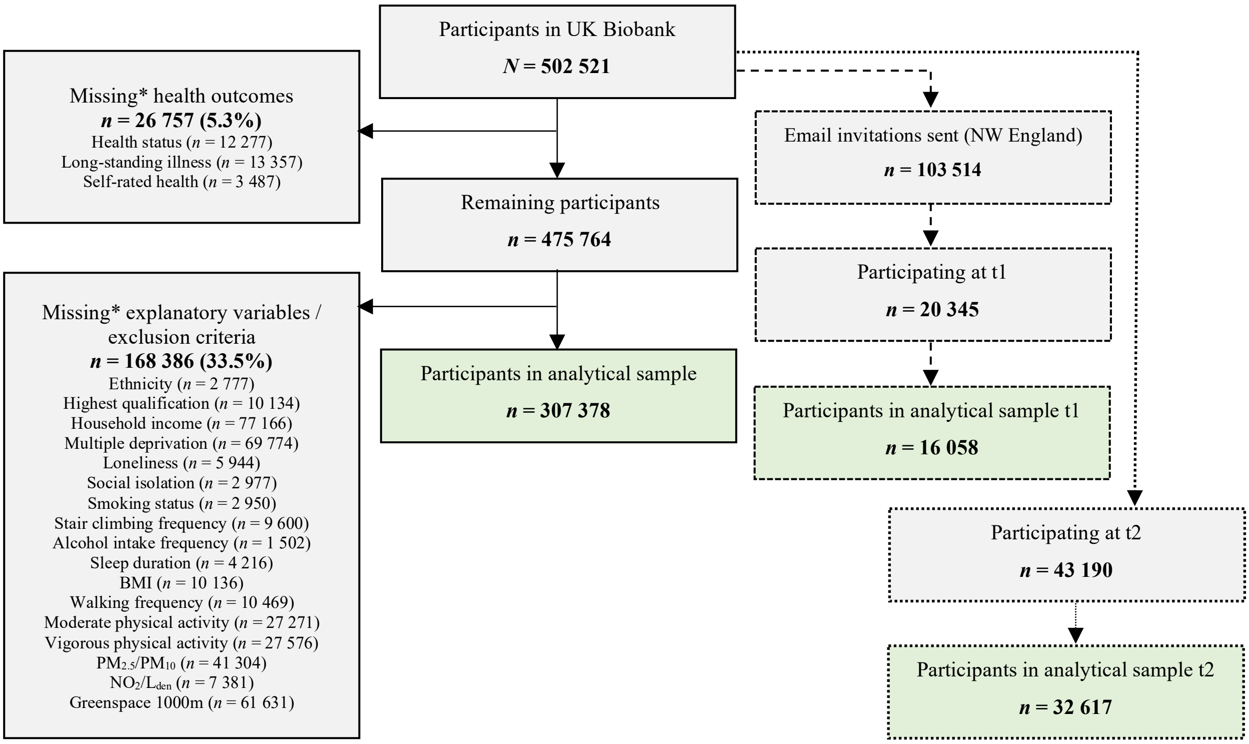

Figure S1. Flowchart of study population. t1 = follow-up during first revisit between 2012–2013; t2 = follow-up during UK Biobank Imaging Study between 2014-2019. BMI = body mass index; PM = particulate matter; NO_2 = nitrogen dioxide; L_{den} = day-evening-night noise level. *Numbers for missing data for individual variables are not mutually exclusive and include "prefer not to answer" and "do not know".

Table S2. Baseline characteristics

Table S2. **Baseline characteristics**

	Full sample (N = 502 521)	Analytical sample (n = 307 378)
Health indicators		
**Health status**		
Unhealthy	162 096 (32.3%)	95 177 (31.0%)
Healthy	328 148 (65.3%)	212 201 (69.0%)
Missing	12 277 (2.4%)	
**Long-standing illness**		
Yes	159 904 (31.8%)	93 757 (30.5%)
No	329 260 (65.5%)	213 621 (69.5%)
Prefer not to answer	1 052 (0.2%)	
Do not know	11 387 (2.3%)	
Missing	918 (0.2%)	
**Self-rated health**		
Poor	22 778 (4.5%)	11 066 (3.6%)
Fair	105 373 (21.0%)	59 169 (19.2%)
Good	289 023 (57.5%)	182 699 (59.4%)
Excellent	81 860 (16.3%)	54 444 (17.7%)
Prefer not to answer	365 (0.1%)	
Do not know	2 204 (0.4%)	
Missing	918 (0.2%)	
Sociodemographic characteristics		
**Age**		
Mean (SD)	56.53 (8.10)	56.11 (8.01)
Range	37-73	38-73
**Sex**		
Female	273 394 (54.4%)	159 574 (51.9%)
Male	229 127 (45.6%)	147 804 (48.1%)
**Ethnicity**		
White	472 711 (94.1%)	293 565 (95.5%)
Mixed-race	2 958 (0.6%)	1 766 (0.6%)
Black	8 061 (1.6%)	4 257 (1.4%)
Asian	9 882 (2.0%)	4 755 (1.5%)
Chinese	1 574 (0.3%)	818 (0.3%)
Other	4 558 (0.9%)	2 217 (0.7%)
Prefer not to answer	1 662 (0.3%)	
Do not know	217 (0.0%)	
Missing	898 (0.2%)	
**Highest qualification**		
None	85 274 (17.0%)	39 828 (13.0%)
O levels/GCSEs/CSEs	132 086 (26.3%)	84 448 (27.5%)
A levels/NVQ/HND/HNC[1]	113 859 (22.7%)	72 584 (23.6%)
Degree	161 168 (32.1%)	110 518 (36.0%)
Prefer not to answer	5 493 (1.1%)	
Missing	4 641 (0.9%)	
**Household income**[2]		
Very low	97 205 (19.3%)	63 099 (20.5%)
Low	108 177 (21.5%)	77 931 (25.4%)
Medium	110 774 (22.0%)	82 338 (26.8%)
High	86 269 (17.2%)	66 106 (21.5%)
Very high	22 930 (4.6%)	17 904 (5.8%)
Prefer not to answer	49 848 (9.9%)	
Do not know	21 305 (4.2%)	
Missing	6 013 (1.2%)	
**Multiple deprivation**		
Mean (SD)	17.68 (14.01)	16.77 (13.33)
Range	0.61-82	0.61-82
Missing	69 774 (13.1%)	
Psychosocial factors		
**Loneliness**		
Not lonely	466 182 (92.8%)	289 901 (94.3%)
Lonely	30 395 (6.0%)	17 477 (5.7%)
Missing	5 944 (1.2%)	
**Social isolation**		
Not isolated	453 401 (90.2%)	280 931 (91.4%)
Isolated	46 143 (9.2%)	26 447 (8.6%)
Missing	2 977 (0.6%)	
Lifestyle factors		
**Smoking status**		
Never	273 528 (54.4%)	168 475 (54.8%)
Former	173 064 (34.4%)	108 638 (35.3%)
Current	52 979 (10.5%)	30 265 (9.8%)

Prefer not to answer	2 059 (0.4%)	
Missing	891 (0.2%)	
**Stair climbing frequency**		
None	44 988 (9.0%)	24 049 (7.8%)
1-5/day	100 569 (20.0%)	58 267 (19.0%)
6-10/day	178 969 (35.6%)	115 982 (37.7%)
11-15/day	91 352 (18.2%)	60 315 (19.6%)
16-20/day	42 477 (8.5%)	27 609 (9.0%)
20+/day	34 566 (6.9%)	21 156 (6.9%)
Prefer not to answer	461 (0.1%)	
Do not know	2 580 (0.5%)	
Missing	6 559 (1.3%)	
**Alcohol intake frequency**		
Never	40 645 (8.1%)	20 423 (6.6%)
Special occasions	58 011 (11.5%)	31 526 (10.3%)
1-3/month	55 856 (11.1%)	33 798 (11.0%)
1-2/week	129 294 (25.7%)	78 777 (25.6%)
3-4/week	115443 (23.0%)	75 251 (24.5%)
Daily/almost daily	10 770 (20.3%)	67 603 (22.0%)
Prefer not to answer	605 (0.1%)	
Missing	897 (0.2%)	
**Sleep duration** (hours/day)		
Mean (SD)	7.15 (1.11)*	7.16 (1.06)
Range	1-23*	1-20
Prefer not to answer	386 (0.1%)	
Do not know	2943 (0.6%)	
Missing	887 (0.2%)	
**BMI** (kg/m^2)		
Mean (SD)	27.43 (4.79)	27.27 (4.67)
Range	12.80-68.40	12.80-67.30
Missing	10 136 (2.0%)	
**Walking** (days/week)[3]		
Mean (SD)	5.39 (1. 93)§	5.36 (1.95)
Range	0-7§	0-7
Prefer not to answer	979 (0.2%)	
Do not know	6 687 (1.3%)	
Unable to walk	1 929 (0.4%)	
Missing	874 (0.2%)	
**Moderate activity** (days/week)[3]		
Mean (SD)	3.63 (2.33)±	3.59 (2.32)
Range	0-7±	0-7
Prefer not to answer	2 273 (0.5%)	
Do not know	24 120 (4.8%)	
Missing	878 (0.2%)	
**Vigorous activity** (days/week)[3]		
Mean (SD)	1.84 (1.96)$	1.876 (1.94)
Range	0-7$	0-7
Prefer not to answer	4 116 (0.8%)	
Do not know	22 582 (4.5%)	
Missing	878 (0.2%)	
Environmental exposures		
**$PM_{2.5}$**		
Mean (SD)	9.99 (1.06)	9.95 (1.04)
Range	8.17-21.31	8.17-21.25
Missing	41 304 (8.2%)	
**PM_{10}**		
Mean (SD)	16.24 (1. 90)	16.19 (1.88)
Range	11.78-31.39	11.78-30.65
Missing	41 304 (8.2%)	
**NO_2**		
Mean (SD)	26.71 (7.58)	26.44 (7.56)
Range	12.93-108.49	12.93-108.49
Missing	7 381 (1.5%)	
**L_{den}**		
Mean (SD)	56.06 (4.28)	56.01 (4.24)
Range	51.54-93.36	51.55-89.29
Missing	7 381 (1.5%)	
**Greenspace** 1000m		
Mean (SD)	44.99 (21.62)	45.52 (21.77)
Range	4.42-99.19	4.49-99.19
Missing	61 631 (12.3%)	

Note: GCSEs = general certificate of secondary education; CSE = certificate of secondary education; NVQ = national vocational qualification; HND = higher national diploma; HNC = higher national certificate; BMI = body mass index; PM = particulate matter; NO_2 = nitrogen dioxide; L_{den} = day-evening-night noise level. [1]also includes 'other professional qualifications'. [2]Annual household income groups: very low (<£18,000), low

(£18,000–£30,999), middle (£31,000–£51,999), high (£52,000–£100,000) and very high (>£100,000). [3]number of days per week engaging in these activities for 10+ minutes continuously. [*]n=498 305; [§]n=492 052; [±]n=475 250; [$]$n$=474 945.

Additional information

Descriptive statistic in manuscript on duration living at current address does not include participants who lived at their current address for <1 year (n = 4,781), but these individuals were included in all other analyses. The average number of days between the baseline assessment and the air pollution exposure estimate (calculated based on the midpoint between 26 January 2010 and 18 January 2011) was 491 days (SD = 285; range = –56 to 1312). The corresponding figures for traffic noise were 105 days (SD = 285; range = –442 to 926).

Figures S2-S3. Self-reported illnesses

Cancer illnesses

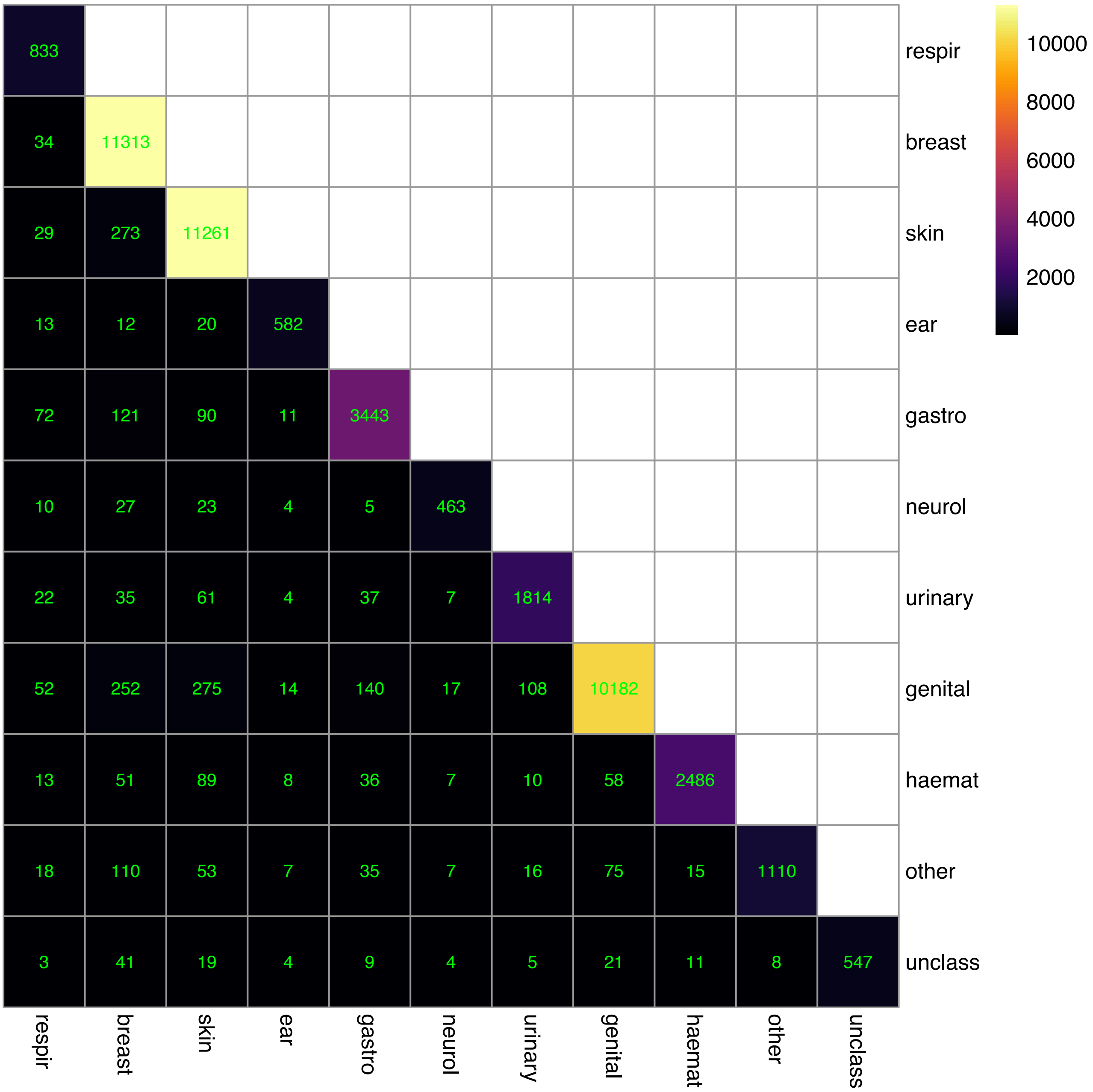

Figure S2. Self-reported cancer illnesses. Cell entries reflect the number of individuals with ≥1 cancer illness of the group in the corresponding row and ≥1 cancer illness of the group in the corresponding column (e.g. 273 participants report ≥1 breast cancer and ≥1 skin cancer). Cell entries in the diagonal reflect the number of participants with ≥1 cancer illness of the group in the corresponding row or column (e.g. 11 313 participants report ≥1 breast cancer). breast = breast cancer; skin = skin cancer; genital = genital tract cancer; gastro = gastrointestinal cancer; haemat = haematological malignancy; urinary = urinary tract cancer; other = other cancer; respir = respiratory / intrathoracic cancer; ear = ear/nose/throat cancer; neurol = neurological system cancer; unclass = unclassifiable.

Non-cancer illnesses

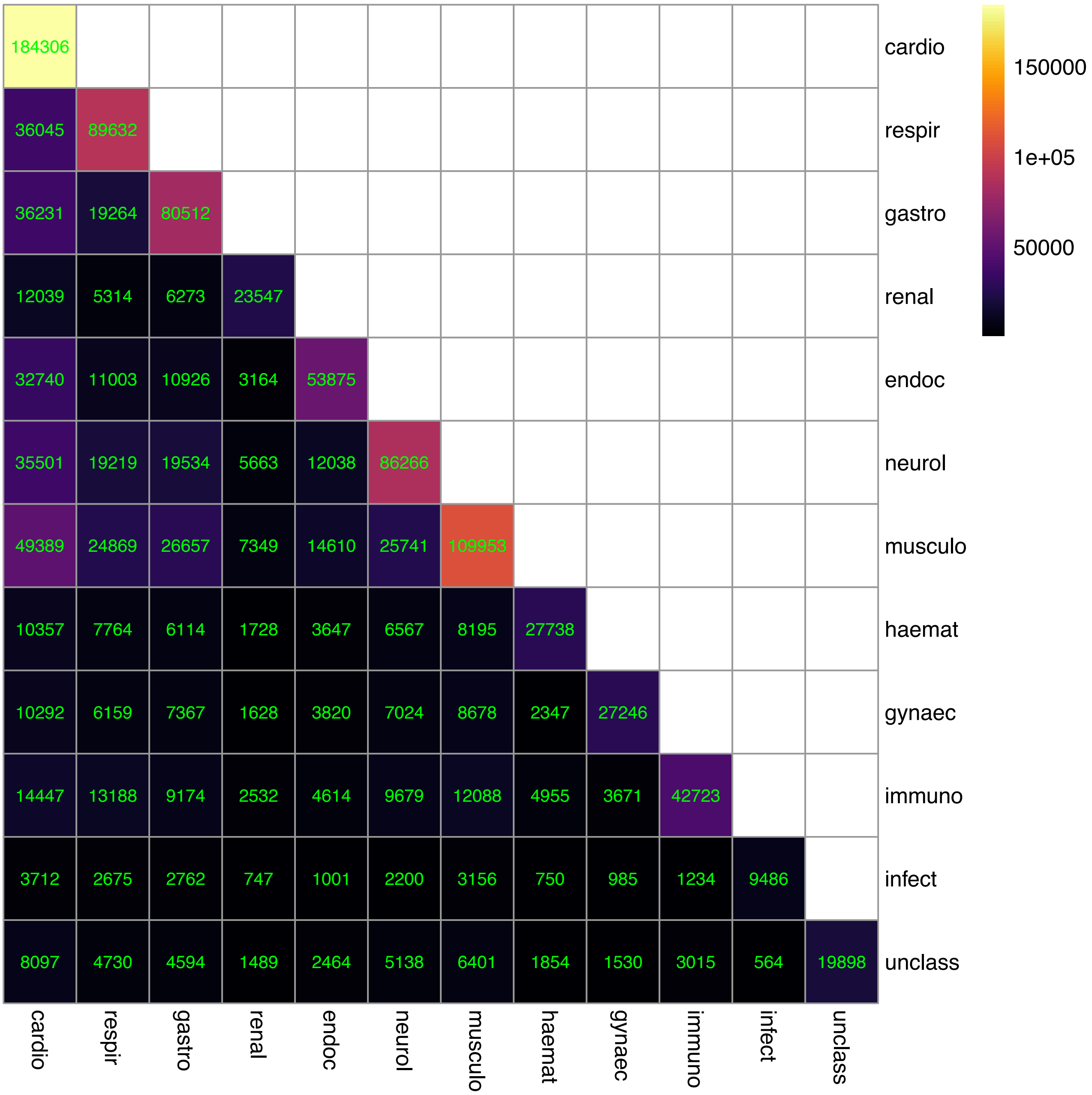

Figure S3. Self-reported non-cancer illnesses. Cell entries reflect the number of individuals with ≥1 non-cancer illness of the group in the corresponding row and ≥1 non-cancer illness of the group in the corresponding column (e.g. 3 712 participants report ≥1 cardiovascular illness and ≥1 infection). Cell entries in the diagonal reflect the number of participants with ≥1 non-cancer illness of the group in the corresponding row or column (e.g. 184 306 participants report ≥1 cardiovascular illness). cardio = cardiovascular; musculo = musculoskeletal/trauma; endoc = endocrine/diabetes; immuno = immunological/systemic disorders; haemat = haematology/dermatology; gynaec = gynaecology/breast; renal = renal/urology; infect = infections; unclass = unclassifiable; respir = respiratory/ent; gastro = gastrointestinal/abdominal; neurol = neurology/eye/psychiatry.

Figure S4. Correlation matrix continuous variables

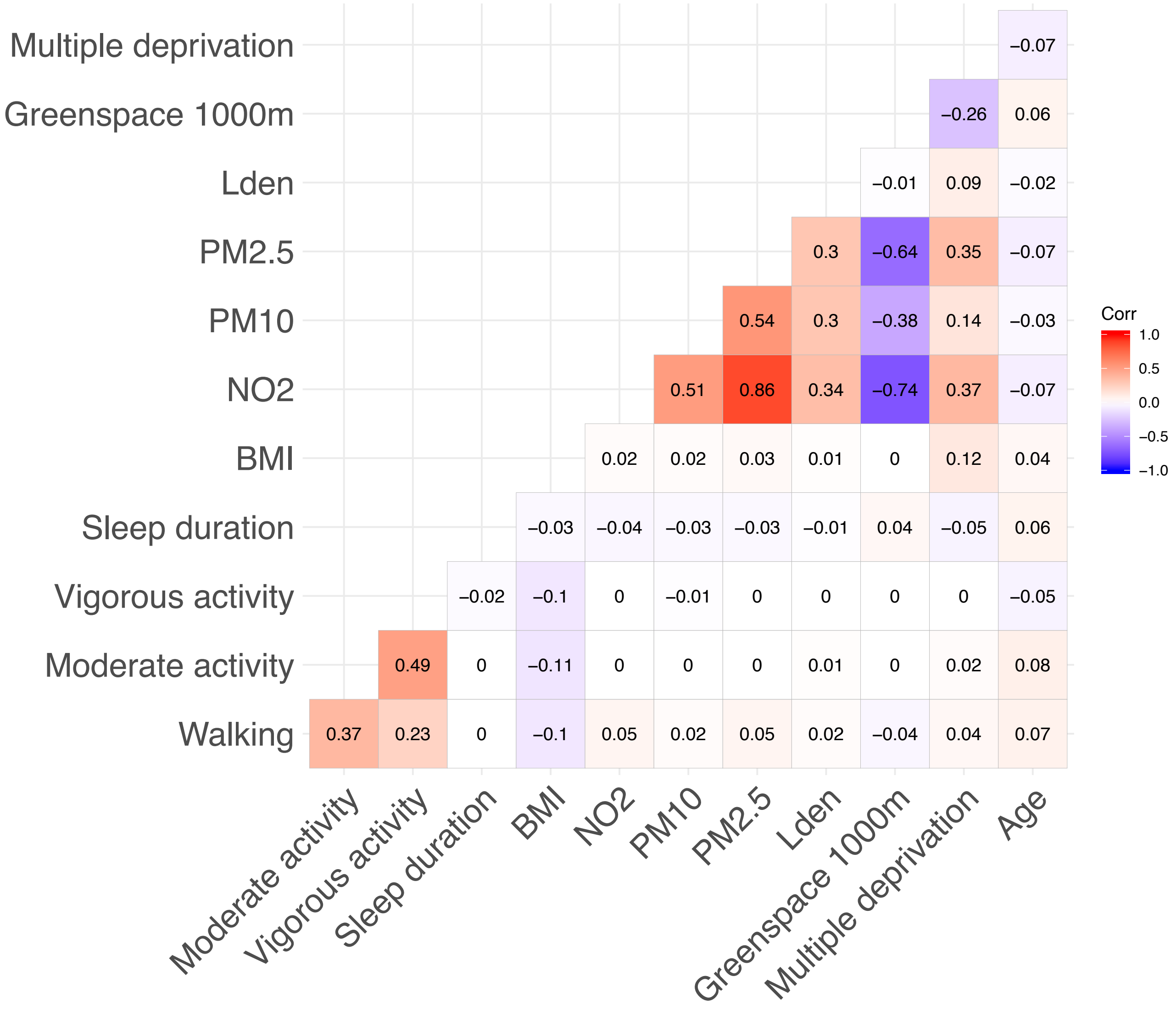

Figure S4. Correlation matrix of continuous explanatory variables.

Tables S3-S5. Descriptive statistics stratified by health indicators

Stratified by health status

Table S3. **Baseline characteristics stratified by health status**

	Unhealthy (*n* = 95 177)	Healthy (*n* = 212 201)	p value
Sociodemographic characteristics			
**Age**			< 0.001
Mean (SD)	58.46 (7.57)	55.05 (8.06)	
Range	38.00-72.00	39.00-73.00	
**Sex**			< 0.001
Female	47 016 (49.4%)	112 558 (53.0%)	
Male	48 161 (50.6%)	99 643 (47.0%)	
**Ethnicity**			< 0.001
White	91 415 (96.0%)	202 150 (95.3%)	
Mixed-race	456 (0.5%)	1 310 (0.6%)	
Black	1 099 (1.2%)	3 158 (1.5%)	
Asian	1 461 (1.5%)	3 294 (1.6%)	
Chinese	142 (0.1%)	676 (0.3%)	
Other	604 (0.6%)	1 613 (0.8%)	
**Highest qualification**			< 0.001
None	16 351 (17.2%)	23 477 (11.1%)	
O levels/GCSEs/CSEs	25 045 (26.3%)	59 403 (28.0%)	
A levels/NVQ/HND/HNC[1]	22 885 (24.0%)	49 699 (23.4%)	
Degree	30 896 (32.5%)	79 622 (37.5%)	
**Household income**[2]			< 0.001
Very low	26 699 (28.1%)	36 400 (17.2%)	
Low	26 029 (27.3%)	51 902 (24.5%)	
Medium	22 610 (23.8%)	59 728 (28.1%)	
High	15 769 (16.6%)	50 337 (23.7%)	
Very high	4 070 (4.3%)	13 834 (6.5%)	
**Multiple deprivation**			< 0.001
Mean (SD)	17.89 (14.15)	16.27 (12.92)	
Range	0.61-82.00	0.61-82.00	
Psychosocial factors			
**Loneliness**			< 0.001
Not lonely	88 594 (93.1%)	201 307 (94.9%)	
Lonely	6 583 (6.9%)	10 894 (5.1%)	
**Social isolation**			< 0.001
Not isolated	85 472 (89.8%)	195 459 (92.1%)	
Isolated	9 705 (10.2%)	16 742 (7.9%)	
Lifestyle factors			
**Smoking status**			< 0.001
Never	46 457 (48.8%)	122 018 (57.5%)	
Former	38 354 (40.3%)	70 284 (33.1%)	
Current	10 366 (10.9%)	19 899 (9.4%)	
**Stair climbing frequency**			< 0.001
None	9 590 (10.1%)	14 459 (6.8%)	
1-5/day	19 676 (20.7%)	38 591 (18.2%)	
6-10/day	34 711 (36.5%)	81 271 (38.3%)	
11-15/day	17 466 (18.4%)	42 849 (20.2%)	
16-20/day	7 918 (8.3%)	19 691 (9.3%)	
20+/day	5 816 (6.1%)	15 340 (7.2%)	
**Alcohol intake frequency**			< 0.001
Never	8 582 (9.0%)	11 841 (5.6%)	
Special occasions	11 621 (12.2%)	19 905 (9.4%)	
1-3/month	10 505 (11.0%)	23 293 (11.0%)	
1-2/week	22 890 (24.0%)	55 887 (26.3%)	
3-4/week	20 899 (22.0%)	54 352 (25.6%)	
Daily/almost daily	20 680 (21.7%)	46 923 (22.1%)	
**Sleep duration** (hours/day)			< 0.001
Mean (SD)	7.19 (1.19)	7.14 (0.99)	
Range	1.00-20.00	1.00-20.00	
**BMI** (kg/m^2)			< 0.001
Mean (SD)	27.96 (5.06)	26.96 (4.44)	
Range	12.80-66.20	12.80-67.30	
**Walking** (days/week)[3]			< 0.001
Mean (SD)	5.34 (1.98)	5.37 (1.93)	
Range	0.00-7.00	0.00-7.00	
**Moderate activity** (days/week)[3]			< 0.001
Mean (SD)	3.55 (2.37)	3.60 (2.30)	
Range	0.00-7.00	0.00-7.00	
**Vigorous activity** (days/week)[3]			< 0.001
Mean (SD)	1.73 (1.96)	1.94 (1.93)	

Range	0.00-7.00	0.00-7.00	
Environmental exposures			
**$PM_{2.5}$**			< 0.001
Mean (SD)	9.98 (1.05)	9.94 (1.03)	
Range	8.17-21.25	8.17-19.89	
**PM_{10}**			< 0.001
Mean (SD)	16.21 (1.88)	16.18 (1.88)	
Range	11.78-30.65	11.78-30.65	
**NO_2**			< 0.001
Mean (SD)	26.55 (7.60)	26.38 (7.54)	
Range	12.93-107.07	12.93-108.49	
**L_{den}**			< 0.001
Mean (SD)	56.05 (4.29)	55.99 (4.22)	
Range	51.55-86.50	51.55-89.29	
**Greenspace** 1000m			0.750
Mean (SD)	45.50 (21.57)	45.53 (21.86)	
Range	4.67-99.18	4.49-99.19	

Note: GCSEs = general certificate of secondary education; CSE = certificate of secondary education; NVQ = national vocational qualification; HND = higher national diploma; HNC = higher national certificate; BMI = body mass index; PM = particulate matter; NO_2 = nitrogen dioxide; L_{den} = day-evening-night noise level. [1]also includes 'other professional qualifications'. [2]Annual household income groups: very low (<£18,000), low (£18,000–£30,999), middle (£31,000–£51,999), high (£52,000–£100,000) and very high (>£100,000). [3]number of days per week engaging in these activities for 10+ minutes continuously.

Stratified by long-standing illness

Table S4. **Baseline characteristics stratified by long-standing illness**

	Yes<br>(*n* = 93 757)	No<br>(*n* = 213 621)	p value
Sociodemographic characteristics			
**Age**			< 0.001
Mean (SD)	57.70 (7.67)	55.41 (8.14)	
Range	39.00-72.00	38.00-73.00	
**Sex**			< 0.001
Female	44 607 (47.6%)	114 967 (53.8%)	
Male	49 150 (52.4%)	98 654 (46.2%)	
**Ethnicity**			< 0.001
White	89 561 (95.5%)	204 004 (95.5%)	
Mixed-race	487 (0.5%)	1 279 (0.6%)	
Black	1 419 (1.5%)	2 838 (1.3%)	
Asian	1 429 (1.5%)	3 326 (1.6%)	
Chinese	163 (0.2%)	655 (0.3%)	
Other	698 (0.7%)	1 519 (0.7%)	
**Highest qualification**			< 0.001
None	15 643 (16.7%)	24 185 (11.3%)	
O levels/GCSEs/CSEs	24 847 (26.5%)	59 601 (27.9%)	
A levels/NVQ/HND/HNC[1]	23 010 (24.5%)	49 574 (23.2%)	
Degree	30 257 (32.3%)	80 261 (37.6%)	
**Household income**[2]			< 0.001
Very low	27 247 (29.1%)	35 852 (16.8%)	
Low	25 264 (26.9%)	52 667 (24.7%)	
Medium	22 387 (23.9%)	59 951 (28.1%)	
High	15 291 (16.3%)	50 815 (23.8%)	
Very high	3 568 (3.8%)	14 336 (6.7%)	
**Multiple deprivation**			< 0.001
Mean (SD)	18.79 (14.62)	15.89 (12.63)	
Range	0.61-82.00	0.61-82.00	
Psychosocial factors			
**Loneliness**			< 0.001
Not lonely	86 392 (92.1%)	203 509 (95.3%)	
Lonely	7 365 (7.9%)	10 112 (4.7%)	
**Social isolation**			< 0.001
Not isolated	83 385 (88.9%)	197 546 (92.5%)	
Isolated	10 372 (11.1%)	16 075 (7.5%)	
Lifestyle factors			
**Smoking status**			< 0.001
Never	45 946 (49.0%)	122 529 (57.4%)	
Former	37 269 (39.8%)	71 369 (33.4%)	
Current	10 542 (11.2%)	19 723 (9.2%)	
**Stair climbing frequency**			< 0.001
None	9 858 (10.5%)	14 191 (6.6%)	
1-5/day	20 726 (22.1%)	37 541 (17.6%)	
6-10/day	33 819 (36.1%)	82 163 (38.5%)	
11-15/day	16 446 (17.5%)	43 869 (20.5%)	
16-20/day	7 373 (7.9%)	20 236 (9.5%)	
20+/day	5 535 (5.9%)	15 621 (7.3%)	
**Alcohol intake frequency**			< 0.001
Never	9 151 (9.8%)	11 272 (5.3%)	
Special occasions	12 274 (13.1%)	19 252 (9.0%)	
1-3/month	11 083 (11.8%)	22 715 (10.6%)	
1-2/week	22 588 (24.1%)	56 189 (26.3%)	
3-4/week	19 669 (21.0%)	55 582 (26.0%)	
Daily/almost daily	18 992 (20.3%)	48 611 (22.8%)	
**Sleep duration** (hours/day)			0.671
Mean (SD)	7.16 (1.24)	7.16 (0.96)	
Range	1.00-20.00	1.00-20.00	
**BMI** (kg/m^2)			< 0.001
Mean (SD)	28.46 (5.23)	26.75 (4.29)	
Range	12.80-67.30	12.80-66.00	
**Walking** (days/week)[3]			< 0.001
Mean (SD)	5.26 (2.04)	5.41 (1.90)	
Range	0.00-7.00	0.00-7.00	
**Moderate activity** (days/week)[3]			< 0.001
Mean (SD)	3.47 (2.39)	3.64 (2.28)	
Range	0.00-7.00	0.00-7.00	
**Vigorous activity** (days/week)[3]			< 0.001
Mean (SD)	1.65 (1.95)	1.97 (1.93)	
Range	0.00-7.00	0.00-7.00	
Environmental exposures			

**$PM_{2.5}$**			< 0.001
Mean (SD)	10.01 (1.05)	9.93 (1.03)	
Range	8.17-21.25	8.17-19.89	
**PM_{10}**			< 0.001
Mean (SD)	16.24 (1.86)	16.17 (1.88)	
Range	11.78-30.65	11.78-30.65	
**NO_2**			< 0.001
Mean (SD)	26.77 (7.53)	26.29 (7.57)	
Range	12.93-108.49	12.93-107.81	
**L_{den}**			0.002
Mean (SD)	56.05 (4.28)	56.00 (4.22)	
Range	51.55-86.50	51.55-89.29	
**Greenspace** 1000m			< 0.001
Mean (SD)	44.97 (21.27)	45.76 (21.98)	
Range	4.54-99.19	4.49-99.18	

Note: GCSEs = general certificate of secondary education; CSE = certificate of secondary education; NVQ = national vocational qualification; HND = higher national diploma; HNC = higher national certificate; BMI = body mass index; PM = particulate matter; NO_2 = nitrogen dioxide; L_{den} = day-evening-night noise level. [1]also includes 'other professional qualifications'. [2]Annual household income groups: very low (<£18,000), low (£18,000–£30,999), middle (£31,000–£51,999), high (£52,000–£100,000) and very high (>£100,000). [3]number of days per week engaging in these activities for 10+ minutes continuously.

Baseline characteristics stratified by self-rated health

Table S5. **Baseline characteristics stratified by self-rated health**

	Poor (*n* = 11 066)	Fair (*n* = 59 169)	Good (*n* = 182 699)	Excellent (*n* = 54 444)	p value
Sociodemographic characteristics					
**Age**					< 0.001
Mean (SD)	55.91 (7.77)	56.19 (8.09)	56.25 (8.07)	55.58 (8.06)	
Range	40.00-72.00	39.00-73.00	38.00-72.00	39.00-71.00	
**Sex**					< 0.001
Female	4 991 (45.1%)	27 657 (46.7%)	97 197 (53.2%)	29 729 (54.6%)	
Male	6 075 (54.9%)	31 512 (53.3%)	85 502 (46.8%)	24 715 (45.4%)	
**Ethnicity**					< 0.001
White	10 299 (93.1%)	55 498 (93.8%)	175 085 (95.8%)	52 683 (96.8%)	
Mixed-race	82 (0.7%)	401 (0.7%)	999 (0.5%)	284 (0.5%)	
Black	236 (2.1%)	1 127 (1.9%)	2 359 (1.3%)	535 (1.0%)	
Asian	311 (2.8%)	1 391 (2.4%)	2 538 (1.4%)	515 (0.9%)	
Chinese	21 (0.2%)	184 (0.3%)	499 (0.3%)	114 (0.2%)	
Other	117 (1.1%)	568 (1.0%)	1 219 (0.7%)	313 (0.6%)	
**Highest qualification**					< 0.001
None	2 723 (24.6%)	10 888 (18.4%)	21 910 (12.0%)	4 307 (7.9%)	
O levels/GCSEs/CSEs	3 133 (28.3%)	17 564 (29.7%)	51 129 (28.0%)	12 622 (23.2%)	
A levels/NVQ/HND/HNC[1]	2 664 (24.1%)	14 193 (24.0%)	43 648 (23.9%)	12 079 (22.2%)	
Degree	2 546 (23.0%)	16 524 (27.9%)	66 012 (36.1%)	25 436 (46.7%)	
**Household income**[2]					< 0.001
Very low	5 224 (47.2%)	16 903 (28.6%)	33 777 (18.5%)	7 195 (13.2%)	
Low	2 673 (24.2%)	15 853 (26.8%)	47 574 (26.0%)	11 831 (21.7%)	
Medium	1 889 (17.1%)	14 817 (25.0%)	50 698 (27.7%)	14 934 (27.4%)	
High	1 072 (9.7%)	9 699 (16.4%)	40 407 (22.1%)	14 928 (27.4%)	
Very high	208 (1.9%)	1 897 (3.2%)	10 243 (5.6%)	5 556 (10.2%)	
**Multiple deprivation**					< 0.001
Mean (SD)	24.45 (16.97)	19.66 (14.84)	16.08 (12.72)	14.39 (11.60)	
Range	0.61-82.00	0.61-82.00	0.61-82.00	0.61-82.00	
Psychosocial factors					
**Loneliness**					< 0.001
Not lonely	9 116 (82.4%)	53 565 (90.5%)	174 338 (95.4%)	52 882 (97.1%)	
Lonely	1 950 (17.6%)	5 604 (9.5%)	8 361 (4.6%)	1 562 (2.9%)	
**Social isolation**					< 0.001
Not isolated	8 848 (80.0%)	52 191 (88.2%)	168 805 (92.4%)	51 087 (93.8%)	
Isolated	2 218 (20.0%)	6 978 (11.8%)	13 894 (7.6%)	3 357 (6.2%)	
Lifestyle factors					
**Smoking status**					< 0.001
Never	4 517 (40.8%)	28 086 (47.5%)	102 052 (55.9%)	33 820 (62.1%)	
Former	4 259 (38.5%)	22 211 (37.5%)	64 552 (35.3%)	17 616 (32.4%)	
Current	2 290 (20.7%)	8 872 (15.0%)	16 095 (8.8%)	3 008 (5.5%)	
**Stair climbing frequency**					< 0.001
None	1 830 (16.5%)	5 687 (9.6%)	13 311 (7.3%)	3 221 (5.9%)	
1-5/day	3 674 (33.2%)	13 892 (23.5%)	32 466 (17.8%)	8 235 (15.1%)	
6-10/day	3 263 (29.5%)	22 079 (37.3%)	70 409 (38.5%)	20 231 (37.2%)	
11-15/day	1 270 (11.5%)	9 948 (16.8%)	37 158 (20.3%)	11 939 (21.9%)	
16-20/day	568 (5.1%)	4 328 (7.3%)	16 862 (9.2%)	5 851 (10.7%)	
20+/day	461 (4.2%)	3 235 (5.5%)	12 493 (6.8%)	4 967 (9.1%)	
**Alcohol intake frequency**					< 0.001
Never	1 944 (17.6%)	5 319 (9.0%)	10 392 (5.7%)	2 768 (5.1%)	
Special occasions	2 081 (18.8%)	8 077 (13.7%)	17 346 (9.5%)	4 022 (7.4%)	
1-3/month	1 421 (12.8%)	7 222 (12.2%)	19 906 (10.9%)	5 249 (9.6%)	
1-2/week	2 298 (20.8%)	14 795 (25.0%)	47 880 (26.2%)	13 804 (25.4%)	
3-4/week	1 525 (13.8%)	12 256 (20.7%)	46 384 (25.4%)	15 086 (27.7%)	
Daily/almost daily	1 797 (16.2%)	11 500 (19.4%)	40 791 (22.3%)	13 515 (24.8%)	
**Sleep duration** (hours/day)					< 0.001
Mean (SD)	7.13 (1.80)	7.07 (1.19)	7.17 (0.98)	7.23 (0.92)	
Range	1.00-20.00	1.00-16.00	1.00-20.00	2.00-18.00	
**BMI** (kg/m^2)					< 0.001
Mean (SD)	30.68 (6.72)	29.13 (5.29)	27.00 (4.27)	25.46 (3.50)	
Range	12.80-67.30	13.80-66.00	12.80-63.40	14.90-51.90	
**Walking** (days/week)[3]					< 0.001
Mean (SD)	4.53 (2.44)	5.14 (2.06)	5.42 (1.88)	5.58 (1.84)	
Range	0.00-7.00	0.00-7.00	0.00-7.00	0.00-7.00	
**Moderate activity** (days/week)[3]					< 0.001
Mean (SD)	2.65 (2.52)	3.29 (2.38)	3.63 (2.28)	3.97 (2.26)	
Range	0.00-7.00	0.00-7.00	0.00-7.00	0.00-7.00	
**Vigorous activity** (days/week)[3]					< 0.001
Mean (SD)	0.98 (1.75)	1.46 (1.88)	1.89 (1.90)	2.45 (2.02)	
Range	0.00-7.00	0.00-7.00	0.00-7.00	0.00-7.00	
Environmental exposures					

**$PM_{2.5}$**					< 0.001
Mean (SD)	10.19 (1.07)	10.05 (1.04)	9.93 (1.03)	9.88 (1.05)	
Range	8.17-17.24	8.17-21.25	8.17-19.76	8.17-19.89	
**PM_{10}**					< 0.001
Mean (SD)	16.37 (1.83)	16.29 (1.85)	16.17 (1.88)	16.10 (1.91)	
Range	11.78-25.48	11.78-30.65	11.78-30.65	11.78-29.90	
**NO_2**					< 0.001
Mean (SD)	28.01 (7.53)	27.13 (7.43)	26.27 (7.51)	25.93 (7.77)	
Range	12.93-98.49	12.93-105.88	12.93-108.49	12.93-107.81	
**L_{den}**					< 0.001
Mean (SD)	56.17 (4.37)	56.08 (4.31)	55.99 (4.23)	55.97 (4.17)	
Range	51.56-84.41	51.56-89.29	51.55-86.50	51.55-84.65	
**Greenspace** 1000m					< 0.001
Mean (SD)	42.57 (20.15)	44.00 (20.81)	45.88 (21.81)	46.54 (22.81)	
Range	6.45-98.60	6.08-99.14	4.49-99.18	4.67-99.19	

Note: GCSEs = general certificate of secondary education; CSE = certificate of secondary education; NVQ = national vocational qualification; HND = higher national diploma; HNC = higher national certificate; BMI = body mass index; PM = particulate matter; NO_2 = nitrogen dioxide; L_{den} = day-evening-night noise level. [1]also includes 'other professional qualifications'. [2]Annual household income groups: very low (<£18,000), low (£18,000–£30,999), middle (£31,000–£51,999), high (£52,000–£100,000) and very high (>£100,000). [3]number of days per week engaging in these activities for 10+ minutes continuously.

Tables S6-S9. Visual summary of findings

Lifestyle factors

	Outcome					Health status						Long-standing illness						Self-rated health					
	Cross-sectional			Longitudinal		Sex			Age			Sex			Age			Sex			Age		
Variable	Health status	Long-standing illness	Self-rated health	Self-rated health t1	Self-rated health t2	Stratified (m/f)		Interaction term	Stratified (<65/65+)		Interaction term	Stratified (m/f)		Interaction term	Stratified (<65/65+)		Interaction term	Stratified (m/f)		Interaction term	Stratified (<65/65+)		Interaction term
**Sleep**	↓	--	↑	↑	↑	↓	--	√√	↓	--	ns	--	--	ns	--	--	√	↑	↑	ns	**↑**	↑	√√
**Physical activity**																							
Walking	↑	↑	↑	↑	↑	--	↑	ns	↑	↑	√√	↑	**↑**	√√	↑	**↑**	√√	↑	**↑**	√	↑	**↑**	√√
Moderate	--	--	↑	--	--	--	--	√	--	--	ns	--	↓	√	--	--	√√	↑	--	√√	↑	**↑**	ns
Vigorous	↑	↑	↑	↑	↑	↑	↑	√√	↑	↑	ns	↑	↑	ns	↑	↑	ns	**↑**	↑	√√	**↑**	↑	√√
**Stair climbing frequency**																							
None																							
1-5/day	↑	↑	↑	--	--	--	--	ns	↑	--	√√	--	↑	ns	↑	--	√√	--	--	ns	↑	--	√√
6-10/day	↑	↑	↑	--	--	↑	↑	√	↑	↑	√√	↑	**↑**	ns	↑	↑	√√	↑	↑	√	↑	↑	√
11-15/day	↑	↑	↑	--	--	↑	↑	√	↑	↑	√√	↑	**↑**	ns	↑	↑	√√	↑	↑	√√	↑	↑	ns
16-20/day	↑	↑	↑	--	--	↑	↑	ns	↑	↑	√	↑	**↑**	ns	↑	**↑**	ns	↑	↑	√√	↑	↑	ns
20+/day	↑	↑	↑	--	--	↑	↑	√√	↑	--	√√	↑	↑	ns	↑	↑	ns	**↑**	↑	√√	↑	↑	√
**Alcohol intake frequency**																							
Never	↓	↓	↓	↓	↓	↓	↓	√	**↓**	↓	√√	↓	**↓**	√√	**↓**	↓	√	↓	**↓**	√√	↓	↓	√√
Special	↓	↓	↓	↓	↓	↓	↓	ns	↓	↓	√√	↓	**↓**	ns	↓	↓	√√	↓	**↓**	ns	↓	↓	ns
1-3/month	↓	↓	↓	--	--	↓	↓	ns	↓	--	ns	↓	↓	ns	↓	↓	√	↓	↓	ns	↓	--	√
1-2/week																							
3-4/week	↑	↑	↑	--	--	↑	--	√	↑	--	ns	↑	↑	ns	↑	**↑**	ns	--	↑	ns	↑	**↑**	ns
Daily	--	↑	--	--	--	↑	--	√	--	--	ns	↑	↑	ns	↑	**↑**	ns	--	↑	ns	--	↑	√√
**BMI**	↓	↓	↓	↓	↓	**↓**	↓	√√	↓	**↓**	√√	**↓**	↓	√√	↓	↓	√√	**↓**	↓	√√	**↓**	↓	√√
**Smoking status**																							
Never																							
Former	↓	↓	↓	↓	↓	**↓**	↓	√√	↓	↓	√√	**↓**	↓	√√	↓	**↓**	√√	**↓**	↓	√√	↓	↓	ns
Current	↓	↓	↓	↓	↓	↓	**↓**	√	**↓**	↓	√	↓	**↓**	ns	**↓**	↓	ns	↓	↓	√√	**↓**	↓	√√

Table legend

↓	associated with unfavourable health
**↓**	associated with unfavourable health; stronger in this stratum
↑	associated with favourable health
**↑**	associated with favourable health; stronger in this stratum
--	no evidence of association with health
√√	statistically significant – Bonferroni correction
√	statistically significant – Benjamini & Hochberg correction
ns	not statistically significant

Environmental exposures

	Outcome					Health status				Long-standing illness				Self-rated health			
	Cross-sectional			Longitudinal		Sex		Age		Sex		Age		Sex		Age	
Variable	Health status	Long-standing illness	Self-rated health	Self-rated health t1	Self-rated health t2	Stratified (m/f)	Interaction term	Stratified (<65/65+)	Interaction term	Stratified (m/f)	Interaction term	Stratified (<65/65+)	Interaction term	Stratified (m/f)	Interaction term	Stratified (<65/65+)	Interaction term
**$PM_{2.5}$**	↓	↓	↑	--	--	↓ / --	✓	↓ / --	ns	↓ / --	ns	↓ / --	ns	-- / --	✓✓	↑ / --	✓✓
**PM_{10}**	--	--	--	--	--	-- / --	ns	-- / --	ns	-- / --	ns	-- / --	ns	-- / --	✓	-- / --	ns
**NO_2**	--	--	↓	--	--	-- / ↑	✓	-- / --	ns	-- / --	ns	-- / --	ns	-- / ↓	✓	-- / --	✓✓
**L_{den}**	--	--	--	--	--	-- / --	ns	-- / --	✓	-- / --	ns	-- / --	ns	-- / --	ns	-- / --	✓✓
**Greenspace**	--	--	--	--	--	-- / --	ns	-- / --	ns	-- / --	ns	-- / --	ns	-- / --	ns	-- / --	✓

Table legend

↓	associated with unfavourable health
↓	associated with unfavourable health; stronger in this stratum
↑	associated with favourable health
↑	associated with favourable health; stronger in this stratum
--	no evidence of association with health
✓✓	statistically significant – Bonferroni correction
✓	statistically significant – Benjamini & Hochberg correction
ns	not statistically significant

Psychosocial factors

Variable	Outcome					Health status				Long-standing illness				Self-rated health			
	Cross-sectional			Longitudinal		Sex		Age		Sex		Age		Sex		Age	
	Health status	Long-standing illness	Self-rated health	Self-rated health t1	Self-rated health t2	Stratified (m/f)	Interaction term	Stratified (<65/65+)	Interaction term	Stratified (m/f)	Interaction term	Stratified (<65/65+)	Interaction term	Stratified (m/f)	Interaction term	Stratified (<65/65+)	Interaction term
**Loneliness**																	
Not lonely																	
Lonely	↓	↓	↓	↓	↓	↓ ↓	✓✓	↓ --	✓	↓ ↓	✓✓	↓ ↓	ns	↓ ↓	✓✓	↓ ↓	✓✓
**Social isolation**																	
Not isolated																	
Isolated	↓	↓	↓	--	↓	↓ --	✓	↓ --	✓✓	↓ ↓	ns	↓ --	✓✓	↓ ↓	✓✓	↓ --	✓✓

Table legend

Symbol	Meaning
↓	associated with unfavourable health
↓	associated with unfavourable health; stronger in this stratum
↑	associated with favourable health
↑	associated with favourable health; stronger in this stratum
--	no evidence of association with health
✓✓	statistically significant – Bonferroni correction
✓	statistically significant – Benjamini & Hochberg correction
ns	not statistically significant

Sociodemographic characteristics

Outcome	Cross-sectional			Longitudinal		Health status				Long-standing illness				Self-rated health			
						Sex		Age		Sex		Age		Sex		Age	
Variable	Health status	Long-standing illness	Self-rated health	Self-rated health t1	Self-rated health t2	Stratified (m/f)	Interaction term	Stratified (<65/65+)	Interaction term	Stratified (m/f)	Interaction term	Stratified (<65/65+)	Interaction term	Stratified (m/f)	Interaction term	Stratified (<65/65+)	Interaction term
**Household income**																	
Very low	↓	↓	↓	↓	↓	↓ ↓	✓✓	↓ ↓	✓✓	↓ ↓	✓✓	↓ ↓	✓✓	↓ ↓	✓✓	↓ ↓	✓✓
Low	↓	↓	↓	--	↓	↓ ↓	✓✓	↓ --	ns	↓ ↓	✓✓	↓ --	✓	↓ ↓	✓	↓ ↓	✓
Middle																	
High	↑	↑	↑	↑	↑	-- --	✓	↑ --	✓	↑ ↑	ns	↑ --	ns	↑ ↑	✓✓	↑ --	✓✓
Very high	--	↑	↑	↑	↑	-- --	✓	-- --	ns	↑ ↑	ns	↑ --	ns	↑ ↑	✓✓	↑ --	✓✓
**Sex**																	
Female																	
Male	↓	↓	↓	↓	↓			↓ ↓	✓✓			↓ ↓	✓✓			↓ ↓	✓✓
**Age**	↓	↓	↑	--	↑	↓ ↓	✓✓	↓ ↓		↓ ↓	✓✓	↓ ↓		↑ ↑	✓✓	↑ --	
**Deprivation**	↓	↓	↓	--	↓	↓ ↓	✓✓	↓ ↓	ns	↓ ↓	✓	↓ ↓	ns	↓ ↓	✓✓	↓ ↓	ns
**Ethnicity**																	
White																	
Mixed-race	--	--	--	--	--	-- --	ns	-- --	ns	-- --	ns	-- --	ns	-- --	✓	-- --	ns
Asian	--	↑	↓	--	↓	-- ↑	✓	-- --	✓✓	↑ ↑	ns	↑ --	✓✓	↓ ↓	✓	↓ ↓	✓✓
Black	↑	↑	--	--	--	↑ ↑	ns	↑ --	ns	↑ --	✓✓	↑ --	✓✓	↑ --	✓✓	↑ ↓	✓✓
Chinese	↑	↑	↓	--	--	↑ ↑	ns	↑ --	ns	↑ ↑	ns	↑ --	ns	-- ↓	ns	↓ --	ns
Other	↑	↑	--	--	--	↑ --	ns	↑ --	ns	↑ --	ns	↑ --	✓✓	-- ↓	✓✓	-- ↓	✓✓
**Highest qualification**																	
None																	
GCSEs	--	--	↑	--	--	-- --	✓✓	-- --	ns	-- --	✓✓	-- --	✓✓	↑ ↑	✓	↑ ↑	✓✓
A levels	--	↓	↑	--	--	-- --	✓✓	-- --	✓	↓ ↓	✓✓	↓ ↓	✓✓	↑ ↑	✓✓	↑ ↑	✓✓
Degree	↓	↓	↑	--	↑	-- --	✓✓	-- --	✓	↓ ↓	✓✓	↓ ↓	✓✓	↑ ↑	✓✓	↑ ↑	✓✓

Table legend

Symbol	Meaning
↓	associated with unfavourable health
↓	associated with unfavourable health; stronger in this stratum
↑	associated with favourable health
↑	associated with favourable health; stronger in this stratum
--	no evidence of association with health
✓✓	statistically significant – Bonferroni correction
✓	statistically significant – Benjamini & Hochberg correction
ns	not statistically significant

Tables S10-S13. Regression tables long-standing illness

Sociodemographic characteristics

Table S10. **Sociodemographic characteristics associated with long-standing illness**

	Model 1			Model 2			Model 3			Model 4		
Term	OR	Bonferroni-corrected CI		OR	Bonferroni-corrected CI		OR	Bonferroni-corrected CI		OR	Bonferroni-corrected CI	
**Household income**[1]												
Very low	0.4914	0.4739	0.5094	0.5362	0.5166	0.5565	0.5965	0.5734	0.6206	0.6654	0.6384	0.6934
Low	0.7785	0.7515	0.8064	0.8323	0.8029	0.8628	0.8638	0.8329	0.8957	0.8906	0.8581	0.9244
Middle	Ref	–	–	Ref	–	–	Ref	–	–	Ref	–	–
High	1.2410	1.1936	1.2903	1.1933	1.1473	1.2412	1.1542	1.1091	1.2011	1.1171	1.0726	1.1634
Very high	1.5004	1.4059	1.6021	1.4327	1.3419	1.5305	1.3720	1.2837	1.4671	1.2524	1.1703	1.3410
**Sex**												
Female	Ref	–	–	Ref	–	–	Ref	–	–	Ref	–	–
Male	0.7788	0.7594	0.7987	–	–	–	0.7679	0.7482	0.7882	0.7585	0.7380	0.7797
**Age**	0.9644	0.9629	0.9660	–	–	–	0.9732	0.9715	0.9750	0.9730	0.9711	0.9748
**Multiple deprivation**	0.9845	0.9836	0.9854	0.9827	0.9818	0.9836	0.9881	0.9871	0.9891	0.9921	0.9910	0.9932
**Ethnicity**												
White	Ref	–	–	Ref	–	–	Ref	–	–	Ref	–	–
Mixed-race	1.1530	0.9729	1.3724	0.9368	0.7890	1.1172	1.0420	0.8755	1.2453	1.1223	0.9387	1.3474
Asian	1.0218	0.9227	1.1331	0.9252	0.8343	1.0273	1.0452	0.9412	1.1622	1.2225	1.0962	1.3652
Black	0.8780	0.7906	0.9763	0.7200	0.6474	0.8017	0.9670	0.8676	1.0791	1.1852	1.0591	1.3277
Chinese	1.7641	1.3404	2.3591	1.5038	1.1401	2.0150	1.5964	1.2071	2.1443	1.6205	1.2207	2.1842
Other	0.9554	0.8252	1.1091	0.8340	0.7191	0.9697	0.9925	0.8539	1.1565	1.2027	1.0301	1.4078
**Highest qualification**												
None	Ref	–	–	Ref	–	–	Ref	–	–	Ref	–	–
O levels/GCSEs/CSEs	1.5515	1.4891	1.6165	1.2693	1.2166	1.3241	1.0412	0.9965	1.0878	0.9920	0.9485	1.0375
A levels/NVQ/HND/HNC[2]	1.3935	1.3364	1.4531	1.2067	1.1562	1.2594	0.9484	0.9070	0.9915	0.8953	0.8554	0.9371
Degree	1.7157	1.6492	1.7849	1.4204	1.3635	1.4796	0.9827	0.9401	1.0272	0.8550	0.8167	0.8950

Note: Bonferroni-adjusted (~99.9%) confidence intervals. OR = odds ratio; CI = confidence interval; GCSEs = general certificate of secondary education; CSE = certificate of secondary education; NVQ = national vocational qualification; HND = higher national diploma; HNC = higher national certificate. [1]Annual household income groups: very low (<£18,000), low (£18,000–£30,999), middle (£31,000–£51,999), high (£52,000–£100,000) and very high (>£100,000). [2]also includes 'other professional qualifications'.
Model 1 – only individual explanatory variables.
Model 2 – adjusted for age and sex.
Model 3 – age, sex and all sociodemographic characteristics.
Model 4 – all explanatory variables.

Psychosocial factors

Table S11. **Psychosocial factors associated with long-standing illness**

	Model 1			Model 2			Model 3			Model 4		
Term	OR	Bonferroni-corrected CI		OR	Bonferroni-corrected CI		OR	Bonferroni-corrected CI		OR	Bonferroni-corrected CI	
**Loneliness**												
Not lonely	Ref	–	–	Ref	–	–	Ref	–	–	Ref	–	–
Lonely	0.5828	0.5539	0.6134	0.5658	0.5373	0.5958	0.5970	0.5667	0.6290	0.7097	0.6722	0.7494
**Social isolation**												
Not isolated	Ref	–	–	Ref	–	–	Ref	–	–	Ref	–	–
Isolated	0.6542	0.6269	0.6828	0.6558	0.6281	0.6847	0.6906	0.6611	0.7214	0.9185	0.8770	0.9621

Note: Bonferroni-adjusted (~99.9%) confidence intervals. OR = odds ratio; CI = confidence interval.

Model 1 – only individual explanatory variables.

Model 2 – adjusted for age and sex.

Model 3 – age, sex and all psychosocial factors.

Model 4 – all explanatory variables.

Table S12. **Lifestyle factors associated with long-standing illness**

	Model 1			Model 2			Model 3			Model 4		
Term	OR	Bonferroni-corrected CI		OR	Bonferroni-corrected CI		OR	Bonferroni-corrected CI		OR	Bonferroni-corrected CI	
**Sleep duration** (hours/day)	0.9984	0.9866	1.0104	1.0142	1.0020	1.0265	1.0014	0.9893	1.0137	1.0004	0.9882	1.0128
**Physical activity** (days/week)[1]												
Walking	1.0408	1.0341	1.0475	1.0505	1.0437	1.0573	1.0179	1.0106	1.0252	1.0250	1.0175	1.0325
Moderate activity	1.0319	1.0263	1.0376	1.0428	1.0370	1.0486	0.9927	0.9859	0.9994	0.9984	0.9916	1.0053
Vigorous activity	1.0917	1.0844	1.0991	1.0918	1.0844	1.0993	1.0688	1.0604	1.0774	1.0641	1.0557	1.0727
**Stair climbing frequency**												
None	Ref	–	–	Ref	–	–	Ref	–	–	Ref	–	–
1-5/day	1.2582	1.1962	1.3235	1.1326	1.0759	1.1923	1.1579	1.0984	1.2206	1.1101	1.0522	1.1710
6-10/day	1.6877	1.6100	1.7690	1.5338	1.4623	1.6086	1.4045	1.3373	1.4750	1.2937	1.2307	1.3598
11-15/day	1.8530	1.7600	1.9508	1.6718	1.5868	1.7612	1.4361	1.3612	1.5151	1.3131	1.2434	1.3866
16-20/day	1.9066	1.7941	2.0263	1.7183	1.6159	1.8275	1.4280	1.3406	1.5211	1.3066	1.2255	1.3932
20+/day	1.9605	1.8360	2.0939	1.7164	1.6061	1.8347	1.4113	1.3181	1.5113	1.2975	1.2108	1.3907
**Alcohol intake frequency**												
Never	0.4952	0.4701	0.5216	0.4959	0.4704	0.5227	0.5097	0.4829	0.5379	0.5533	0.5234	0.5850
Special occasions	0.6305	0.6028	0.6596	0.6119	0.5844	0.6406	0.6623	0.6319	0.6941	0.7142	0.6809	0.7492
1-3/month	0.8239	0.7876	0.8620	0.7846	0.7495	0.8213	0.8197	0.7824	0.8589	0.8486	0.8096	0.8895
1-2/week	Ref	–	–	Ref	–	–	Ref	–	–	Ref	–	–
3-4/week	1.1360	1.0949	1.1786	1.1808	1.1376	1.2257	1.1387	1.0963	1.1828	1.0925	1.0514	1.1353
Daily/almost daily	1.0289	0.9911	1.0682	1.1540	1.1108	1.1989	1.1374	1.0939	1.1828	1.0744	1.0326	1.1180
**BMI** (kg/m^2)	0.9259	0.9234	0.9284	0.9276	0.9250	0.9301	0.9387	0.9361	0.9414	0.9419	0.9392	0.9446
**Smoking status**												
Never	Ref	–	–	Ref	–	–	Ref	–	–	Ref	–	–
Former	0.7181	0.6989	0.7378	0.7898	0.7683	0.8119	0.7761	0.7542	0.7987	0.7978	0.7749	0.8213
Current	0.7016	0.6723	0.7322	0.6864	0.6574	0.7168	0.6888	0.6588	0.7202	0.7858	0.7506	0.8227

Note: Bonferroni-adjusted (~99.9%) confidence intervals. OR = odds ratio; CI = confidence interval; BMI = body mass index. [1]number of days per week engaging in these activities for 10+ minutes continuously.
Model 1 – only individual explanatory variables.
Model 2 – adjusted for age and sex.
Model 3 – age, sex and all lifestyle factors.
Model 4 – all explanatory variables.

Table S13. **Environmental exposures associated with long-standing illness**

	Model 1			Model 2			Model 3			Model 4		
Term	OR	Bonferroni-corrected CI		OR	Bonferroni-corrected CI		OR	Bonferroni-corrected CI		OR	Bonferroni-corrected CI	
**$PM_{2.5}$**	0.9254	0.9144	0.9367	0.9065	0.8955	0.9176	0.8969	0.8749	0.9194	0.9677	0.9430	0.9932
**PM_{10}**	0.9814	0.9748	0.9880	0.9762	0.9696	0.9828	1.0053	0.9970	1.0136	1.0019	0.9934	1.0105
**NO_2**	0.9917	0.9901	0.9933	0.9887	0.9870	0.9904	0.9971	0.9930	1.0011	1.0025	0.9982	1.0068
**L_{den}**	0.9971	0.9942	1.0001	0.9958	0.9928	0.9988	1.0049	1.0013	1.0084	1.0015	0.9979	1.0052
**Greenspace** 1000m	1.0017	1.0011	1.0022	1.0026	1.0020	1.0032	0.9986	0.9977	0.9996	0.9997	0.9987	1.0007

Note: Bonferroni-adjusted (~99.9%) confidence intervals. OR = odds ratio; CI = confidence interval; PM = particulate matter; NO_2 = nitrogen dioxide; L_{den} = day-evening-night noise level.
Model 1 – only individual explanatory variables.
Model 2 – adjusted for age and sex.
Model 3 – age, sex and all environmental exposures.
Model 4 – all explanatory variables.

Tables S14-S17. Regression tables self-rated health

Sociodemographic characteristics

Table S14. **Sociodemographic characteristics associated with self-rated health**

	Model 1			Model 2			Model 3			Model 4		
Term	OR	Bonferroni-corrected CI		OR	Bonferroni-corrected CI		OR	Bonferroni-corrected CI		OR	Bonferroni-corrected CI	
**Household income**[1]												
Very low	0.4830	0.4670	0.4997	0.4399	0.4248	0.4555	0.5696	0.5490	0.5909	0.6192	0.5960	0.6432
Low	0.8127	0.7873	0.8389	0.7668	0.7425	0.7919	0.8472	0.8201	0.8753	0.8512	0.8234	0.8799
Middle	Ref	–	–	Ref	–	–	Ref	–	–	Ref	–	–
High	1.3055	1.2628	1.3497	1.3572	1.3125	1.4034	1.2288	1.1877	1.2712	1.2102	1.1691	1.2526
Very high	1.9695	1.8690	2.0754	2.0726	1.9663	2.1846	1.7670	1.6748	1.8644	1.6364	1.5491	1.7286
**Sex**												
Female	Ref	–	–	Ref	–	–	Ref	–	–	Ref	–	–
Male	0.8049	0.7867	0.8236	–	–	–	0.7601	0.7426	0.7780	0.7836	0.7648	0.8030
**Age**	0.9954	0.9940	0.9968	–	–	–	1.0100	1.0084	1.0116	1.0113	1.0097	1.0130
**Multiple deprivation**	0.9771	0.9762	0.9779	0.9768	0.9760	0.9777	0.9859	0.9850	0.9868	0.9923	0.9913	0.9933
**Ethnicity**												
White	Ref	–	–	Ref	–	–	Ref	–	–	Ref	–	–
Mixed-race	0.8044	0.6916	0.9360	0.7644	0.6570	0.8898	0.8341	0.7162	0.9718	0.9019	0.7729	1.0527
Asian	0.5267	0.4811	0.5768	0.5270	0.4813	0.5773	0.5714	0.5212	0.6266	0.5995	0.5445	0.6602
Black	0.6258	0.5683	0.6893	0.6025	0.5469	0.6639	0.8382	0.7595	0.9253	1.0896	0.9839	1.2069
Chinese	0.8293	0.6673	1.0318	0.7950	0.6396	0.9894	0.7954	0.6386	0.9915	0.6783	0.5413	0.8506
Other	0.6770	0.5922	0.7745	0.6604	0.5774	0.7557	0.7479	0.6529	0.8570	0.8903	0.7746	1.0235
**Highest qualification**												
None	Ref	–	–	Ref	–	–	Ref	–	–	Ref	–	–
O levels/GCSEs/CSEs	1.5682	1.5094	1.6292	1.5801	1.5193	1.6434	1.2710	1.2208	1.3232	1.2420	1.1920	1.2941
A levels/NVQ/HND/HNC[2]	1.7160	1.6499	1.7849	1.7435	1.6753	1.8146	1.3336	1.2797	1.3896	1.2876	1.2345	1.3429
Degree	2.5396	2.4468	2.6360	2.5944	2.4968	2.6958	1.7178	1.6490	1.7895	1.5031	1.4412	1.5676

Note: Bonferroni-adjusted (~99.9%) confidence intervals. OR = odds ratio; CI = confidence interval; GCSEs = general certificate of secondary education; CSE = certificate of secondary education; NVQ = national vocational qualification; HND = higher national diploma; HNC = higher national certificate. For categorical explanatory variables the odds ratios indicate the changes in odds of reporting better self-rated health associated with the explanatory variable group relative to the reference group. Odds ratios for continuous explanatory variables indicate proportional odds ratios for a 1-unit increase in the explanatory variable on level of self-rated health. [1]Annual household income groups: very low (<£18,000), low (£18,000–£30,999), middle (£31,000–£51,999), high (£52,000–£100,000) and very high (>£100,000). [2]also includes 'other professional qualifications'.
Model 1 – only individual explanatory variables.
Model 2 – adjusted for age and sex.
Model 3 – age, sex and all sociodemographic characteristics.
Model 4 – all explanatory variables.

Table S15. **Psychosocial factors associated with self-rated health**

	Model 1			Model 2			Model 3			Model 4		
Term	OR	Bonferroni-corrected CI		OR	Bonferroni-corrected CI		OR	Bonferroni-corrected CI		OR	Bonferroni-corrected CI	
**Loneliness**												
Not lonely	Ref	–	–	Ref	–	–	Ref	–	–	Ref	–	–
Lonely	0.3590	0.3419	0.3769	0.3589	0.3419	0.3769	0.3860	0.3675	0.4055	0.4921	0.4678	0.5176
**Social isolation**												
Not isolated	Ref	–	–	Ref	–	–	Ref	–	–	Ref	–	–
Isolated	0.5374	0.5159	0.5598	0.5394	0.5178	0.5619	0.5905	0.5667	0.6153	0.8593	0.8233	0.8970

Note: Bonferroni-adjusted (~99.9%) confidence intervals. OR = odds ratio; CI = confidence interval. For categorical explanatory variables the odds ratios indicate the changes in odds of reporting better self-rated health associated with the explanatory variable group relative to the reference group.
Model 1 – only individual explanatory variables.
Model 2 – adjusted for age and sex.
Model 3 – age, sex and all psychosocial factors.
Model 4 – all explanatory variables.

Table S16. **Lifestyle factors associated with self-rated health**

	Model 1			Model 2			Model 3			Model 4		
Term	OR	Bonferroni-corrected CI		OR	Bonferroni-corrected CI		OR	Bonferroni-corrected CI		OR	Bonferroni-corrected CI	
**Sleep duration** (hours/day)	1.0959	1.0836	1.1082	1.0958	1.0836	1.1082	1.0781	1.0660	1.0903	1.0730	1.0609	1.0852
**Physical activity** (days/week)[1]												
Walking	1.1044	1.0979	1.1110	1.1044	1.0978	1.1110	1.0394	1.0326	1.0461	1.0522	1.0454	1.0591
Moderate activity	1.1009	1.0954	1.1064	1.1029	1.0974	1.1085	1.0010	0.9949	1.0071	1.0132	1.0070	1.0195
Vigorous activity	1.2033	1.1960	1.2106	1.2126	1.2052	1.2200	1.1715	1.1632	1.1799	1.1668	1.1585	1.1751
**Stair climbing frequency**												
None	Ref	–	–	Ref	–	–	Ref	–	–	Ref	–	–
1-5/day	1.0734	1.0225	1.1269	1.0717	1.0205	1.1254	1.1313	1.0766	1.1888	1.0533	1.0019	1.1074
6-10/day	1.5855	1.5157	1.6586	1.5775	1.5077	1.6506	1.4181	1.3544	1.4848	1.2293	1.1733	1.2878
11-15/day	1.8892	1.7997	1.9831	1.8694	1.7804	1.9628	1.5192	1.4457	1.5964	1.2892	1.2260	1.3557
16-20/day	2.0255	1.9150	2.1424	1.9988	1.8893	2.1146	1.5394	1.4537	1.6302	1.2991	1.2258	1.3767
20+/day	2.1944	2.0663	2.3305	2.1546	2.0282	2.2889	1.5905	1.4955	1.6915	1.3652	1.2828	1.4529
**Alcohol intake frequency**												
Never	0.5340	0.5078	0.5615	0.5201	0.4945	0.5470	0.5463	0.5190	0.5750	0.6624	0.6285	0.6981
Special occasions	0.6048	0.5799	0.6309	0.5743	0.5504	0.5992	0.6556	0.6279	0.6845	0.7565	0.7240	0.7904
1-3/month	0.8268	0.7934	0.8616	0.8007	0.7683	0.8345	0.8699	0.8343	0.9070	0.9201	0.8821	0.9597
1-2/week	Ref	–	–	Ref	–	–	Ref	–	–	Ref	–	–
3-4/week	1.2045	1.1663	1.2441	1.2297	1.1906	1.2701	1.1591	1.1217	1.1978	1.0520	1.0178	1.0875
Daily/almost daily	1.1495	1.1119	1.1884	1.2013	1.1617	1.2423	1.1793	1.1395	1.2204	1.0264	0.9913	1.0628
**BMI** (kg/m^2)	0.8850	0.8827	0.8873	0.8860	0.8838	0.8883	0.8994	0.8970	0.9018	0.9045	0.9020	0.9069
**Smoking status**												
Never	Ref	–	–	Ref	–	–	Ref	–	–	Ref	–	–
Former	0.7546	0.7361	0.7735	0.7741	0.7549	0.7938	0.7708	0.7512	0.7909	0.8128	0.7919	0.8342
Current	0.4133	0.3973	0.4298	0.4192	0.4030	0.4361	0.4057	0.3896	0.4226	0.5029	0.4825	0.5242

Note: Bonferroni-adjusted (~99.9%) confidence intervals. OR = odds ratio; CI = confidence interval; BMI = body mass index. For categorical explanatory variables the odds ratios indicate the changes in odds of reporting better self-rated health associated with the explanatory variable group relative to the reference group. Odds ratios for continuous explanatory variables indicate proportional odds ratios for a 1-unit increase in the explanatory variable on level of self-rated health. [1]number of days per week engaging in these activities for 10+ minutes continuously.
Model 1 – only individual explanatory variables.
Model 2 – adjusted for age and sex.
Model 3 – age, sex and all lifestyle factors.
Model 4 – all explanatory variables.

Table S17. **Environmental exposures associated with self-rated health**

	Model 1			Model 2			Model 3			Model 4		
Term	OR	Bonferroni-corrected CI		OR	Bonferroni-corrected CI		OR	Bonferroni-corrected CI		OR	Bonferroni-corrected CI	
**$PM_{2.5}$**	0.8860	0.8762	0.8958	0.8829	0.8732	0.8927	0.9192	0.8988	0.9400	1.0283	1.0047	1.0525
**PM_{10}**	0.9628	0.9570	0.9687	0.9621	0.9562	0.9679	0.9963	0.9890	1.0037	0.9979	0.9904	1.0055
**NO_2**	0.9843	0.9829	0.9858	0.9838	0.9823	0.9853	0.9906	0.9870	0.9942	0.9948	0.9910	0.9986
**L_{den}**	0.9951	0.9924	0.9977	0.9950	0.9923	0.9976	1.0072	1.0041	1.0104	1.0025	0.9993	1.0058
**Greenspace** 1000m	1.0039	1.0034	1.0044	1.0041	1.0035	1.0046	0.9990	0.9981	0.9998	1.0007	0.9998	1.0015

Note: Bonferroni-adjusted (~99.9%) confidence intervals. OR = odds ratio; CI = confidence interval; PM = particulate matter; NO_2 = nitrogen dioxide; L_{den} = day-evening-night noise level. Odds ratios indicate proportional odds ratios for a 1-unit increase in the explanatory variable on level of self-rated health.
Model 1 – only individual explanatory variables.
Model 2 – adjusted for age and sex.
Model 3 – age, sex and all environmental exposures.
Model 4 – all explanatory variables.

Tables S18-S21. Regression tables health indicators

Sociodemographic characteristics

Table S18. **Sociodemographic characteristics associated with health indicators**

	Health status			Long-standing illness			Self-rated health		
Term	OR	Bonferroni-corrected CI		OR	Bonferroni-corrected CI		OR[3]	Bonferroni-corrected CI	
**Household income**[1]									
Very low	0.7447	0.7148	0.7759	0.6654	0.6384	0.6934	0.6192	0.5960	0.6432
Low	0.9196	0.8864	0.9541	0.8906	0.8581	0.9244	0.8512	0.8234	0.8799
Middle	Ref	–	–	Ref	–	–	Ref	–	–
High	1.0510	1.0096	1.0941	1.1171	1.0726	1.1634	1.2102	1.1691	1.2526
Very high	1.0472	0.9812	1.1181	1.2524	1.1703	1.3410	1.6364	1.5491	1.7286
**Sex**									
Female	Ref	–	–	Ref	–	–	Ref	–	–
Male	0.8847	0.8610	0.9090	0.7585	0.7380	0.7797	0.7836	0.7648	0.8030
**Age**	0.9528	0.9510	0.9546	0.9730	0.9711	0.9748	1.0113	1.0097	1.0130
**Multiple deprivation**	0.9952	0.9941	0.9963	0.9921	0.9910	0.9932	0.9923	0.9913	0.9933
**Ethnicity**									
White	Ref	–	–	Ref	–	–	Ref	–	–
Mixed-race	1.1037	0.9221	1.3273	1.1223	0.9387	1.3474	0.9019	0.7729	1.0527
Asian	1.0188	0.9149	1.1359	1.2225	1.0962	1.3652	0.5995	0.5445	0.6602
Black	1.3125	1.1657	1.4805	1.1852	1.0591	1.3277	1.0896	0.9839	1.2069
Chinese	1.8339	1.3646	2.5106	1.6205	1.2207	2.1842	0.6783	0.5413	0.8506
Other	1.2570	1.0727	1.4779	1.2027	1.0301	1.4078	0.8903	0.7746	1.0235
**Highest qualification**									
None	Ref	–	–	Ref	–	–	Ref	–	–
O levels/GCSEs/CSEs	1.0315	0.9870	1.0779	0.9920	0.9485	1.0375	1.2420	1.1920	1.2941
A levels/NVQ/HND/HNC[2]	0.9879	0.9445	1.0332	0.8953	0.8554	0.9371	1.2876	1.2345	1.3429
Degree	0.9558	0.9137	0.9997	0.8550	0.8167	0.8950	1.5031	1.4412	1.5676

Note: Estimates from Model 4 (i.e. including all explanatory variables). Bonferroni-adjusted (~99.9%) confidence intervals. OR = odds ratio; CI = confidence interval; GCSEs = general certificate of secondary education; CSE = certificate of secondary education; NVQ = national vocational qualification; HND = higher national diploma; HNC = higher national certificate. [1]Annual household income groups: very low (<£18,000), low (£18,000–£30,999), middle (£31,000–£51,999), high (£52,000–£100,000) and very high (>£100,000). [2]also includes 'other professional qualifications'. [3]For categorical explanatory variables the odds ratios indicate the changes in odds of reporting better self-rated health associated with the explanatory variable group relative to the reference group. Odds ratios for continuous explanatory variables indicate proportional odds ratios for a 1-unit increase in the explanatory variable on level of self-rated health.

Table S19. **Psychosocial factors associated with health indicators**

	Health status			Long-standing illness			Self-rated health		
Term	OR	Bonferroni-corrected CI		OR	Bonferroni-corrected CI		OR[1]	Bonferroni-corrected CI	
**Loneliness**									
Not lonely	Ref	–	–	Ref	–	–	Ref	–	–
Lonely	0.8129	0.7697	0.8587	0.7097	0.6722	0.7494	0.4921	0.4678	0.5176
**Social isolation**									
Not isolated	Ref	–	–	Ref	–	–	Ref	–	–
Isolated	0.9496	0.9067	0.9947	0.9185	0.8770	0.9621	0.8593	0.8233	0.8970

Note: Estimates from Model 4 (i.e. including all explanatory variables). Bonferroni-adjusted (~99.9%) confidence intervals. OR = odds ratio; CI = confidence interval. [1]For categorical explanatory variables the odds ratios indicate the changes in odds of reporting better self-rated health associated with the explanatory variable group relative to the reference group.

Table S20. **Lifestyle factors associated with health indicators**

	Health status			Long-standing illness			Self-rated health		
Term	OR	Bonferroni-corrected CI		OR	Bonferroni-corrected CI		OR[2]	Bonferroni-corrected CI	
**Sleep duration** (hours/day)	0.9748	0.9631	0.9868	1.0004	0.9882	1.0128	1.0730	1.0609	1.0852
**Physical activity** (days/week)[1]									
Walking	1.0105	1.0032	1.0178	1.0250	1.0175	1.0325	1.0522	1.0454	1.0591
Moderate activity	1.0032	0.9964	1.0100	0.9984	0.9916	1.0053	1.0132	1.0070	1.0195
Vigorous activity	1.0319	1.0238	1.0400	1.0641	1.0557	1.0727	1.1668	1.1585	1.1751
**Stair climbing frequency**									
None	Ref	–	–	Ref	–	–	Ref	–	–
1-5/day	1.0704	1.0148	1.1289	1.1101	1.0522	1.1710	1.0533	1.0019	1.1074
6-10/day	1.1756	1.1188	1.2352	1.2937	1.2307	1.3598	1.2293	1.1733	1.2878
11-15/day	1.1714	1.1098	1.2363	1.3131	1.2434	1.3866	1.2892	1.2260	1.3557
16-20/day	1.1650	1.0937	1.2410	1.3066	1.2255	1.3932	1.2991	1.2258	1.3767
20+/day	1.1881	1.1097	1.2723	1.2975	1.2108	1.3907	1.3652	1.2828	1.4529
**Alcohol intake frequency**									
Never	0.6285	0.5946	0.6644	0.5533	0.5234	0.5850	0.6624	0.6285	0.6981
Special occasions	0.7792	0.7429	0.8174	0.7142	0.6809	0.7492	0.7565	0.7240	0.7904
1-3/month	0.9129	0.8708	0.9570	0.8486	0.8096	0.8895	0.9201	0.8821	0.9597
1-2/week	Ref	–	–	Ref	–	–	Ref	–	–
3-4/week	1.0590	1.0198	1.0998	1.0925	1.0514	1.1353	1.0520	1.0178	1.0875
Daily/almost daily	1.0334	0.9939	1.0745	1.0744	1.0326	1.1180	1.0264	0.9913	1.0628
**BMI** (kg/m^2)	0.9683	0.9656	0.9711	0.9419	0.9392	0.9446	0.9045	0.9020	0.9069
**Smoking status**									
Never	Ref	–	–	Ref	–	–	Ref	–	–
Former	0.7922	0.7698	0.8152	0.7978	0.7749	0.8213	0.8128	0.7919	0.8342
Current	0.7518	0.7183	0.7869	0.7858	0.7506	0.8227	0.5029	0.4825	0.5242

Note: Estimates from Model 4 (i.e. including all explanatory variables). Bonferroni-adjusted (~99.9%) confidence intervals. OR = odds ratio; CI = confidence interval; BMI = body mass index. [1]number of days per week engaging in these activities for 10+ minutes continuously. [2]For categorical explanatory variables the odds ratios indicate the changes in odds of reporting better self-rated health associated with the explanatory variable group relative to the reference group. Odds ratios for continuous explanatory variables indicate proportional odds ratios for a 1-unit increase in the explanatory variable on level of self-rated health.

Table S21. **Environmental exposures associated with health indicators**

	Health status			Long-standing illness			Self-rated health		
Term	OR	Bonferroni-corrected CI		OR	Bonferroni-corrected CI		OR[1]	Bonferroni-corrected CI	
**$PM_{2.5}$**	0.9656	0.9411	0.9908	0.9677	0.9430	0.9932	1.0283	1.0047	1.0525
**PM_{10}**	1.0019	0.9935	1.0104	1.0019	0.9934	1.0105	0.9979	0.9904	1.0055
**NO_2**	1.0036	0.9993	1.0078	1.0025	0.9982	1.0068	0.9948	0.9910	0.9986
**L_{den}**	0.9981	0.9945	1.0017	1.0015	0.9979	1.0052	1.0025	0.9993	1.0058
**Greenspace** 1000m	0.9995	0.9985	1.0004	0.9997	0.9987	1.0007	1.0007	0.9998	1.0015

Note: Estimates from Model 4 (i.e. including all explanatory variables). Bonferroni-adjusted (~99.9%) confidence intervals. OR = odds ratio; CI = confidence interval; PM = particulate matter; NO_2 = nitrogen dioxide; L_{den} = day-evening-night noise level. [1]Odds ratios indicate proportional odds ratios for a 1-unit increase in the explanatory variable on level of self-rated health.

Tables S22-S24. Standardised regression coefficients tables

Health status

Table S22. **Standardised regression coefficients health status**

Term	*β*	Bonferroni-corrected CI	
Sociodemographic characteristics			
**Household income**[1]			
Very low	-0.2948	-0.3358	-0.2538
Low	-0.0838	-0.1206	-0.047
Middle	Ref	–	–
High	0.0497	0.0095	0.0899
Very high	0.0461	-0.019	0.1116
**Sex**			
Female	Ref	–	–
Male	-0.1225	-0.1496	-0.0954
**Age**	-0.7801	-0.811	-0.7493
**Multiple deprivation**	-0.1295	-0.1591	-0.0998
**Ethnicity**			
White	Ref	–	–
Mixed-race	0.0987	-0.0811	0.2831
Asian	0.0187	-0.0889	0.1274
Black	0.2719	0.1533	0.3924
Chinese	0.6065	0.3109	0.9205
Other	0.2287	0.0702	0.3906
**Highest qualification**			
None	Ref	–	–
O levels/GCSEs/CSEs	0.031	-0.0131	0.075
A levels/NVQ/HND/HNC[2]	-0.0122	-0.0571	0.0326
Degree	-0.0453	-0.0902	-0.0003
Psychosocial factors			
**Loneliness**			
Not lonely	Ref	–	–
Lonely	-0.2072	-0.2618	-0.1524
**Social isolation**			
Not isolated	Ref	–	–
Isolated	-0.0517	-0.098	-0.0053
Lifestyle factors			
**Sleep duration** (hours/day)	-0.0538	-0.0794	-0.0281
**Physical activity** (days/week)[3]			
Walking	0.0406	0.0124	0.0687
Moderate activity	0.0147	-0.0168	0.0462
Vigorous activity	0.1218	0.0914	0.1523
**Stair climbing frequency**			
None	Ref	–	–
1-5/day	0.068	0.0147	0.1213
6-10/day	0.1618	0.1122	0.2113
11-15/day	0.1581	0.1042	0.2121
16-20/day	0.1527	0.0896	0.2159
20+/day	0.1724	0.1041	0.2408
**Alcohol intake frequency**			
Never	-0.4644	-0.5198	-0.4089
Special occasions	-0.2494	-0.2972	-0.2016
1-3/month	-0.0911	-0.1384	-0.0439
1-2/week	Ref	–	–
3-4/week	0.0574	0.0196	0.0952
Daily/almost daily	0.0329	-0.0062	0.0718
**BMI** (kg/m^2)	-0.3001	-0.3267	-0.2735
**Smoking status**			
Never	Ref	–	–
Former	-0.233	-0.2616	-0.2043
Current	-0.2853	-0.3309	-0.2396
Environmental exposures			
**$PM_{2.5}$**	-0.0728	-0.1261	-0.0193
**PM_{10}**	0.007	-0.0246	0.0388
**NO_2**	0.0538	-0.0103	0.1177
**L_{den}**	-0.0162	-0.0466	0.0143
**Greenspace** 1000m	-0.023	-0.0652	0.0192

Note: β = Model 4 regression coefficients rescaled to have a mean equal to zero and, for numeric variables with more than two values, divided by two standard deviations. Bonferroni-adjusted (~99.9%) confidence intervals. CI = confidence interval; GCSEs = general certificate of secondary education; CSE = certificate of secondary education; NVQ = national vocational qualification; HND = higher national diploma; HNC = higher national certificate; BMI = body mass index; PM = particulate matter; NO_2 = nitrogen dioxide; L_{den} = day-evening-night noise level. [1]Annual household income groups: very low (<£18,000), low (£18,000–£30,999), middle (£31,000–£51,999), high (£52,000–£100,000) and very high (>£100,000). [2]also includes 'other professional qualifications'. [3]number of days per week engaging in these activities for 10+ minutes continuously.

Long-standing illness

Table S23. **Standardised regression coefficients for long-standing illness**

Term	*β*	Bonferroni-adjusted CI	
Sociodemographic characteristics			
**Household income**[1]			
Very low	-0.4074	-0.4487	-0.3661
Low	-0.1158	-0.153	-0.0786
Middle	Ref	–	–
High	0.1107	0.0701	0.1514
Very high	0.2251	0.1573	0.2934
**Sex**			
Female	Ref	–	–
Male	-0.2763	-0.3038	-0.2489
**Age**	-0.4423	-0.4729	-0.4117
**Multiple deprivation**	-0.2125	-0.2421	-0.1828
**Ethnicity**			
White	Ref	–	–
Mixed-race	0.1154	-0.0632	0.2982
Asian	0.2009	0.0918	0.3113
Black	0.1699	0.0574	0.2835
Chinese	0.4827	0.1994	0.7812
Other	0.1846	0.0297	0.3421
**Highest qualification**			
None	Ref	–	–
O levels/GCSEs/CSEs	-0.008	-0.0529	0.0368
A levels/NVQ/HND/HNC[2]	-0.1106	-0.1562	-0.0649
Degree	-0.1567	-0.2025	-0.1109
Psychosocial factors			
**Loneliness**			
Not lonely	Ref	–	–
Lonely	-0.3429	-0.3971	-0.2885
**Social isolation**			
Not isolated	Ref	–	–
Isolated	-0.085	-0.1313	-0.0387
Lifestyle factors			
**Sleep duration** (hours/day)	0.0009	-0.025	0.0268
**Physical activity** (days/week)[3]			
Walking	0.096	0.0677	0.1243
Moderate activity	-0.0074	-0.0393	0.0245
Vigorous activity	0.2413	0.2103	0.2724
**Stair climbing frequency**			
None	Ref	–	–
1-5/day	0.1044	0.0509	0.1579
6-10/day	0.2575	0.2076	0.3073
11-15/day	0.2724	0.2179	0.3269
16-20/day	0.2675	0.2034	0.3316
20+/day	0.2605	0.1913	0.3298
**Alcohol intake frequency**			
Never	-0.5918	-0.6474	-0.5361
Special occasions	-0.3366	-0.3844	-0.2888
1-3/month	-0.1642	-0.2113	-0.1171
1-2/week	Ref	–	–
3-4/week	0.0885	0.0501	0.1269
Daily/almost daily	0.0718	0.032	0.1115
**BMI** (kg/m^2)	-0.5586	-0.5855	-0.5317
**Smoking status**			
Never	Ref	–	–
Former	-0.2259	-0.255	-0.1969
Current	-0.2411	-0.2868	-0.1952
Environmental exposures			
**$PM_{2.5}$**	-0.0682	-0.1221	-0.0142
**PM_{10}**	0.0072	-0.0249	0.0393
**NO_2**	0.0381	-0.0266	0.1029
**L_{den}**	0.0127	-0.0181	0.0436
**Greenspace** 1000m	-0.0133	-0.0562	0.0295

Note: β = Model 4 regression coefficients rescaled to have a mean equal to zero and, for numeric variables with more than two values, divided by two standard deviations. Bonferroni-adjusted (~99.9%) confidence intervals. CI = confidence interval; GCSEs = general certificate of secondary education; CSE = certificate of secondary education; NVQ = national vocational qualification; HND = higher national diploma; HNC = higher national certificate; BMI = body mass index; PM = particulate matter; NO_2 = nitrogen dioxide; L_{den} = day-evening-night noise level. [1]Annual household income groups: very low (<£18,000), low (£18,000–£30,999), middle (£31,000–£51,999), high (£52,000–£100,000) and very high (>£100,000). [2]also includes 'other professional qualifications'. [3]number of days per week engaging in these activities for 10+ minutes continuously.

Self-rated health

Table S24. **Standardised regression coefficients for self-rated health**

Term	*β*	Bonferroni-corrected CI	
Sociodemographic characteristics			
**Household income**[1]			
Very low	-0.4794	-0.5174	-0.4412
Low	-0.1611	-0.1943	-0.128
Middle	Ref	–	–
High	0.1908	0.1563	0.2252
Very high	0.4925	0.444	0.5477
**Sex**			
Female	Ref	–	–
Male	-0.2438	-0.2682	-0.2194
**Age**	0.1819	0.1552	0.2086
**Multiple deprivation**	-0.2054	-0.2325	-0.1783
**Ethnicity**			
White	Ref	–	–
Mixed-race	-0.1034	-0.2572	0.0508
Asian	-0.5116	-0.6077	-0.4153
Black	0.0858	-0.016	0.1879
Chinese	-0.3881	-0.6128	-0.163
Other	-0.1163	-0.2553	0.0229
**Highest qualification**			
None	Ref	–	–
O levels/GCSEs/CSEs	0.2167	0.1761	0.2578
A levels/NVQ/HND/HNC[2]	0.2528	0.211	0.2947
Degree	0.4075	0.3655	0.4495
Psychosocial factors			
**Loneliness**			
Not lonely	Ref	–	–
Lonely	-0.7092	-0.7596	-0.6587
**Social isolation**			
Not isolated	Ref	–	–
Isolated	-0.1516	-0.1944	-0.1087
Lifestyle factors			
**Sleep duration** (hours/day)	0.1487	0.1248	0.1726
**Physical activity** (days/week)[3]			
Walking	0.198	0.1726	0.2234
Moderate activity	0.061	0.0326	0.0895
Vigorous activity	0.5988	0.5711	0.6266
**Stair climbing frequency**			
None	Ref	–	–
1-5/day	0.052	0.0019	0.102
6-10/day	0.2064	0.1599	0.2529
11-15/day	0.2541	0.2037	0.3043
16-20/day	0.2617	0.2037	0.3196
20+/day	0.3113	0.2489	0.3738
**Alcohol intake frequency**			
Never	-0.4119	-0.4631	-0.3607
Special occasions	-0.2791	-0.3229	-0.2352
1-3/month	-0.0833	-0.1254	-0.0411
1-2/week	Ref	–	–
3-4/week	0.0507	0.0177	0.0837
Daily/almost daily	0.0261	-0.0093	0.0609
**BMI** (kg/m^2)	-0.9368	-0.9616	-0.912
**Smoking status**			
Never	Ref	–	–
Former	-0.2073	-0.2332	-0.1813
Current	-0.6873	-0.7287	-0.6459
Environmental exposures			
**$PM_{2.5}$**	0.0581	0.01	0.1064
**PM_{10}**	-0.0077	-0.0359	0.0206
**NO_2**	-0.0784	-0.1362	-0.0207
**L_{den}**	0.0214	-0.0059	0.0487
**Greenspace** 1000m	0.0289	-0.0089	0.0666

Note: β = Model 4 regression coefficients rescaled to have a mean equal to zero and, for numeric variables with more than two values, divided by two standard deviations. Bonferroni-adjusted (~99.9%) confidence intervals. CI = confidence interval; GCSEs = general certificate of secondary education; CSE = certificate of secondary education; NVQ = national vocational qualification; HND = higher national diploma; HNC = higher national certificate; BMI = body mass index; PM = particulate matter; NO_2 = nitrogen dioxide; L_{den} = day-evening-night noise level. [1]Annual household income groups: very low (<£18,000), low (£18,000–£30,999), middle (£31,000–£51,999), high (£52,000–£100,000) and very high (>£100,000). [2]also includes 'other professional qualifications'. [3]number of days per week engaging in these activities for 10+ minutes continuously.

Figures S5-S6. Standardised regression coefficients plots

Long-standing illness

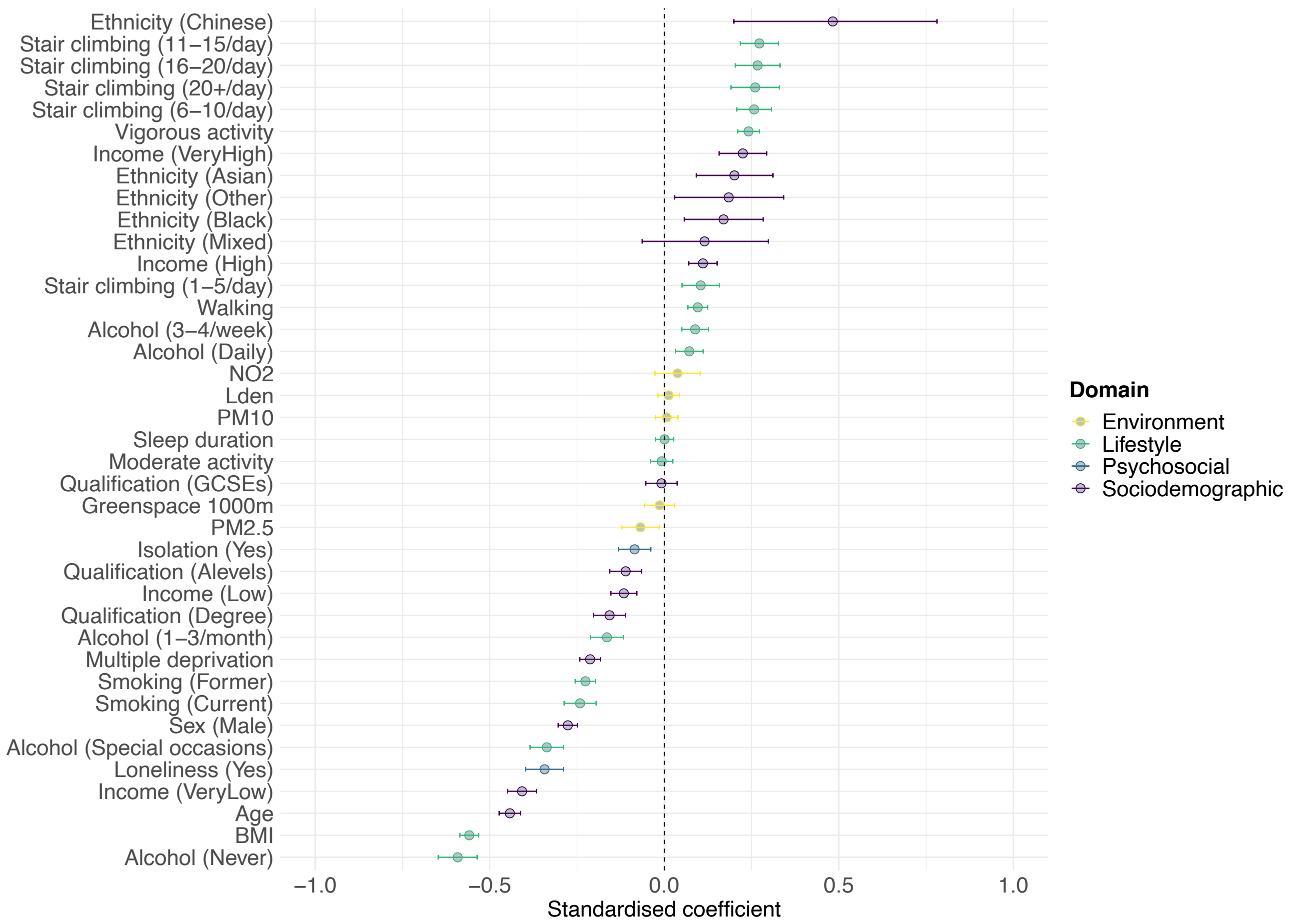

Figure S5. Confidence interval plot for long-standing illness. Standardised β estimates and Bonferroni-adjusted (~99.9%) confidence intervals. Model 4 regression coefficients were rescaled to have a mean equal to zero and, for numeric variables with more than two values, divided by two standard deviations.

Self-rated health

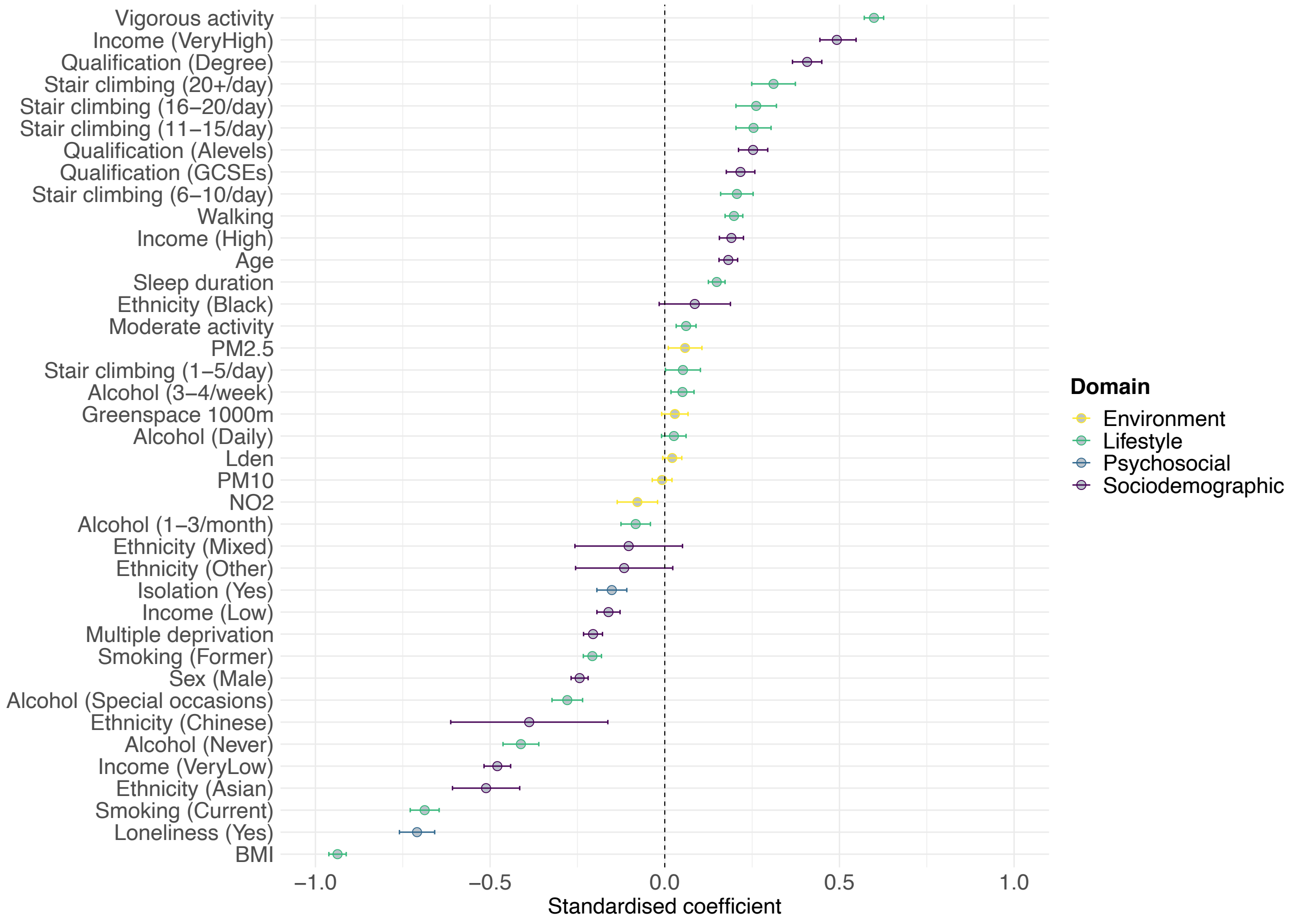

Figure S6. Confidence interval plot for self-rated health. Standardised β estimates and Bonferroni-adjusted (~99.9%) confidence intervals. Model 4 regression coefficients were rescaled to have a mean equal to zero and, for numeric variables with more than two values, divided by two standard deviations.

Table S25. Baseline characteristics stratified by sex

Table S25. **Baseline characteristics stratified by sex**

	Female (*n* = 159 574)	Male (*n* = 147 804)	p value
Health indicators			
**Health status**			< 0.001
Unhealthy	47 016 (29.5%)	48 161 (32.6%)	
Healthy	112 558 (70.5%)	99 643 (67.4%)	
**Long-standing illness**			< 0.001
Yes	44 607 (28.0%)	49 150 (33.3%)	
No	114 967 (72.0%)	98 654 (66.7%)	
**Self-rated health**			< 0.001
Poor	4 991 (3.1%)	6 075 (4.1%)	
Fair	27 657 (17.3%)	31 512 (21.3%)	
Good	97 197 (60.9%)	85 502 (57.8%)	
Excellent	29 729 (18.6%)	24 715 (16.7%)	
Sociodemographic characteristics			
**Age**			< 0.001
Mean (SD)	55.68 (7.96)	56.56 (8.16)	
Range	39.00-71.00	38.00-73.00	
**Ethnicity**			< 0.001
White	152 293 (95.4%)	141 272 (95.6%)	
Mixed-race	1 105 (0.7%)	661 (0.4%)	
Black	2 445 (1.5%)	1 812 (1.2%)	
Asian	2 016 (1.3%)	2 739 (1.9%)	
Chinese	505 (0.3%)	313 (0.2%)	
Other	1 210 (0.8%)	1 007 (0.7%)	
**Highest qualification**			< 0.001
None	19 497 (12.2%)	20 331 (13.8%)	
O levels/GCSEs/CSEs	47 467 (29.7%)	36 981 (25.0%)	
A levels/NVQ/HND/HNC[1]	36 545 (22.9%)	36 039 (24.4%)	
Degree	56 065 (35.1%)	54 453 (36.8%)	
**Household income**[2]			< 0.001
Very low	41 713 (26.1%)	40 625 (27.5%)	
Low	35 968 (22.5%)	27 131 (18.4%)	
Medium	42 060 (26.4%)	35 871 (24.3%)	
High	31 507 (19.7%)	34 599 (23.4%)	
Very high	8 326 (5.2%)	9 578 (6.5%)	
**Multiple deprivation**			< 0.001
Mean (SD)	16.65 (13.13)	16.90 (13.54)	
Range	0.61-82.00	0.61-82.00	
Psychosocial factors			
**Loneliness**			< 0.001
Not lonely	150 898 (94.6%)	139 003 (94.0%)	
Lonely	8 676 (5.4%)	8 801 (6.0%)	
**Social isolation**			< 0.001
Not isolated	146 366 (91.7%)	134 565 (91.0%)	
Isolated	13 208 (8.3%)	13 239 (9.0%)	
Lifestyle factors			
**Smoking status**			< 0.001
Never	94 928 (59.5%)	73 547 (49.8%)	
Former	51 194 (32.1%)	57 444 (38.9%)	
Current	13 452 (8.4%)	16 813 (11.4%)	
**Stair climbing frequency**			< 0.001
None	12 398 (7.8%)	11 651 (7.9%)	
1-5/day	28 452 (17.8%)	29 815 (20.2%)	
6-10/day	59 067 (37.0%)	56 915 (38.5%)	
11-15/day	32 437 (20.3%)	27 878 (18.9%)	
16-20/day	15 174 (9.5%)	12 435 (8.4%)	
20+/day	12 046 (7.5%)	9 110 (6.2%)	
**Alcohol intake frequency**			< 0.001
Never	41 206 (25.8%)	37 571 (25.4%)	
Special occasions	12 551 (7.9%)	7 872 (5.3%)	
1-3/month	21 911 (13.7%)	9 615 (6.5%)	
1-2/week	20 913 (13.1%)	12 885 (8.7%)	
3-4/week	35 098 (22.0%)	40 153 (27.2%)	
Daily/almost daily	27 895 (17.5%)	39 708 (26.9%)	
**Sleep duration** (hours/day)			< 0.001
Mean (SD)	7.18 (1.06)	7.13 (1.04)	
Range	1.00-20.00	1.00-20.00	
**BMI** (kg/m^2)			< 0.001
Mean (SD)	26.85 (5.07)	27.72 (4.13)	
Range	12.80-67.30	12.80-63.40	
**Walking** (days/week)[3]			< 0.001

Mean (SD)	5.43 (1.89)	5.29 (2.00)	
Range	0.00-7.00	0.00-7.00	
**Moderate activity** (days/week)[3]			0.352
Mean (SD)	3.59 (2.32)	3.58 (2.32)	
Range	0.00-7.00	0.00-7.00	
**Vigorous activity** (days/week)[3]			< 0.001
Mean (SD)	1.70 (1.84)	2.07 (2.02)	
Range	0.00-7.00	0.00-7.00	
Environmental exposures			
**$PM_{2.5}$**			0.005
Mean (SD)	9.96 (1.03)	9.95 (1.04)	
Range	8.17-19.89	8.17-21.25	
**PM_{10}**			0.159
Mean (SD)	16.19 (1.87)	16.18 (1.89)	
Range	11.78-30.65	11.78-30.65	
**NO_2**			< 0.001
Mean (SD)	26.49 (7.53)	26.38 (7.59)	
Range	12.93-107.47	12.93-108.49	
**L_{den}**			0.174
Mean (SD)	56.00 (4.21)	56.02 (4.27)	
Range	51.55-89.29	51.55-86.50	
**Greenspace** 1000m			< 0.001
Mean (SD)	45.27 (21.82)	45.78 (21.71)	
Range	4.49-99.18	4.54-99.19	

Note: GCSEs = general certificate of secondary education; CSE = certificate of secondary education; NVQ = national vocational qualification; HND = higher national diploma; HNC = higher national certificate; BMI = body mass index; PM = particulate matter; NO_2 = nitrogen dioxide; L_{den} = day-evening-night noise level. [1]also includes 'other professional qualifications'. [2]Annual household income groups: very low (<£18,000), low (£18,000–£30,999), middle (£31,000–£51,999), high (£52,000–£100,000) and very high (>£100,000). [3]number of days per week engaging in these activities for 10+ minutes continuously.

Table S26. Baseline characteristics stratified by age

Table S26. **Baseline characteristics stratified by age**

	Below 65 (*n* = 254 361)	65 and above (*n* = 53 017)	p value
Health indicators			
**Health status**			< 0.001
Unhealthy	71 234 (28.0%)	23 943 (45.2%)	
Healthy	183 127 (72.0%)	29 074 (54.8%)	
**Long-standing illness**			< 0.001
Yes	73 408 (28.9%)	20 349 (38.4%)	
No	180 953 (71.1%)	32 668 (61.6%)	
**Self-rated health**			< 0.001
Poor	9 382 (3.7%)	1 684 (3.2%)	
Fair	48 636 (19.1%)	10 533 (19.9%)	
Good	150 135 (59.0%)	32 564 (61.4%)	
Excellent	46 208 (18.2%)	8 236 (15.5%)	
Sociodemographic characteristics			
**Age**			< 0.001
Mean (SD)	53.86 (7.00)	66.87 (1.48)	
Range	38.00 - 64.00	65.00 - 73.00	
**Sex**			< 0.001
Female	135 416 (53.2%)	24 158 (45.6%)	
Male	118 945 (46.8%)	28 859 (54.4%)	
**Ethnicity**			< 0.001
White	241 835 (95.1%)	51 730 (97.6%)	
Mixed-race	1 641 (0.6%)	125 (0.2%)	
Black	3 943 (1.6%)	314 (0.6%)	
Asian	4 167 (1.6%)	588 (1.1%)	
Chinese	758 (0.3%)	60 (0.1%)	
Other	2 017 (0.8%)	200 (0.4%)	
**Highest qualification**			< 0.001
None	25 954 (10.2%)	13 874 (26.2%)	
O levels/GCSEs/CSEs	72 006 (28.3%)	12 442 (23.5%)	
A levels/NVQ/HND/HNC[1]	60 115 (23.6%)	12 469 (23.5%)	
Degree	96 286 (37.9%)	14 232 (26.8%)	
**Household income**[2]			< 0.001
Very low	72 706 (28.6%)	9 632 (18.2%)	
Low	42 889 (16.9%)	20 210 (38.1%)	
Medium	59 348 (23.3%)	18 583 (35.1%)	
High	62 369 (24.5%)	3 737 (7.0%)	
Very high	17 049 (6.7%)	855 (1.6%)	
**Multiple deprivation**			< 0.001
Mean (SD)	16.95 (13.44)	15.91 (12.78)	
Range	0.61 - 82.00	0.61 - 82.00	
Psychosocial factors			
**Loneliness**			< 0.001
Not lonely	239 515 (94.2%)	50 386 (95.0%)	
Lonely	14 846 (5.8%)	2 631 (5.0%)	
**Social isolation**			0.002
Not isolated	232 297 (91.3%)	48 634 (91.7%)	
Isolated	22 064 (8.7%)	4 383 (8.3%)	
Lifestyle factors			
**Smoking status**			< 0.001
Never	142 883 (56.2%)	25 592 (48.3%)	
Former	84 862 (33.4%)	23 776 (44.8%)	
Current	26 616 (10.5%)	3 649 (6.9%)	
**Stair climbing frequency**			< 0.001
None	17 222 (6.8%)	6 827 (12.9%)	
1-5/day	49 187 (19.3%)	9 080 (17.1%)	
6-10/day	96 576 (38.0%)	19 406 (36.6%)	
11-15/day	50 249 (19.8%)	10 066 (19.0%)	
16-20/day	23 039 (9.1%)	4 570 (8.6%)	
20+/day	18 088 (7.1%)	3 068 (5.8%)	
**Alcohol intake frequency**			< 0.001
Never	66 613 (26.2%)	12 164 (22.9%)	
Special occasions	16 139 (6.3%)	4 284 (8.1%)	
1-3/month	25 472 (10.0%)	6 054 (11.4%)	
1-2/week	28 784 (11.3%)	5 014 (9.5%)	
3-4/week	63 767 (25.1%)	11 484 (21.7%)	
Daily/almost daily	53 586 (21.1%)	14 017 (26.4%)	
**Sleep duration** (hours/day)			< 0.001
Mean (SD)	7.13 (1.04)	7.32 (1.11)	
Range	1.00 - 20.00	1.00 - 18.00	
**BMI** (kg/m^2)			< 0.001

Mean (SD)	27.24 (4.75)	27.40 (4.25)	
Range	12.80 - 67.30	12.80 - 56.50	
**Walking** (days/week)[3]			< 0.001
Mean (SD)	5.31 (1.97)	5.63 (1.78)	
Range	0.00 - 7.00	0.00 - 7.00	
**Moderate activity** (days/week)[3]			< 0.001
Mean (SD)	3.52 (2.31)	3.93 (2.32)	
Range	0.00 - 7.00	0.00 - 7.00	
**Vigorous activity** (days/week)[3]			< 0.001
Mean (SD)	1.89 (1.93)	1.80 (1.98)	
Range	0.00 - 7.00	0.00 - 7.00	
Environmental exposures			
**$PM_{2.5}$**			< 0.001
Mean (SD)	9.97 (1.04)	9.88 (1.01)	
Range	8.17 - 20.19	8.17 - 21.25	
**PM_{10}**			< 0.001
Mean (SD)	16.20 (1.88)	16.13 (1.85)	
Range	11.78 - 30.65	11.78 - 27.62	
**NO_2**			< 0.001
Mean (SD)	26.54 (7.59)	25.92 (7.40)	
Range	12.93 - 108.49	12.93 - 107.07	
**L_{den}**			< 0.001
Mean (SD)	56.03 (4.25)	55.92 (4.21)	
Range	51.55 - 89.29	51.55 - 86.28	
**Greenspace** 1000m			< 0.001
Mean (SD)	45.27 (21.77)	46.69 (21.72)	
Range	4.49 - 99.19	5.34 - 99.18	

Note: GCSEs = general certificate of secondary education; CSE = certificate of secondary education; NVQ = national vocational qualification; HND = higher national diploma; HNC = higher national certificate; BMI = body mass index; PM = particulate matter; NO_2 = nitrogen dioxide; L_{den} = day-evening-night noise level. [1]also includes 'other professional qualifications'. [2]Annual household income groups: very low (<£18,000), low (£18,000–£30,999), middle (£31,000–£51,999), high (£52,000–£100,000) and very high (>£100,000). [3]number of days per week engaging in these activities for 10+ minutes continuously.

Table S27-S30. Regression tables health status stratified by sex

Sociodemographic characteristics

Table S27. **Sociodemographic characteristics associated with health status stratified by sex**

	All participants			Male			Female			Interaction term	
Term	OR	Bonferroni-corrected CI		OR	Bonferroni-corrected CI		OR	Bonferroni-corrected CI		$p_{Bonf.}$	p_{BH}
**Household income**[1]											
Very low	0.7447	0.7148	0.7759	0.6953	0.6545	0.7386	0.7794	0.7368	0.8245	<0.0001	<0.0001
Low	0.9196	0.8864	0.9541	0.9052	0.8586	0.9544	0.9326	0.8858	0.9818	0.0006	0.0001
Middle	Ref	–	–	Ref	–	–	Ref	–	–	–	–
High	1.0510	1.0096	1.0941	1.0507	0.9934	1.1113	1.0438	0.9852	1.1060	0.4472	0.0225
Very high	1.0472	0.9812	1.1181	1.0541	0.9640	1.1535	1.0257	0.9327	1.1292	0.1482	0.0087
**Sex**											
Female	Ref	–	–	Ref	–	–	Ref	–	–	–	–
Male	0.8847	0.8610	0.9090	–	–	–	–	–	–	–	–
**Age**	0.9528	0.9510	0.9546	0.9441	0.9414	0.9467	0.9608	0.9583	0.9634	<0.0001	<0.0001
**Multiple deprivation**	0.9952	0.9941	0.9963	0.9943	0.9927	0.9959	0.9962	0.9946	0.9977	<0.0001	<0.0001
**Ethnicity**											
White	Ref	–	–	Ref	–	–	Ref	–	–	–	–
Mixed-race	1.1037	0.9221	1.3273	1.0296	0.7706	1.3907	1.1729	0.9339	1.4851	>0.9999	0.6739
Asian	1.0188	0.9149	1.1359	0.9194	0.7985	1.0605	1.1865	1.0024	1.4096	0.1043	0.0065
Black	1.3125	1.1657	1.4805	1.3079	1.0905	1.5754	1.3178	1.1274	1.5455	>0.9999	0.9911
Chinese	1.8339	1.3646	2.5106	1.7054	1.0800	2.8050	1.9827	1.3537	3.0032	>0.9999	0.8143
Other	1.2570	1.0727	1.4779	1.3268	1.0478	1.6919	1.2131	0.9805	1.5104	>0.9999	0.3444
**Highest qualification**											
None	Ref	–	–	Ref	–	–	Ref	–	–	–	–
O levels/GCSEs/CSEs	1.0315	0.9870	1.0779	0.9946	0.9330	1.0603	1.0482	0.9858	1.1146	0.0078	0.0006
A levels/NVQ/HND/HNC[2]	0.9879	0.9445	1.0332	0.9710	0.9114	1.0344	1.0033	0.9410	1.0697	0.0037	0.0004
Degree	0.9558	0.9137	0.9997	0.9400	0.8816	1.0021	0.9624	0.9029	1.0257	<0.0001	<0.0001

Note: Estimates from Model 4 (i.e. including all explanatory variables). Bonferroni-adjusted (~99.9%) confidence intervals. OR = odds ratio; CI = confidence interval; GCSEs = general certificate of secondary education; CSE = certificate of secondary education; NVQ = national vocational qualification; HND = higher national diploma; HNC = higher national certificate. [1]Annual household income groups: very low (<£18,000), low (£18,000–£30,999), middle (£31,000–£51,999), high (£52,000–£100,000) and very high (>£100,000). [2]also includes 'other professional qualifications'.

Psychosocial factors

Table S28. **Psychosocial factors associated with health status stratified by sex**

	All participants			Male			Female			Interaction term	
Term	OR	Bonferroni-corrected CI		OR	Bonferroni-corrected CI		OR	Bonferroni-corrected CI		$p_{Bonf.}$	p_{BH}
**Loneliness**											
Not lonely	Ref	–	–	Ref	–	–	Ref	–	–	–	–
Lonely	0.8129	0.7697	0.8587	0.7688	0.7112	0.8313	0.8538	0.7904	0.9227	0.0083	0.0006
**Social isolation**											
Not isolated	Ref	–	–	Ref	–	–	Ref	–	–	–	–
Isolated	0.9496	0.9067	0.9947	0.9307	0.8711	0.9946	0.9653	0.9044	1.0306	0.0818	0.0055

Note: Estimates from Model 4 (i.e. including all explanatory variables). Bonferroni-adjusted (~99.9%) confidence intervals. OR = odds ratio; CI = confidence interval.

Table S29. **Lifestyle factors associated with health status stratified by sex**

	All participants			Male			Female			Interaction term	
Term	OR	Bonferroni-corrected CI		OR	Bonferroni-corrected CI		OR	Bonferroni-corrected CI		$p_{Bonf.}$	p_{BH}
**Sleep duration** (hours/day)	0.9748	0.9631	0.9868	0.9635	0.9465	0.9808	0.9947	0.9782	1.0116	<0.0001	<0.0001
**Physical activity** (days/week)[1]											
Walking	1.0105	1.0032	1.0178	1.0062	0.9960	1.0164	1.0160	1.0055	1.0266	>0.9999	0.1722
Moderate activity	1.0032	0.9964	1.0100	1.0093	0.9992	1.0195	0.9979	0.9887	1.0071	0.8784	0.0351
Vigorous activity	1.0319	1.0238	1.0400	1.0319	1.0205	1.0433	1.0279	1.0164	1.0395	0.0064	0.0006
**Stair climbing frequency**											
None	Ref	–	–	Ref	–	–	Ref	–	–	–	–
1-5/day	1.0704	1.0148	1.1289	1.0604	0.9827	1.1442	1.0778	0.9998	1.1617	>0.9999	0.1995
6-10/day	1.1756	1.1188	1.2352	1.1657	1.0854	1.2517	1.1836	1.1044	1.2683	0.5750	0.0261
11-15/day	1.1714	1.1098	1.2363	1.1553	1.0684	1.2491	1.1898	1.1038	1.2823	0.6537	0.0284
16-20/day	1.1650	1.0937	1.2410	1.1425	1.0422	1.2525	1.1971	1.0971	1.3063	>0.9999	0.1028
20+/day	1.1881	1.1097	1.2723	1.2364	1.1172	1.3688	1.1660	1.0627	1.2797	0.0004	<0.0001
**Alcohol intake frequency**											
Never	0.6285	0.5946	0.6644	0.6097	0.5586	0.6656	0.6279	0.5842	0.6749	0.7799	0.0325
Special occasions	0.7792	0.7429	0.8174	0.7591	0.7003	0.8231	0.7720	0.7270	0.8199	>0.9999	0.0621
1-3/month	0.9129	0.8708	0.9570	0.8829	0.8204	0.9503	0.9244	0.8689	0.9836	>0.9999	0.1105
1-2/week	Ref	–	–	Ref	–	–	Ref	–	–	–	–
3-4/week	1.0590	1.0198	1.0998	1.0837	1.0280	1.1424	1.0342	0.9794	1.0922	0.4505	0.0225
Daily/almost daily	1.0334	0.9939	1.0745	1.0604	1.0054	1.1183	0.9982	0.9419	1.0579	>0.9999	0.0392
**BMI** (kg/m^2)	0.9683	0.9656	0.9711	0.9564	0.9520	0.9608	0.9746	0.9711	0.9782	<0.0001	<0.0001
**Smoking status**											
Never	Ref	–	–	Ref	–	–	Ref	–	–	–	–
Former	0.7922	0.7698	0.8152	0.7694	0.7384	0.8018	0.8366	0.8036	0.8711	<0.0001	<0.0001
Current	0.7518	0.7183	0.7869	0.7812	0.7337	0.8321	0.7188	0.6725	0.7685	0.0747	0.0053

Note: Estimates from Model 4 (i.e. including all explanatory variables). Bonferroni-adjusted (~99.9%) confidence intervals. OR = odds ratio; CI = confidence interval; BMI = body mass index. [1]number of days per week engaging in these activities for 10+ minutes continuously.

Environmental exposures

Table S30. **Environmental exposures associated with health status stratified by sex**

	All participants			Male			Female			Interaction term	
Term	OR	Bonferroni-corrected CI		OR	Bonferroni-corrected CI		OR	Bonferroni-corrected CI		$p_{Bonf.}$	p_{BH}
**$PM_{2.5}$**	0.9656	0.9411	0.9908	0.9614	0.9266	0.9976	0.9690	0.9347	1.0045	0.1946	0.0108
**PM_{10}**	1.0019	0.9935	1.0104	1.0012	0.9892	1.0134	1.0029	0.9911	1.0148	>0.9999	0.1722
**NO_2**	1.0036	0.9993	1.0078	1.0010	0.9949	1.0071	1.0060	1.0001	1.0120	0.4953	0.0236
**L_{den}**	0.9981	0.9945	1.0017	1.0009	0.9957	1.0061	0.9955	0.9905	1.0005	>0.9999	0.5176
**Greenspace** 1000m	0.9995	0.9985	1.0004	0.9986	0.9973	1.0000	1.0003	0.9989	1.0017	>0.9999	0.7593

Note: Estimates from Model 4 (i.e. including all explanatory variables). Bonferroni-adjusted (~99.9%) confidence intervals. OR = odds ratio; CI = confidence interval; PM = particulate matter; NO_2 = nitrogen dioxide; L_{den} = day-evening-night noise level.

Figures S7-S9. Confidence interval plots health status stratified by sex

Sociodemographic and psychosocial factors

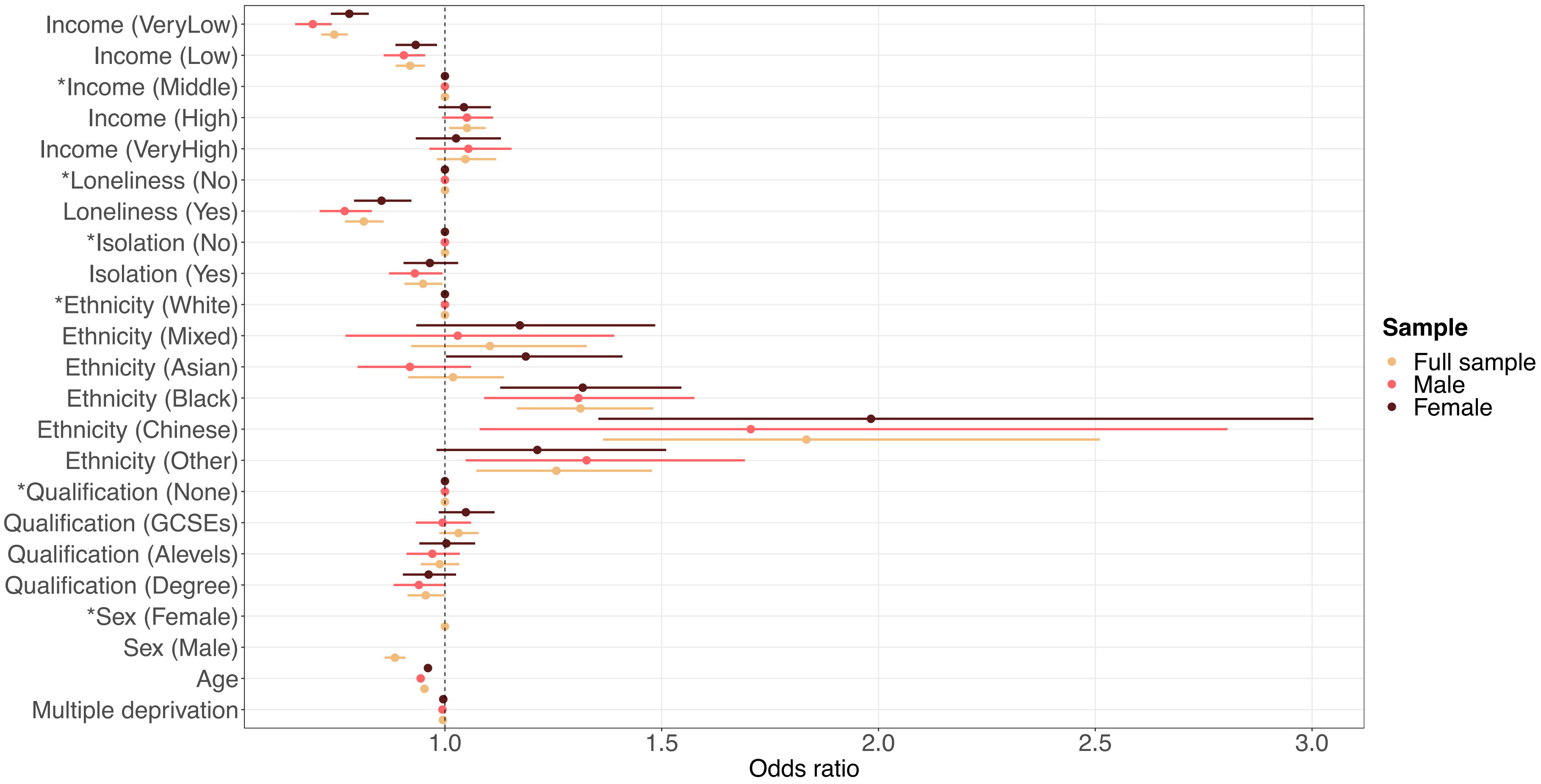

Figure S7. Sociodemographic characteristics and psychosocial factors associated with health status, stratified by sex. Confidence interval plot (odds ratio ± Bonferroni-adjusted (~99.9%) confidence intervals) for Model 4 (i.e. including all explanatory variables). GCSEs = general certificate of secondary education. *Indicates reference group for categorical explanatory variables. Annual household income groups: very low (<£18,000), low (£18,000–£30,999), middle (£31,000–£51,999), high (£52,000–£100,000) and very high (>£100,000). 'GCSEs' also includes O levels and certificate of secondary education (CSE). 'A levels' also includes national vocational qualification (NVQ), higher national diploma (HND), higher national certificate (HNC) and 'other professional qualifications'.

Lifestyle factors

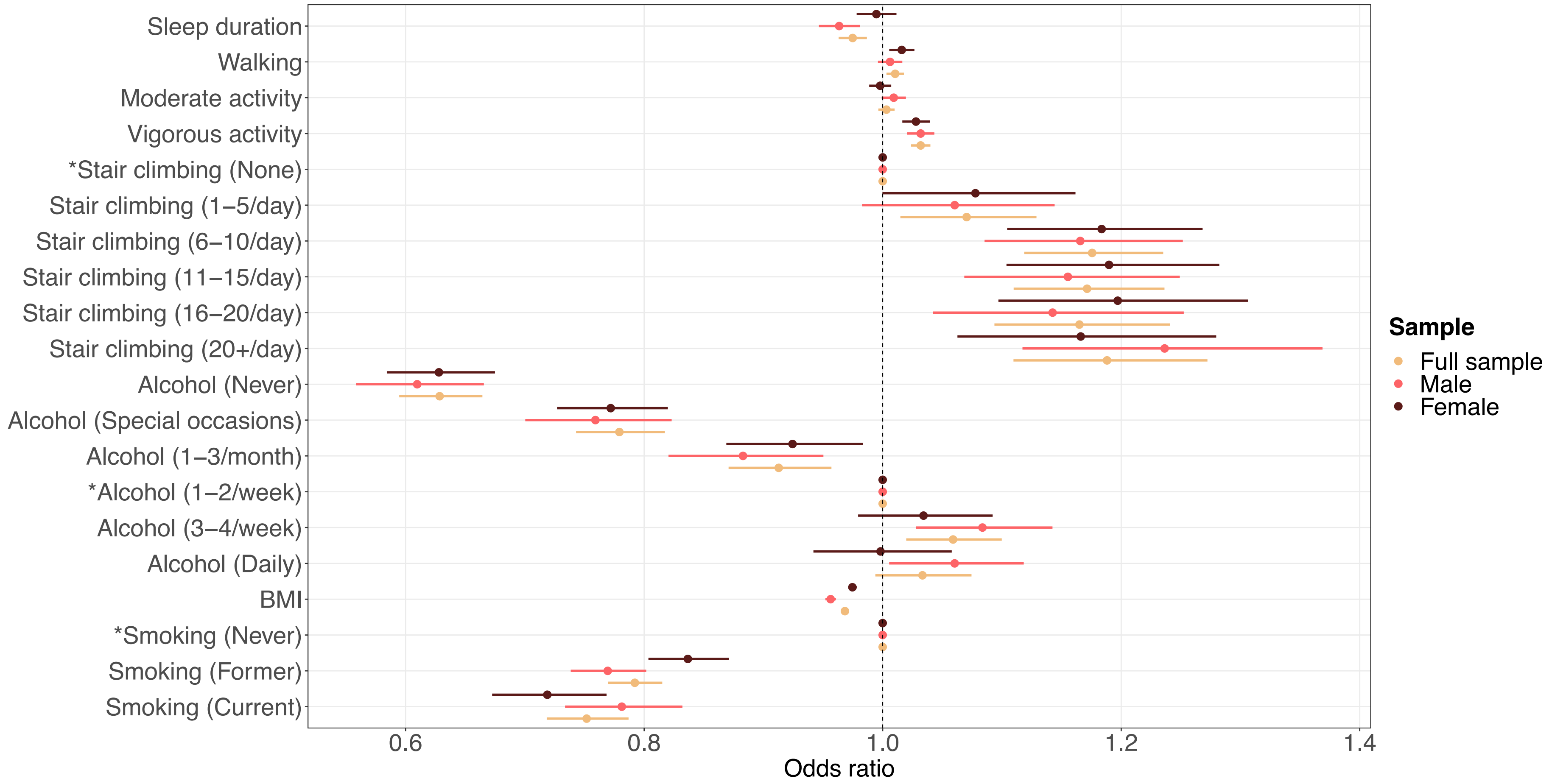

Figure S8. Lifestyle factors associated with health status, stratified by sex. Confidence interval plot (odds ratio ± Bonferroni-adjusted (~99.9%) confidence intervals) for Model 4 (i.e. including all explanatory variables). BMI = body mass index. *Indicates reference group for categorical explanatory variables.

Environmental exposures

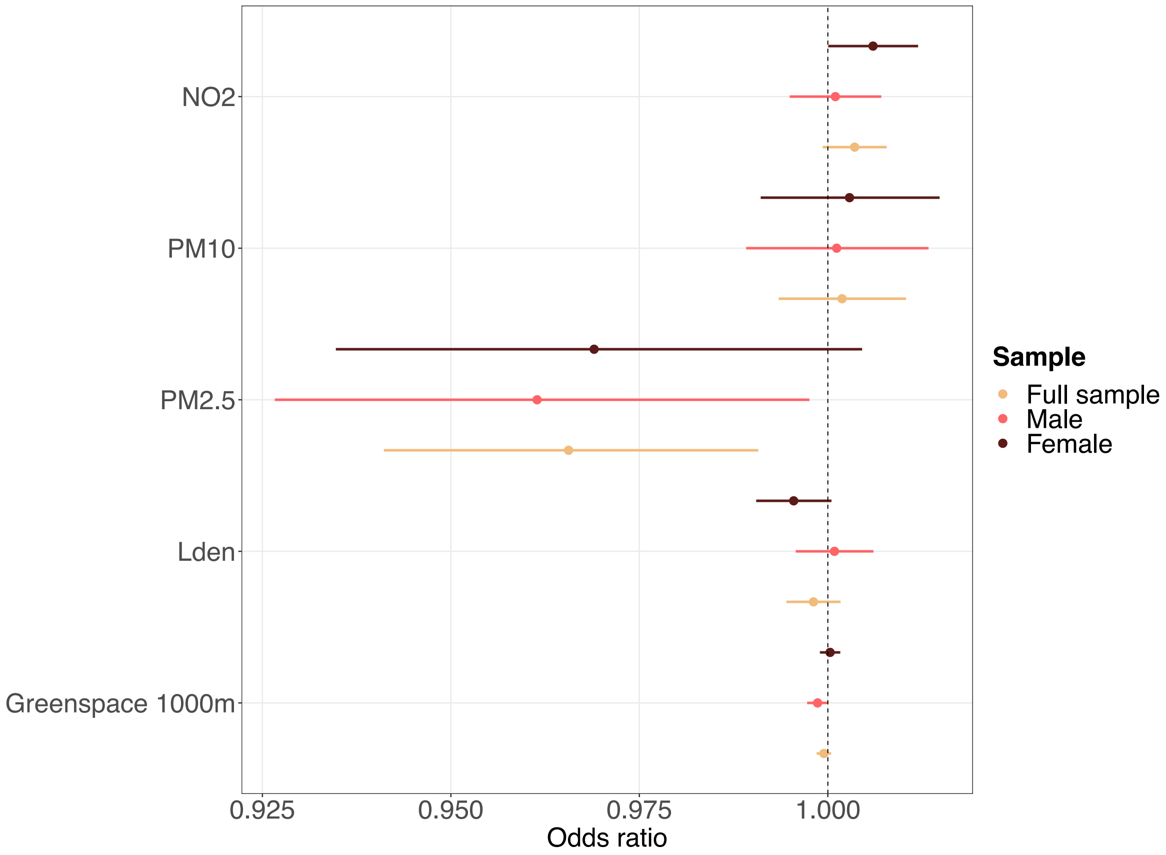

Figure S9. Environmental exposures associated with health status, stratified by sex. Confidence interval plot (odds ratio ± Bonferroni-adjusted (~99.9%) confidence intervals) for Model 4 (i.e. including all explanatory variables). PM = particulate matter; NO_2 = nitrogen dioxide; L_{den} = day-evening-night noise level.

Tables S31-S34. Regression tables health status stratified by age

Sociodemographic characteristics

Table S31. **Sociodemographic characteristics associated with health status stratified by age**

	All participants			Below 65			65 and above			Interaction term	
Term	OR	Bonferroni-corrected CI		OR	Bonferroni-corrected CI		OR	Bonferroni-corrected CI		$p_{Bonf.}$	p_{BH}
**Household income**[1]											
Very low	0.7447	0.7148	0.7759	0.7132	0.6807	0.7471	0.8509	0.7764	0.9326	<0.0001	<0.0001
Low	0.9196	0.8864	0.9541	0.9249	0.8875	0.9639	0.9432	0.8666	1.0264	>0.9999	0.7976
Middle	Ref	–	–	Ref	–	–	Ref	–	–	–	–
High	1.0510	1.0096	1.0941	1.0509	1.0070	1.0967	0.9563	0.8415	1.0872	0.2171	0.0155
Very high	1.0472	0.9812	1.1181	1.0428	0.9741	1.1169	0.9791	0.7734	1.2424	>0.9999	0.2886
**Sex**											
Female	Ref	–	–	Ref	–	–	Ref	–	–	–	–
Male	0.8847	0.8610	0.9090	0.9270	0.8992	0.9557	0.7548	0.7101	0.8023	<0.0001	<0.0001
**Age**	0.9528	0.9510	0.9546	0.9553	0.9530	0.9575	0.9436	0.9255	0.9620	–	–
**Multiple deprivation**	0.9952	0.9941	0.9963	0.9951	0.9939	0.9963	0.9958	0.9933	0.9984	>0.9999	0.1995
**Ethnicity**											
White	Ref	–	–	Ref	–	–	Ref	–	–	–	–
Mixed-race	1.1037	0.9221	1.3273	1.1227	0.9296	1.3634	1.0573	0.5866	1.9315	>0.9999	0.6005
Asian	1.0188	0.9149	1.1359	1.0654	0.9472	1.2003	0.8668	0.6575	1.1426	<0.0001	<0.0001
Black	1.3125	1.1657	1.4805	1.3410	1.1832	1.5233	1.2654	0.8693	1.8516	>0.9999	0.9840
Chinese	1.8339	1.3646	2.5106	1.8577	1.3604	2.5919	1.9177	0.7868	5.2841	>0.9999	0.7976
Other	1.2570	1.0727	1.4779	1.2936	1.0925	1.5381	1.1064	0.6937	1.7755	>0.9999	0.0544
**Highest qualification**											
None	Ref	–	–	Ref	–	–	Ref	–	–	–	–
O levels/GCSEs/CSEs	1.0315	0.9870	1.0779	1.0315	0.9786	1.0873	1.0540	0.9688	1.1466	>0.9999	0.1995
A levels/NVQ/HND/HNC[2]	0.9879	0.9445	1.0332	0.9907	0.9388	1.0454	1.0009	0.9197	1.0892	0.8449	0.0422
Degree	0.9558	0.9137	0.9997	0.9517	0.9022	1.0039	1.0103	0.9246	1.1040	0.8264	0.0422

Note: Estimates from Model 4 (i.e. including all explanatory variables). Bonferroni-adjusted (~99.9%) confidence intervals. OR = odds ratio; CI = confidence interval; GCSEs = general certificate of secondary education; CSE = certificate of secondary education; NVQ = national vocational qualification; HND = higher national diploma; HNC = higher national certificate. [1]Annual household income groups: very low (<£18,000), low (£18,000–£30,999), middle (£31,000–£51,999), high (£52,000–£100,000) and very high (>£100,000). [2]also includes 'other professional qualifications'.

Psychosocial factors

Table S32. **Psychosocial factors associated with health status stratified by age**

	All participants			Below 65			65 and above			Interaction term	
Term	OR	Bonferroni-corrected CI		OR	Bonferroni-corrected CI		OR	Bonferroni-corrected CI		$p_{Bonf.}$	p_{BH}
**Loneliness**											
Not lonely	Ref	–	–	Ref	–	–	Ref	–	–	–	–
Lonely	0.8129	0.7697	0.8587	0.7959	0.7497	0.8452	0.8958	0.7844	1.0231	0.3908	0.0244
**Social isolation**											
Not isolated	Ref	–	–	Ref	–	–	Ref	–	–	–	–
Isolated	0.9496	0.9067	0.9947	0.9443	0.8970	0.9942	0.9625	0.8650	1.0711	0.0112	0.0009

Note: Estimates from Model 4 (i.e. including all explanatory variables). Bonferroni-adjusted (~99.9%) confidence intervals. OR = odds ratio; CI = confidence interval.

Table S33. **Lifestyle factors associated with health status stratified by age**

	All participants			Below 65			65 and above			Interaction term	
Term	OR	Bonferroni-corrected CI		OR	Bonferroni-corrected CI		OR	Bonferroni-corrected CI		$p_{Bonf.}$	p_{BH}
**Sleep duration** (hours/day)	0.9748	0.9631	0.9868	0.9784	0.9650	0.9920	0.9787	0.9537	1.0043	>0.9999	0.1995
**Physical activity** (days/week)[1]											
Walking	1.0105	1.0032	1.0178	1.0084	1.0003	1.0165	1.0242	1.0067	1.0421	<0.0001	<0.0001
Moderate activity	1.0032	0.9964	1.0100	1.0026	0.9949	1.0103	1.0057	0.9913	1.0204	>0.9999	0.1763
Vigorous activity	1.0319	1.0238	1.0400	1.0338	1.0246	1.0432	1.0241	1.0075	1.0410	>0.9999	0.1536
**Stair climbing frequency**											
None	Ref	–	–	Ref	–	–	Ref	–	–	–	–
1-5/day	1.0704	1.0148	1.1289	1.1064	1.0395	1.1774	0.9614	0.8649	1.0686	<0.0001	<0.0001
6-10/day	1.1756	1.1188	1.2352	1.2041	1.1355	1.2766	1.1106	1.0115	1.2194	<0.0001	<0.0001
11-15/day	1.1714	1.1098	1.2363	1.1985	1.1249	1.2767	1.1195	1.0079	1.2434	0.0004	<0.0001
16-20/day	1.1650	1.0937	1.2410	1.1772	1.0942	1.2665	1.1735	1.0324	1.3343	0.4709	0.0277
20+/day	1.1881	1.1097	1.2723	1.2150	1.1236	1.3139	1.1410	0.9861	1.3210	0.0128	0.0010
**Alcohol intake frequency**											
Never	0.6285	0.5946	0.6644	0.6145	0.5772	0.6543	0.6783	0.6023	0.7636	0.0021	0.0002
Special occasions	0.7792	0.7429	0.8174	0.7720	0.7317	0.8146	0.8078	0.7273	0.8972	0.0052	0.0005
1-3/month	0.9129	0.8708	0.9570	0.9015	0.8557	0.9499	0.9684	0.8669	1.0820	>0.9999	0.0775
1-2/week	Ref	–	–	Ref	–	–	Ref	–	–	–	–
3-4/week	1.0590	1.0198	1.0998	1.0552	1.0117	1.1006	1.0730	0.9841	1.1698	>0.9999	0.2894
Daily/almost daily	1.0334	0.9939	1.0745	1.0310	0.9864	1.0776	1.0607	0.9751	1.1538	>0.9999	0.1995
**BMI** (kg/m^2)	0.9683	0.9656	0.9711	0.9694	0.9663	0.9724	0.9625	0.9557	0.9693	<0.0001	<0.0001
**Smoking status**											
Never	Ref	–	–	Ref	–	–	Ref	–	–	–	–
Former	0.7922	0.7698	0.8152	0.8054	0.7796	0.8320	0.7632	0.7175	0.8117	<0.0001	<0.0001
Current	0.7518	0.7183	0.7869	0.7423	0.7066	0.7799	0.8400	0.7460	0.9459	0.6816	0.0379

Note: Estimates from Model 4 (i.e. including all explanatory variables). Bonferroni-adjusted (~99.9%) confidence intervals. OR = odds ratio; CI = confidence interval; BMI = body mass index. [1]number of days per week engaging in these activities for 10+ minutes continuously.

Table S34. **Environmental exposures associated with health status stratified by age**

	All participants			Below 65			65 and above			Interaction term	
Term	OR	Bonferroni-corrected CI		OR	Bonferroni-corrected CI		OR	Bonferroni-corrected CI		$p_{Bonf.}$	p_{BH}
**$PM_{2.5}$**	0.9656	0.9411	0.9908	0.9641	0.9367	0.9922	0.9640	0.9099	1.0213	>0.9999	0.2963
**PM_{10}**	1.0019	0.9935	1.0104	1.0006	0.9912	1.0101	1.0064	0.9876	1.0255	>0.9999	0.2092
**NO_2**	1.0036	0.9993	1.0078	1.0037	0.9990	1.0085	1.0043	0.9949	1.0138	>0.9999	0.8340
**L_{den}**	0.9981	0.9945	1.0017	0.9990	0.9950	1.0030	0.9946	0.9866	1.0026	0.3084	0.0206
**Greenspace** 1000m	0.9995	0.9985	1.0004	0.9995	0.9984	1.0006	0.9994	0.9972	1.0015	>0.9999	0.2912

Note: Estimates from Model 4 (i.e. including all explanatory variables). Bonferroni-adjusted (~99.9%) confidence intervals. OR = odds ratio; CI = confidence interval; PM = particulate matter; NO_2 = nitrogen dioxide; L_{den} = day-evening-night noise level.

Figures S10-S12. Confidence interval plots health status stratified by age

Sociodemographic and psychosocial factors

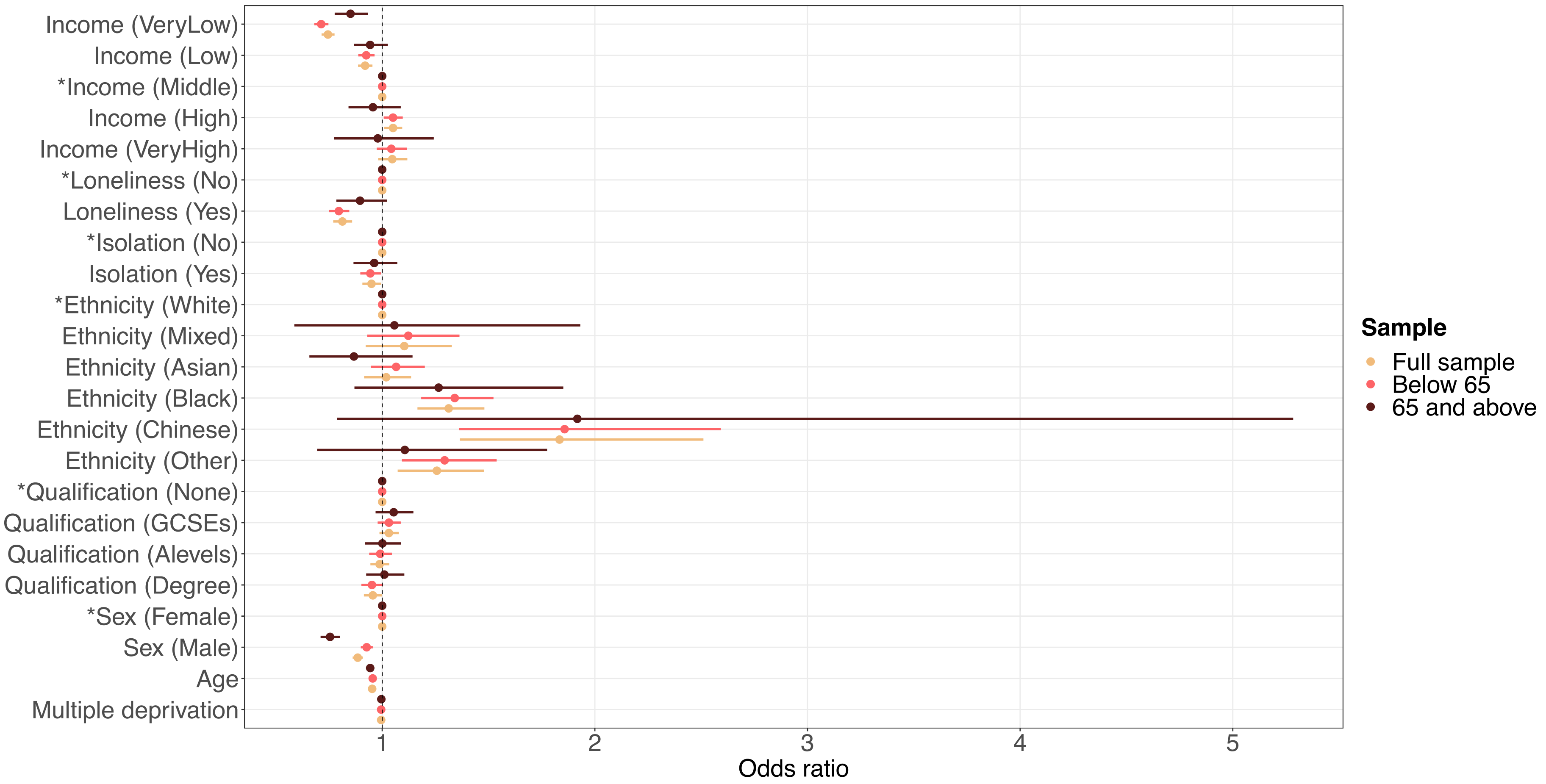

Figure S10. Sociodemographic characteristics and psychosocial factors associated with health status, stratified by age. Confidence interval plot (odds ratio ± Bonferroni-adjusted (~99.9%) confidence intervals) for Model 4 (i.e. including all explanatory variables). GCSEs = general certificate of secondary education. *Indicates reference group for categorical explanatory variables. Annual household income groups: very low (<£18,000), low (£18,000–£30,999), middle (£31,000–£51,999), high (£52,000–£100,000) and very high (>£100,000). 'GCSEs' also includes O levels and certificate of secondary education (CSE). 'A levels' also includes national vocational qualification (NVQ), higher national diploma (HND), higher national certificate (HNC) and 'other professional qualifications'.

Lifestyle factors

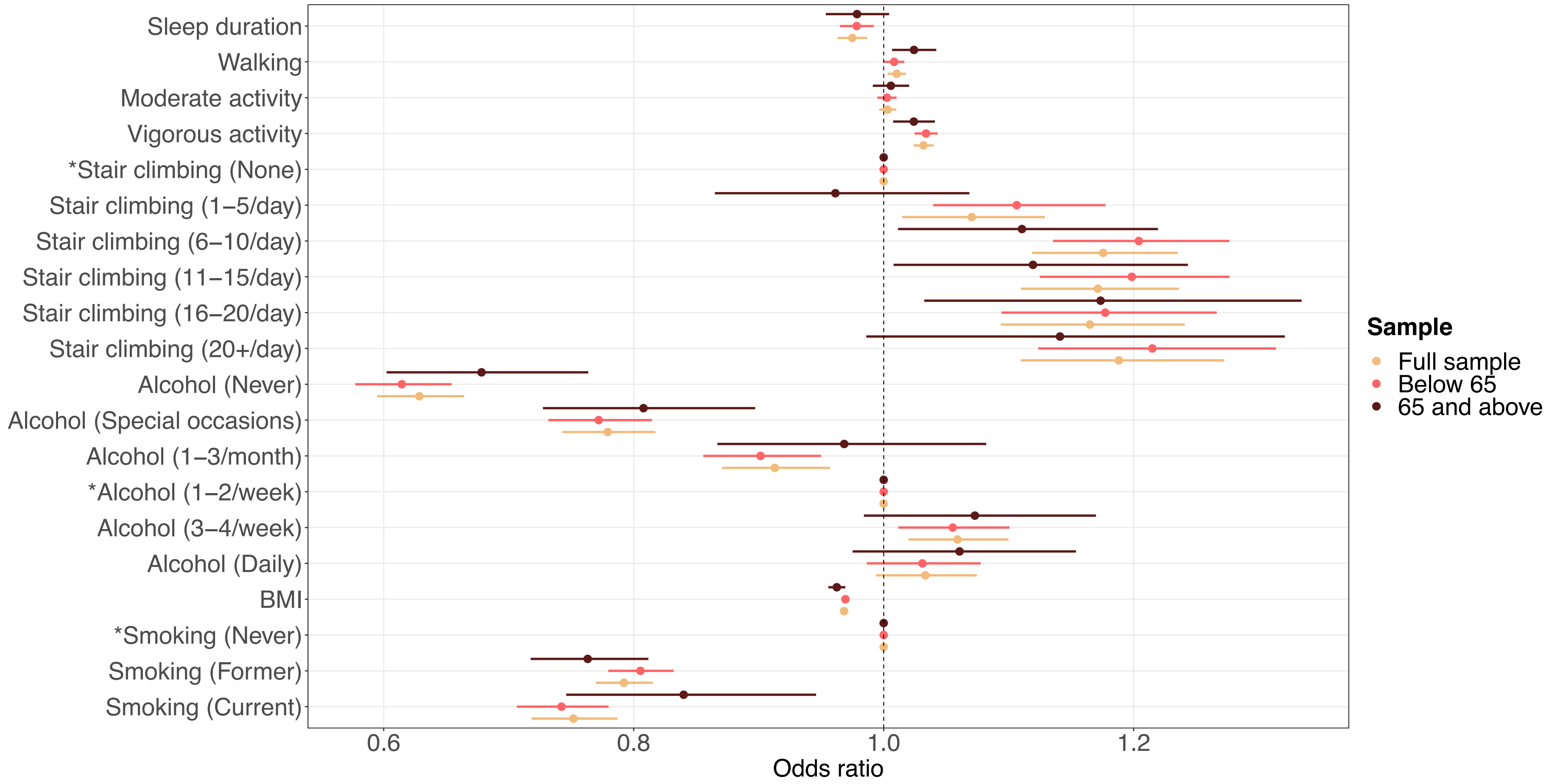

Figure S11. Lifestyle factors associated with health status, stratified by age. Confidence interval plot (odds ratio ± Bonferroni-adjusted (~99.9%) confidence intervals) for Model 4 (i.e. including all explanatory variables). BMI = body mass index. *Indicates reference group for categorical explanatory variables.

Environmental exposures

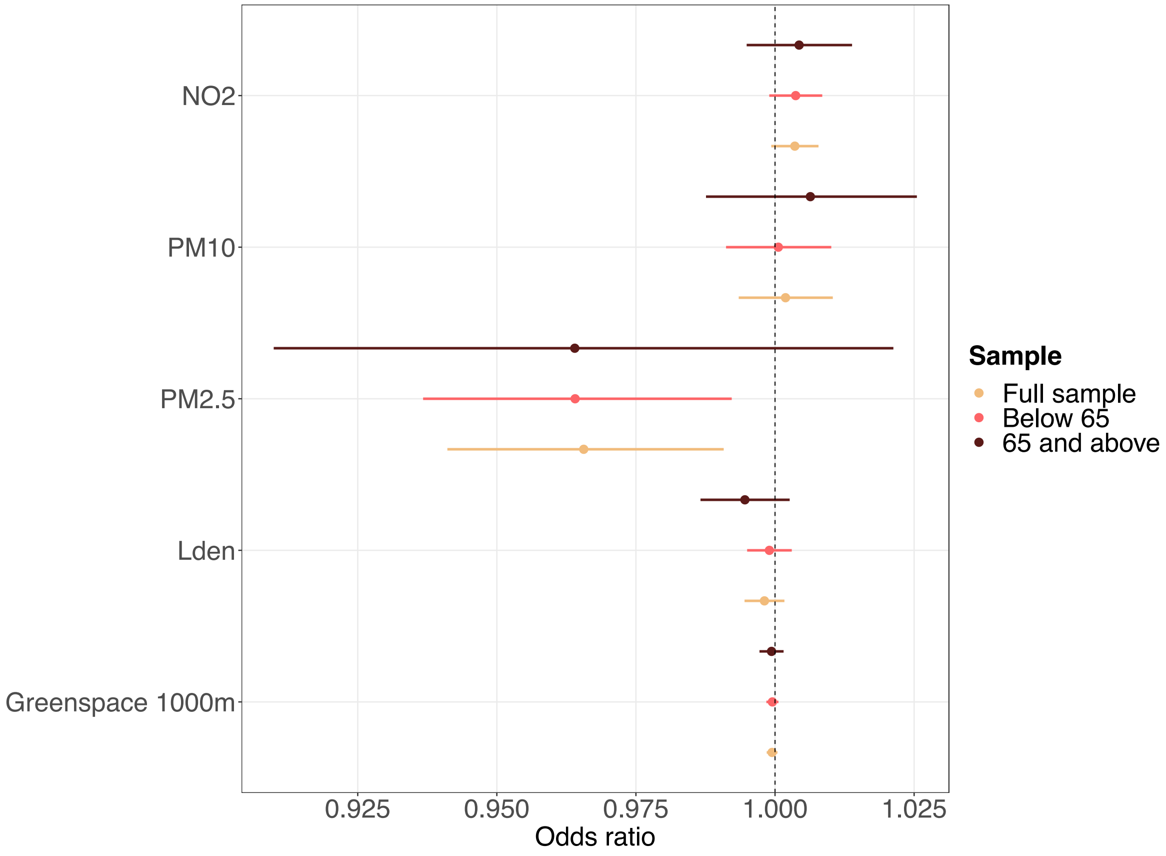

Figure S12. Environmental exposures associated with health status, stratified by age. Confidence interval plot (odds ratio ± Bonferroni-adjusted (~99.9%) confidence intervals) for Model 4 (i.e. including all explanatory variables). PM = particulate matter; NO_2 = nitrogen dioxide; L_{den} = day-evening-night noise level.

Tables S35-S38. Regression tables long-standing illness stratified by sex

Sociodemographic characteristics

Table S35. **Sociodemographic characteristics associated with long-standing illness stratified by sex**

	All participants			Male			Female			Interaction term	
Term	OR	Bonferroni-corrected CI		OR	Bonferroni-corrected CI		OR	Bonferroni-corrected CI		$p_{Bonf.}$	p_{BH}
**Household income**[1]											
Very low	0.6654	0.6384	0.6934	0.6067	0.5714	0.6442	0.7141	0.6743	0.7563	<0.0001	<0.0001
Low	0.8906	0.8581	0.9244	0.8593	0.8154	0.9056	0.9228	0.8752	0.9729	0.0001	<0.0001
Middle	Ref	–	–	Ref	–	–	Ref	–	–	–	–
High	1.1171	1.0726	1.1634	1.1170	1.0568	1.1807	1.1068	1.0426	1.1752	>0.9999	0.1703
Very high	1.2524	1.1703	1.3410	1.2639	1.1547	1.3847	1.2112	1.0934	1.3436	0.9984	0.0605
**Sex**											
Female	Ref	–	–	Ref	–	–	Ref	–	–	–	–
Male	0.7585	0.7380	0.7797	–	–	–	–	–	–	–	–
**Age**	0.9730	0.9711	0.9748	0.9684	0.9658	0.9710	0.9775	0.9748	0.9801	<0.0001	<0.0001
**Multiple deprivation**	0.9921	0.9910	0.9932	0.9919	0.9903	0.9934	0.9924	0.9908	0.9940	0.3315	0.0255
**Ethnicity**											
White	Ref	–	–	Ref	–	–	Ref	–	–	–	–
Mixed-race	1.1223	0.9387	1.3474	1.1439	0.8594	1.5376	1.1235	0.8952	1.4203	>0.9999	0.5334
Asian	1.2225	1.0962	1.3652	1.1664	1.0117	1.3474	1.2903	1.0886	1.5350	>0.9999	0.9754
Black	1.1852	1.0591	1.3277	1.4492	1.2161	1.7330	1.0314	0.8905	1.1966	<0.0001	<0.0001
Chinese	1.6205	1.2207	2.1842	1.5633	1.0145	2.4879	1.7049	1.1788	2.5373	>0.9999	0.9159
Other	1.2027	1.0301	1.4078	1.2967	1.0326	1.6367	1.1288	0.9151	1.3993	>0.9999	0.0605
**Highest qualification**											
None	Ref	–	–	Ref	–	–	Ref	–	–	–	–
O levels/GCSEs/CSEs	0.9920	0.9485	1.0375	0.9806	0.9199	1.0453	0.9820	0.9212	1.0466	0.0149	0.0014
A levels/NVQ/HND/HNC[2]	0.8953	0.8554	0.9371	0.9294	0.8723	0.9902	0.8574	0.8023	0.9161	<0.0001	<0.0001
Degree	0.8550	0.8167	0.8950	0.9002	0.8442	0.9599	0.8028	0.7514	0.8576	<0.0001	<0.0001

Note: Estimates from Model 4 (i.e. including all explanatory variables). Bonferroni-adjusted (~99.9%) confidence intervals. OR = odds ratio; CI = confidence interval; GCSEs = general certificate of secondary education; CSE = certificate of secondary education; NVQ = national vocational qualification; HND = higher national diploma; HNC = higher national certificate. [1]Annual household income groups: very low (<£18,000), low (£18,000–£30,999), middle (£31,000–£51,999), high (£52,000–£100,000) and very high (>£100,000). [2]also includes 'other professional qualifications'.

Psychosocial factors

Table S36. **Psychosocial factors associated with long-standing illness stratified by sex**										
	All participants			Male			Female			Interaction term	
Term	OR	Bonferroni-corrected CI		OR	Bonferroni-corrected CI		OR	Bonferroni-corrected CI		$p_{Bonf.}$	p_{BH}
**Loneliness**											
Not lonely	Ref	–	–	Ref	–	–	Ref	–	–	–	–
Lonely	0.7097	0.6722	0.7494	0.6682	0.6191	0.7214	0.7526	0.6964	0.8136	0.0037	0.0005
**Social isolation**											
Not isolated	Ref	–	–	Ref	–	–	Ref	–	–	–	–
Isolated	0.9185	0.8770	0.9621	0.8990	0.8422	0.9599	0.9325	0.8731	0.9963	>0.9999	0.0605

Note: Estimates from Model 4 (i.e. including all explanatory variables). Bonferroni-adjusted (~99.9%) confidence intervals. OR = odds ratio; CI = confidence interval.

Table S37. **Lifestyle factors associated with long-standing illness stratified by sex**

	All participants			Male			Female			Interaction term	
Term	OR	Bonferroni-corrected CI		OR	Bonferroni-corrected CI		OR	Bonferroni-corrected CI		$p_{Bonf.}$	p_{BH}
**Sleep duration** (hours/day)	1.0004	0.9882	1.0128	1.0000	0.9825	1.0179	1.0087	0.9915	1.0262	>0.9999	0.0605
**Physical activity** (days/week)[1]											
Walking	1.0250	1.0175	1.0325	1.0173	1.0071	1.0275	1.0342	1.0233	1.0453	0.0177	0.0015
Moderate activity	0.9984	0.9916	1.0053	1.0097	0.9997	1.0199	0.9893	0.9799	0.9987	0.3576	0.0255
Vigorous activity	1.0641	1.0557	1.0727	1.0618	1.0502	1.0736	1.0636	1.0512	1.0761	>0.9999	0.0721
**Stair climbing frequency**											
None	Ref	–	–	Ref	–	–	Ref	–	–	–	–
1-5/day	1.1101	1.0522	1.1710	1.0747	0.9963	1.1592	1.1463	1.0625	1.2366	>0.9999	0.9829
6-10/day	1.2937	1.2307	1.3598	1.2203	1.1365	1.3100	1.3692	1.2762	1.4688	>0.9999	0.2991
11-15/day	1.3131	1.2434	1.3866	1.2388	1.1459	1.3392	1.3897	1.2873	1.5001	>0.9999	0.4917
16-20/day	1.3066	1.2255	1.3932	1.2230	1.1158	1.3406	1.3984	1.2784	1.5300	>0.9999	0.3744
20+/day	1.2975	1.2108	1.3907	1.2701	1.1483	1.4053	1.3355	1.2141	1.4695	>0.9999	0.3002
**Alcohol intake frequency**											
Never	0.5533	0.5234	0.5850	0.5987	0.5488	0.6532	0.5192	0.4827	0.5584	0.0152	0.0014
Special occasions	0.7142	0.6809	0.7492	0.7400	0.6833	0.8016	0.6913	0.6506	0.7347	>0.9999	0.2105
1-3/month	0.8486	0.8096	0.8895	0.8393	0.7811	0.9020	0.8477	0.7962	0.9027	>0.9999	0.9159
1-2/week	Ref	–	–	Ref	–	–	Ref	–	–	–	–
3-4/week	1.0925	1.0514	1.1353	1.1021	1.0459	1.1612	1.0875	1.0274	1.1512	>0.9999	0.3744
Daily/almost daily	1.0744	1.0326	1.1180	1.0780	1.0224	1.1366	1.0766	1.0129	1.1444	>0.9999	0.9331
**BMI** (kg/m^2)	0.9419	0.9392	0.9446	0.9390	0.9347	0.9434	0.9437	0.9402	0.9472	0.0071	0.0008
**Smoking status**											
Never	Ref	–	–	Ref	–	–	Ref	–	–	–	–
Former	0.7978	0.7749	0.8213	0.7711	0.7401	0.8034	0.8382	0.8040	0.8739	<0.0001	<0.0001
Current	0.7858	0.7506	0.8227	0.8126	0.7637	0.8648	0.7519	0.7024	0.8051	>0.9999	0.1703

Note: Estimates from Model 4 (i.e. including all explanatory variables). Bonferroni-adjusted (~99.9%) confidence intervals. OR = odds ratio; CI = confidence interval; BMI = body mass index. [1]number of days per week engaging in these activities for 10+ minutes continuously.

Environmental exposures

Table S38. **Environmental exposures associated with long-standing illness stratified by sex**

	All participants			Male			Female			Interaction term	
Term	OR	Bonferroni-corrected CI		OR	Bonferroni-corrected CI		OR	Bonferroni-corrected CI		$p_{Bonf.}$	p_{BH}
**$PM_{2.5}$**	0.9677	0.9430	0.9932	0.9629	0.9283	0.9987	0.9713	0.9362	1.0079	>0.9999	0.0721
**PM_{10}**	1.0019	0.9934	1.0105	1.0004	0.9884	1.0125	1.0039	0.9917	1.0162	>0.9999	0.3238
**NO_2**	1.0025	0.9982	1.0068	1.0011	0.9951	1.0071	1.0042	0.9981	1.0104	>0.9999	0.3424
**L_{den}**	1.0015	0.9979	1.0052	1.0037	0.9986	1.0089	0.9994	0.9942	1.0046	>0.9999	0.4917
**Greenspace** 1000m	0.9997	0.9987	1.0007	0.9992	0.9978	1.0006	1.0001	0.9988	1.0015	>0.9999	0.9754

Note: Estimates from Model 4 (i.e. including all explanatory variables). Bonferroni-adjusted (~99.9%) confidence intervals. OR = odds ratio; CI = confidence interval; PM = particulate matter; NO_2 = nitrogen dioxide; L_{den} = day-evening-night noise level.

Figures S13-S15. Confidence interval plots long-standing illness stratified by sex

Sociodemographic and psychosocial factors

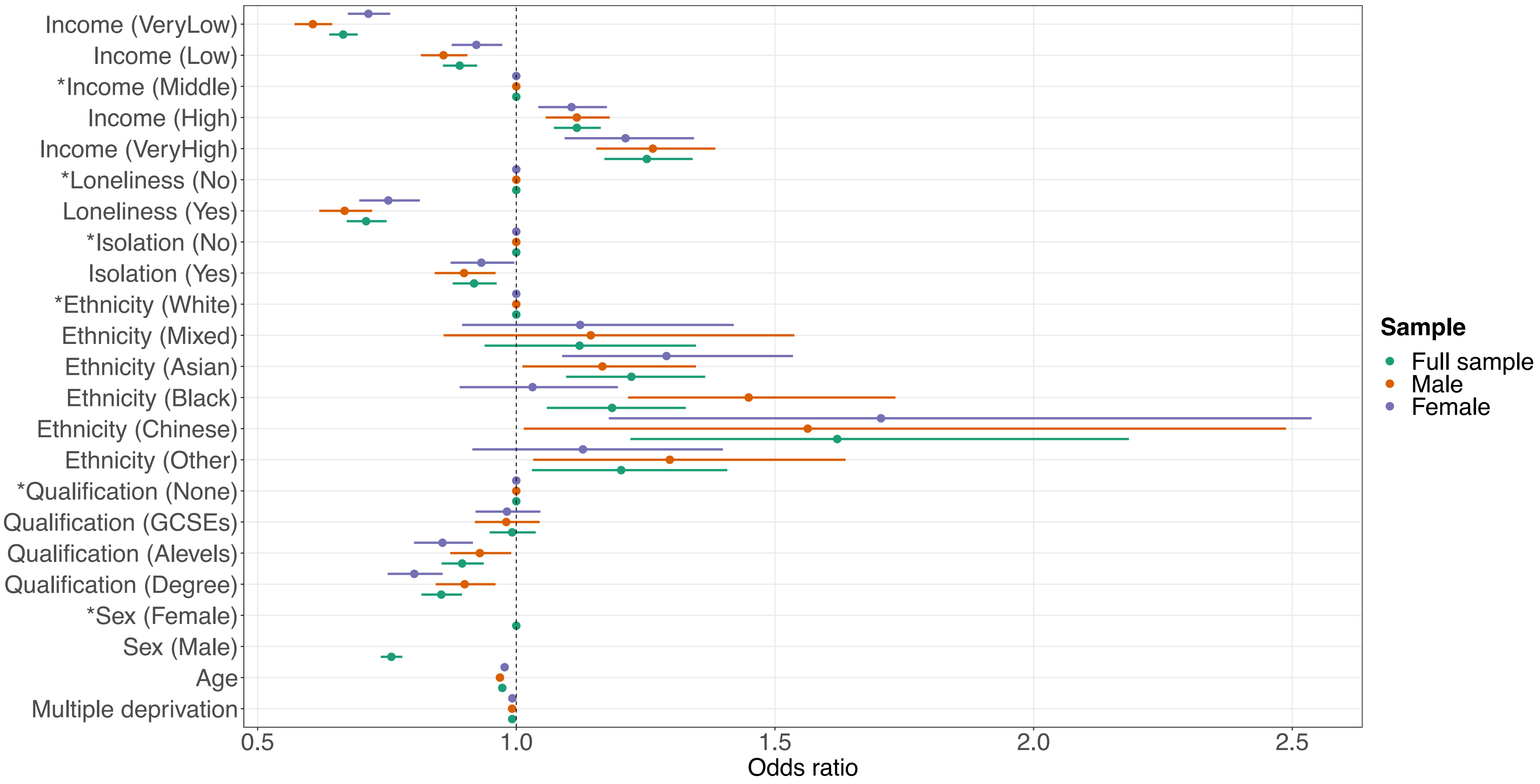

Figure S13. Sociodemographic characteristics and psychosocial factors associated with long-standing illness, stratified by sex. Confidence interval plot (odds ratio ± Bonferroni-adjusted (~99.9%) confidence intervals) for Model 4 (i.e. including all explanatory variables). GCSEs = general certificate of secondary education. *Indicates reference group for categorical explanatory variables. Annual household income groups: very low (<£18,000), low (£18,000–£30,999), middle (£31,000–£51,999), high (£52,000–£100,000) and very high (>£100,000). 'GCSEs' also includes O levels and certificate of secondary education (CSE). 'A levels' also includes national vocational qualification (NVQ), higher national diploma (HND), higher national certificate (HNC) and 'other professional qualifications'.

Lifestyle factors

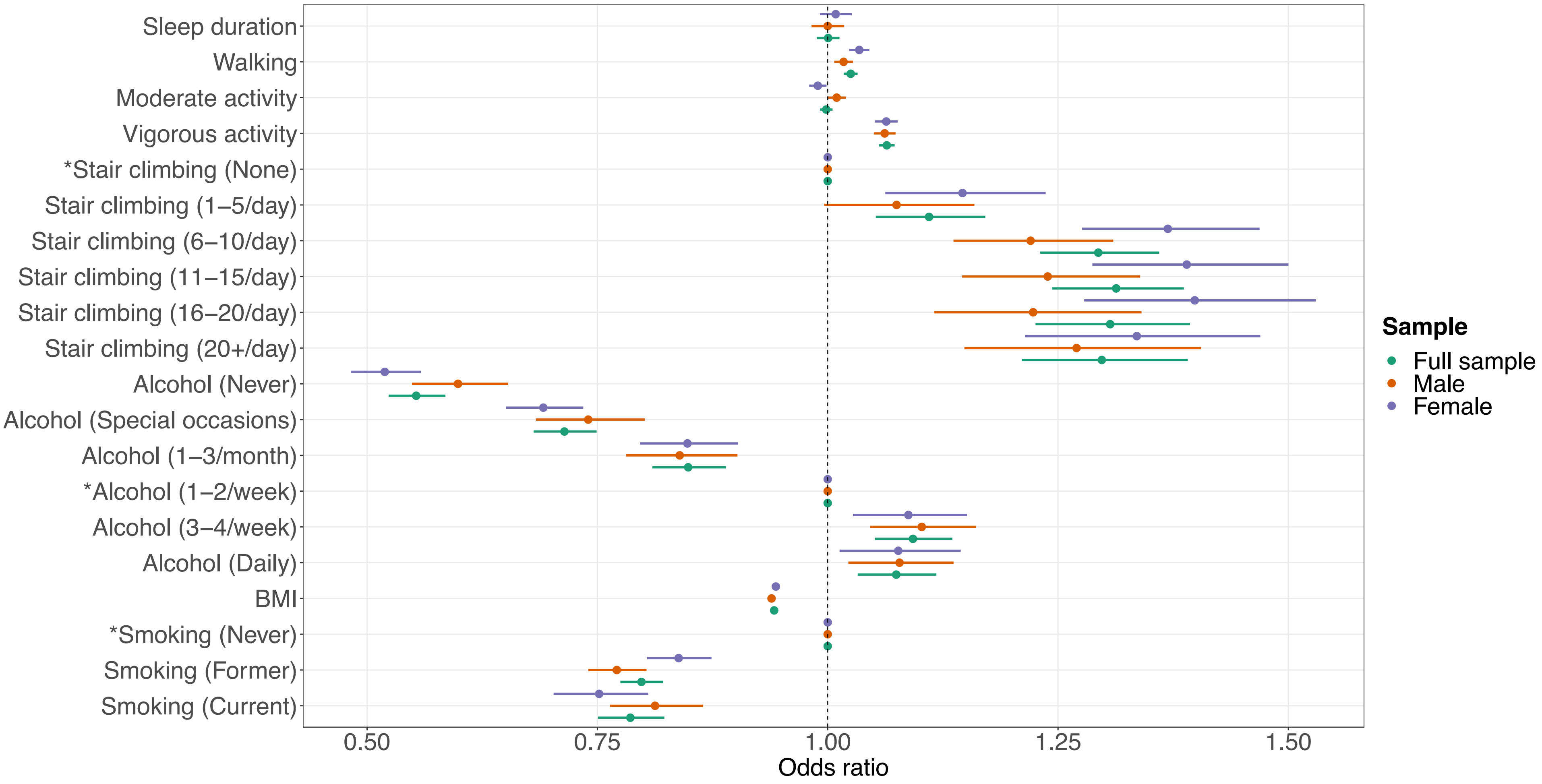

Figure S14. Lifestyle factors associated with long-standing illness, stratified by sex. Confidence interval plot (odds ratio ± Bonferroni-adjusted (~99.9%) confidence intervals) for Model 4 (i.e. including all explanatory variables). BMI = body mass index. *Indicates reference group for categorical explanatory variables.

Environmental exposures

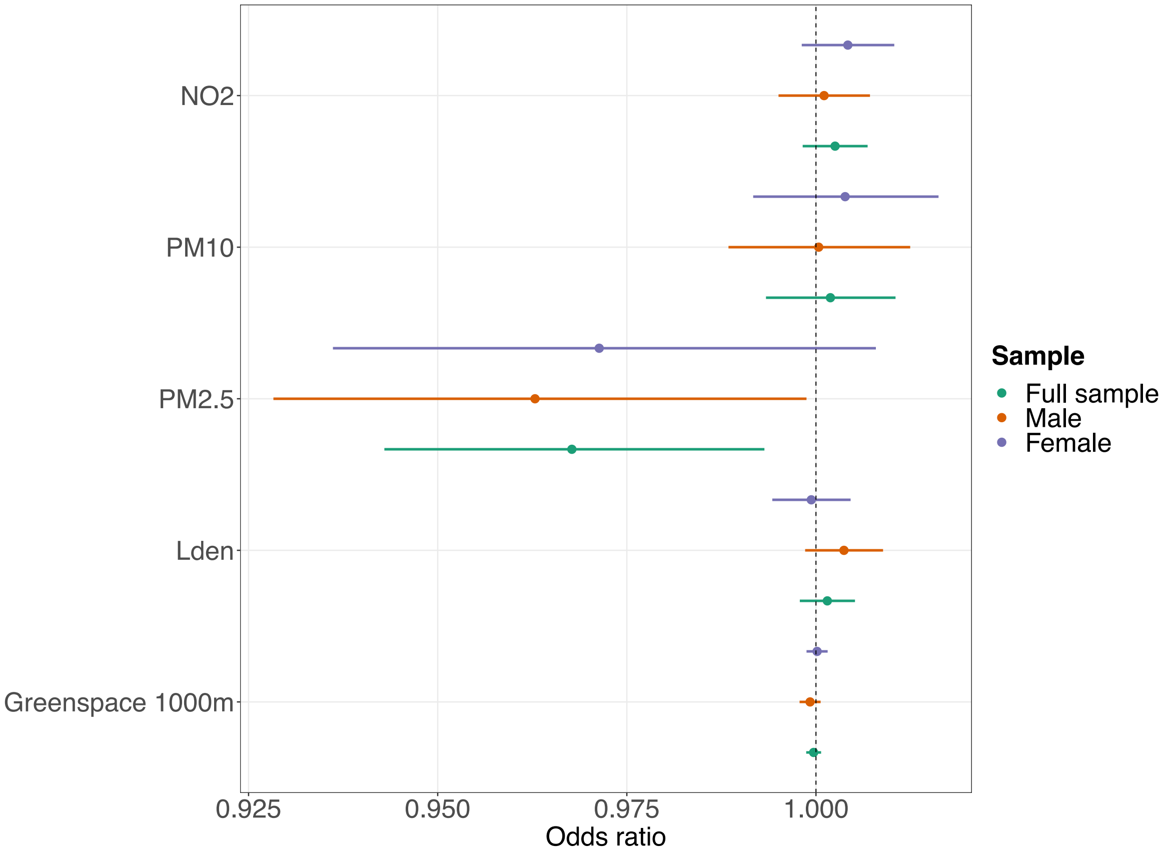

Figure S15. Environmental exposures associated with long-standing illness, stratified by sex. Confidence interval plot (odds ratio ± Bonferroni-adjusted (~99.9%) confidence intervals) for Model 4 (i.e. including all explanatory variables). PM = particulate matter; NO_2 = nitrogen dioxide; L_{den} = day-evening-night noise level.

Tables S39-S42. Regression tables long-standing illness stratified by age

Sociodemographic characteristics

Table S39. **Sociodemographic characteristics associated with long-standing illness stratified by age**

	All participants			Below 65			65 and above			Interaction term	
Term	OR	Bonferroni-corrected CI		OR	Bonferroni-corrected CI		OR	Bonferroni-corrected CI		$p_{Bonf.}$	p_{BH}
**Household income**[1]											
Very low	0.6654	0.6384	0.6934	0.6115	0.5837	0.6405	0.8485	0.7712	0.9334	<0.0001	<0.0001
Low	0.8906	0.8581	0.9244	0.8888	0.8527	0.9263	0.9497	0.8691	1.0375	0.1447	0.0072
Middle	Ref	–	–	Ref	–	–	Ref	–	–	–	–
High	1.1171	1.0726	1.1634	1.1103	1.0637	1.1590	1.0561	0.9228	1.2096	>0.9999	0.0776
Very high	1.2524	1.1703	1.3410	1.2450	1.1599	1.3372	1.1402	0.8881	1.4724	>0.9999	0.0720
**Sex**											
Female	Ref	–	–	Ref	–	–	Ref	–	–	–	–
Male	0.7585	0.7380	0.7797	0.7670	0.7439	0.7909	0.7216	0.6770	0.7690	<0.0001	<0.0001
**Age**	0.9730	0.9711	0.9748	0.9708	0.9685	0.9730	0.9752	0.9558	0.9950	–	–
**Multiple deprivation**	0.9921	0.9910	0.9932	0.9922	0.9910	0.9935	0.9919	0.9893	0.9945	>0.9999	0.6879
**Ethnicity**											
White	Ref	–	–	Ref	–	–	Ref	–	–	–	–
Mixed-race	1.1223	0.9387	1.3474	1.0957	0.9097	1.3257	1.4796	0.7911	2.9027	>0.9999	0.9750
Asian	1.2225	1.0962	1.3652	1.2957	1.1502	1.4621	0.9176	0.6924	1.2196	<0.0001	<0.0001
Black	1.1852	1.0591	1.3277	1.2185	1.0824	1.3735	0.9258	0.6330	1.3562	0.0015	0.0001
Chinese	1.6205	1.2207	2.1842	1.6305	1.2120	2.2309	1.7656	0.6992	5.1866	>0.9999	0.3689
Other	1.2027	1.0301	1.4078	1.2814	1.0861	1.5165	0.7729	0.4805	1.2433	<0.0001	<0.0001
**Highest qualification**											
None	Ref	–	–	Ref	–	–	Ref	–	–	–	–
O levels/GCSEs/CSEs	0.9920	0.9485	1.0375	1.0113	0.9588	1.0665	1.0088	0.9242	1.1011	<0.0001	<0.0001
A levels/NVQ/HND/HNC[2]	0.8953	0.8554	0.9371	0.9185	0.8699	0.9697	0.8943	0.8193	0.9760	<0.0001	<0.0001
Degree	0.8550	0.8167	0.8950	0.8775	0.8312	0.9261	0.8600	0.7843	0.9430	<0.0001	<0.0001

Note: Estimates from Model 4 (i.e. including all explanatory variables). Bonferroni-adjusted (~99.9%) confidence intervals. OR = odds ratio; CI = confidence interval; GCSEs = general certificate of secondary education; CSE = certificate of secondary education; NVQ = national vocational qualification; HND = higher national diploma; HNC = higher national certificate. [1]Annual household income groups: very low (<£18,000), low (£18,000–£30,999), middle (£31,000–£51,999), high (£52,000–£100,000) and very high (>£100,000). [2]also includes 'other professional qualifications'.

Psychosocial factors

Table S40. **Psychosocial factors associated with long-standing illness stratified by age**

	All participants			Below 65			65 and above			Interaction term	
Term	OR	Bonferroni-corrected CI		OR	Bonferroni-corrected CI		OR	Bonferroni-corrected CI		$p_{Bonf.}$	p_{BH}
**Loneliness**											
Not lonely	Ref	–	–	Ref	–	–	Ref	–	–	–	–
Lonely	0.7097	0.6722	0.7494	0.7077	0.6670	0.7511	0.7328	0.6401	0.8392	>0.9999	0.0776
**Social isolation**											
Not isolated	Ref	–	–	Ref	–	–	Ref	–	–	–	–
Isolated	0.9185	0.8770	0.9621	0.9168	0.8711	0.9650	0.9448	0.8468	1.0546	0.0001	<0.0001

Note: Estimates from Model 4 (i.e. including all explanatory variables). Bonferroni-adjusted (~99.9%) confidence intervals. OR = odds ratio; CI = confidence interval.

Lifestyle factors

Table S41. **Lifestyle factors associated with long-standing illness stratified by age**											
	All participants			Below 65			65 and above			Interaction term	
Term	OR	Bonferroni-corrected CI		OR	Bonferroni-corrected CI		OR	Bonferroni-corrected CI		$p_{Bonf.}$	p_{BH}
**Sleep duration** (hours/day)	1.0004	0.9882	1.0128	1.0010	0.9872	1.0150	1.0031	0.9766	1.0302	0.0758	0.0042
**Physical activity** (days/week)[1]											
Walking	1.0250	1.0175	1.0325	1.0215	1.0134	1.0297	1.0441	1.0257	1.0628	<0.0001	<0.0001
Moderate activity	0.9984	0.9916	1.0053	0.9958	0.9881	1.0036	1.0081	0.9931	1.0232	<0.0001	<0.0001
Vigorous activity	1.0641	1.0557	1.0727	1.0665	1.0569	1.0762	1.0551	1.0372	1.0733	>0.9999	0.9750
**Stair climbing frequency**											
None	Ref	–	–	Ref	–	–	Ref	–	–	–	–
1-5/day	1.1101	1.0522	1.1710	1.1256	1.0576	1.1979	1.0512	0.9437	1.1708	<0.0001	<0.0001
6-10/day	1.2937	1.2307	1.3598	1.2962	1.2222	1.3745	1.2988	1.1801	1.4292	0.0002	<0.0001
11-15/day	1.3131	1.2434	1.3866	1.3104	1.2296	1.3964	1.3528	1.2139	1.5075	0.0441	0.0026
16-20/day	1.3066	1.2255	1.3932	1.2799	1.1890	1.3778	1.4677	1.2837	1.6790	>0.9999	0.0631
20+/day	1.2975	1.2108	1.3907	1.2935	1.1957	1.3996	1.3680	1.1744	1.5952	>0.9999	0.1379
**Alcohol intake frequency**											
Never	0.5533	0.5234	0.5850	0.5390	0.5062	0.5739	0.6084	0.5390	0.6867	0.2955	0.0141
Special occasions	0.7142	0.6809	0.7492	0.7070	0.6702	0.7458	0.7452	0.6691	0.8299	0.0211	0.0013
1-3/month	0.8486	0.8096	0.8895	0.8408	0.7984	0.8855	0.8828	0.7877	0.9896	0.1011	0.0053
1-2/week	Ref	–	–	Ref	–	–	Ref	–	–	–	–
3-4/week	1.0925	1.0514	1.1353	1.0794	1.0345	1.1262	1.1667	1.0659	1.2770	>0.9999	0.9558
Daily/almost daily	1.0744	1.0326	1.1180	1.0720	1.0252	1.1211	1.1248	1.0303	1.2279	>0.9999	0.4071
**BMI** (kg/m^2)	0.9419	0.9392	0.9446	0.9425	0.9396	0.9455	0.9392	0.9324	0.9461	<0.0001	<0.0001
**Smoking status**											
Never	Ref	–	–	Ref	–	–	Ref	–	–	–	–
Former	0.7978	0.7749	0.8213	0.8114	0.7853	0.8385	0.7592	0.7120	0.8094	<0.0001	<0.0001
Current	0.7858	0.7506	0.8227	0.7824	0.7447	0.8222	0.8565	0.7578	0.9687	>0.9999	0.2871

Note: Estimates from Model 4 (i.e. including all explanatory variables). Bonferroni-adjusted (~99.9%) confidence intervals. OR = odds ratio; CI = confidence interval; BMI = body mass index. [1]number of days per week engaging in these activities for 10+ minutes continuously.

Table S42. **Environmental exposures associated with long-standing illness stratified by age**

	All participants			Below 65			65 and above			Interaction term	
Term	OR	Bonferroni-corrected CI		OR	Bonferroni-corrected CI		OR	Bonferroni-corrected CI		$p_{Bonf.}$	p_{BH}
**$PM_{2.5}$**	0.9677	0.9430	0.9932	0.9704	0.9428	0.9988	0.9517	0.8966	1.0102	>0.9999	0.2850
**PM_{10}**	1.0019	0.9934	1.0105	1.0016	0.9921	1.0112	1.0028	0.9834	1.0226	>0.9999	0.9425
**NO_2**	1.0025	0.9982	1.0068	1.0025	0.9977	1.0073	1.0036	0.9938	1.0134	>0.9999	0.3335
**L_{den}**	1.0015	0.9979	1.0052	1.0020	0.9980	1.0061	0.9995	0.9911	1.0079	>0.9999	0.0776
**Greenspace** 1000m	0.9997	0.9987	1.0007	0.9998	0.9987	1.0009	0.9995	0.9972	1.0018	>0.9999	0.6879

Note: Estimates from Model 4 (i.e. including all explanatory variables). Bonferroni-adjusted (~99.9%) confidence intervals. OR = odds ratio; CI = confidence interval; PM = particulate matter; NO_2 = nitrogen dioxide; L_{den} = day-evening-night noise level.

Figures S16-S18. Confidence interval plots long-standing illness stratified by age

Sociodemographic and psychosocial factors

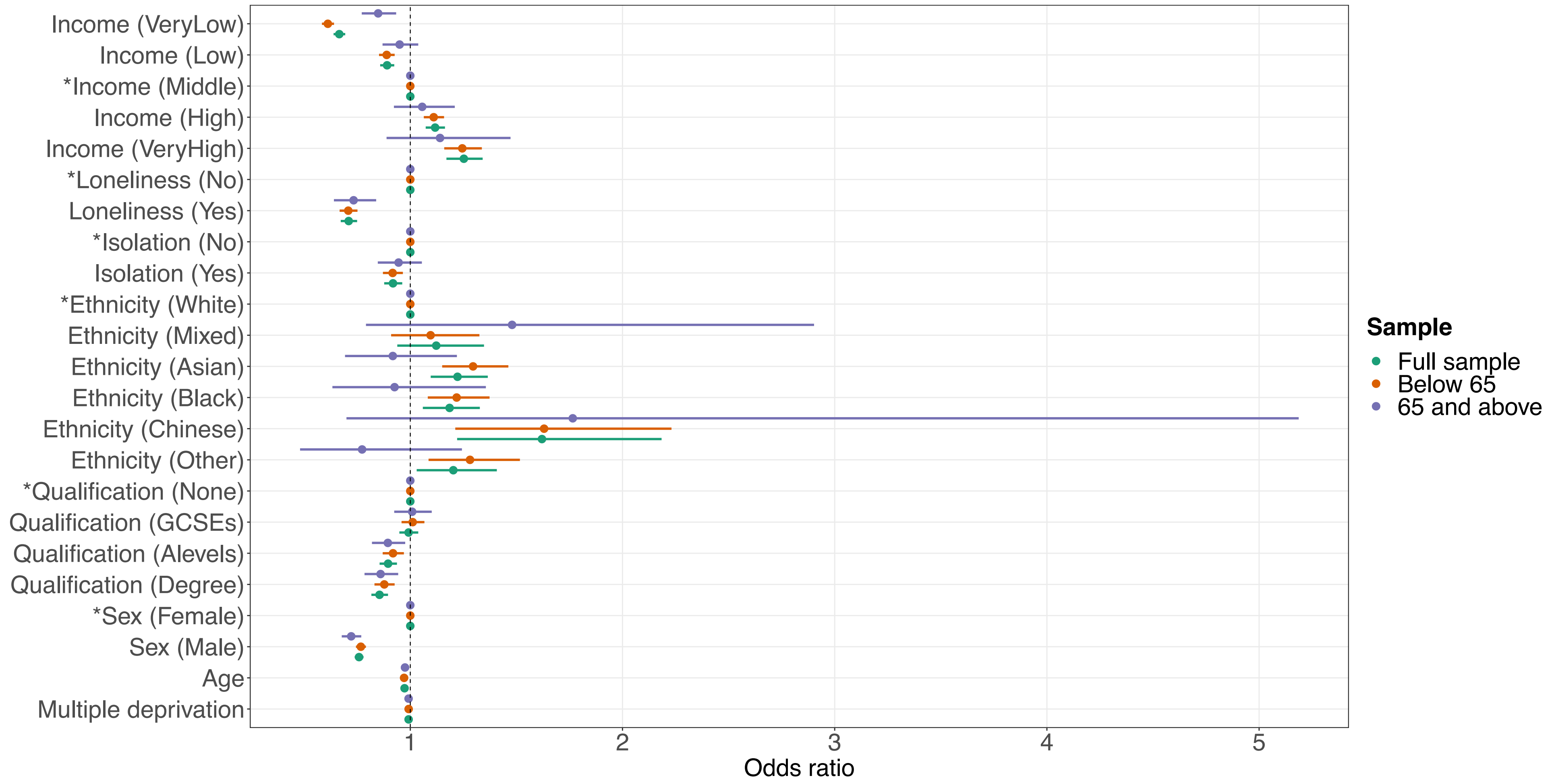

Figure S16. Sociodemographic characteristics and psychosocial factors associated with long-standing illness, stratified by age. Confidence interval plot (odds ratio ± Bonferroni-adjusted (~99.9%) confidence intervals) for Model 4 (i.e. including all explanatory variables). GCSEs = general certificate of secondary education. *Indicates reference group for categorical explanatory variables. Annual household income groups: very low (<£18,000), low (£18,000–£30,999), middle (£31,000–£51,999), high (£52,000–£100,000) and very high (>£100,000). 'GCSEs' also includes O levels and certificate of secondary education (CSE). 'A levels' also includes national vocational qualification (NVQ), higher national diploma (HND), higher national certificate (HNC) and 'other professional qualifications'.

Lifestyle factors

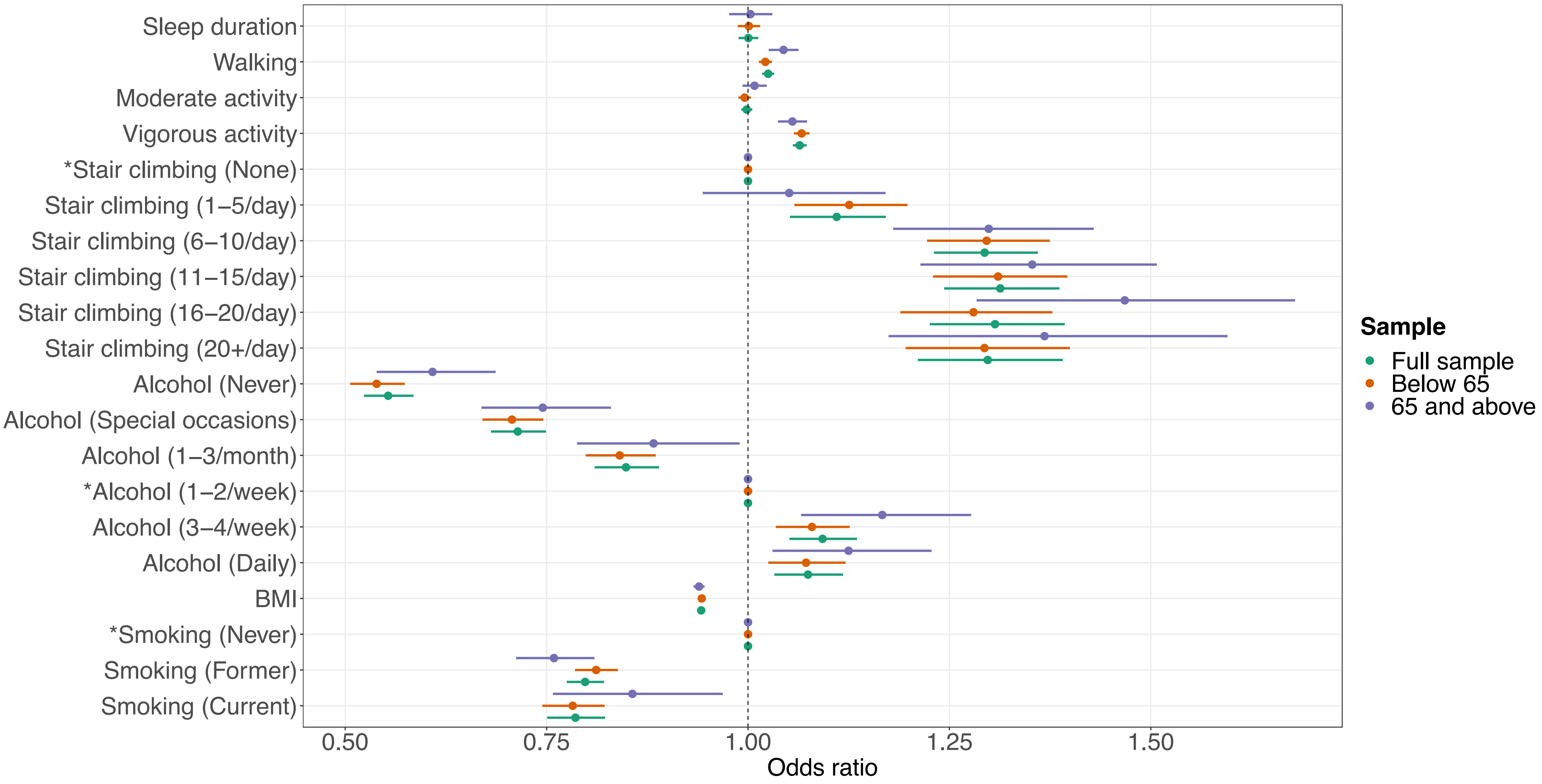

Figure S17. Lifestyle factors associated with long-standing illness, stratified by age. Confidence interval plot (odds ratio ± Bonferroni-adjusted (~99.9%) confidence intervals) for Model 4 (i.e. including all explanatory variables). BMI = body mass index. *Indicates reference group for categorical explanatory variables.

Environmental exposures

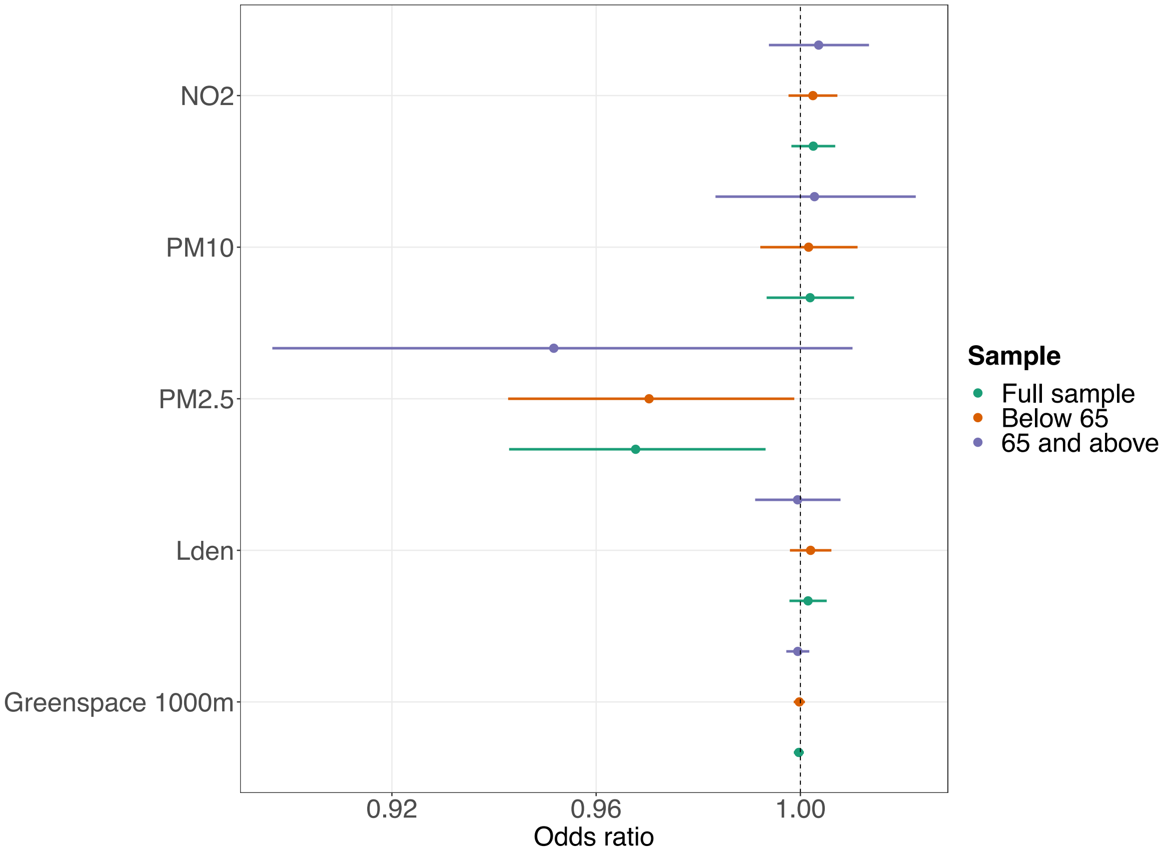

Figure S18. Environmental exposures associated with long-standing illness, stratified by age. Confidence interval plot (odds ratio ± Bonferroni-adjusted (~99.9%) confidence intervals) for Model 4 (i.e. including all explanatory variables). PM = particulate matter; NO_2 = nitrogen dioxide; L_{den} = day-evening-night noise level.

Tables S43-S46. Regression tables self-rated health stratified by sex

Sociodemographic characteristics

Table S43. **Sociodemographic characteristics associated with self-rated health stratified by sex**

	All participants			Male			Female			Interaction term	
Term	OR	Bonferroni-corrected CI		OR	Bonferroni-corrected CI		OR	Bonferroni-corrected CI		$p_{Bonf.}$	p_{BH}
**Household income**[1]											
Very low	0.6192	0.5960	0.6432	0.5733	0.5418	0.6065	0.6505	0.6175	0.6852	<0.0001	<0.0001
Low	0.8512	0.8234	0.8799	0.8300	0.7912	0.8706	0.8650	0.8259	0.9059	0.1481	0.0067
Middle	Ref	–	–	Ref	–	–	Ref	–	–	–	–
High	1.2102	1.1691	1.2526	1.2497	1.1910	1.3113	1.1722	1.1156	1.2318	0.0002	<0.0001
Very high	1.6364	1.5491	1.7286	1.7228	1.5975	1.8580	1.5552	1.4361	1.6842	<0.0001	<0.0001
**Sex**											
Female	Ref	–	–	Ref	–	–	Ref	–	–	–	–
Male	0.7836	0.7648	0.8030	–	–	–	–	–	–	–	–
**Age**	1.0113	1.0097	1.0130	1.0118	1.0094	1.0142	1.0108	1.0084	1.0132	<0.0001	<0.0001
**Multiple deprivation**	0.9923	0.9913	0.9933	0.9915	0.9901	0.9930	0.9932	0.9917	0.9946	<0.0001	<0.0001
**Ethnicity**											
White	Ref	–	–	Ref	–	–	Ref	–	–	–	–
Mixed-race	0.9019	0.7729	1.0527	1.0413	0.8098	1.3399	0.8229	0.6762	1.0020	0.5618	0.0208
Asian	0.5995	0.5445	0.6602	0.6139	0.5407	0.6970	0.5739	0.4948	0.6658	0.8350	0.0288
Black	1.0896	0.9839	1.2069	1.4325	1.2258	1.6745	0.8748	0.7643	1.0017	<0.0001	<0.0001
Chinese	0.6783	0.5413	0.8506	0.7109	0.4948	1.0236	0.6773	0.5069	0.9063	>0.9999	0.2264
Other	0.8903	0.7746	1.0235	1.1508	0.9361	1.4154	0.7210	0.5972	0.8710	<0.0001	<0.0001
**Highest qualification**											
None	Ref	–	–	Ref	–	–	Ref	–	–	–	–
O levels/GCSEs/CSEs	1.2420	1.1920	1.2941	1.2168	1.1471	1.2908	1.2412	1.1716	1.3149	0.7710	0.0275
A levels/NVQ/HND/HNC[2]	1.2876	1.2345	1.3429	1.2761	1.2030	1.3536	1.2892	1.2138	1.3692	0.0201	0.0010
Degree	1.5031	1.4412	1.5676	1.5429	1.4537	1.6376	1.4286	1.3456	1.5166	<0.0001	<0.0001

Note: Estimates from Model 4 (i.e. including all explanatory variables). Bonferroni-adjusted (~99.9%) confidence intervals. OR = odds ratio; CI = confidence interval; GCSEs = general certificate of secondary education; CSE = certificate of secondary education; NVQ = national vocational qualification; HND = higher national diploma; HNC = higher national certificate. For categorical explanatory variables the odds ratios indicate the changes in odds of reporting better self-rated health associated with the explanatory variable group relative to the reference group. Odds ratios for continuous explanatory variables indicate proportional odds ratios for a 1-unit increase in the explanatory variable on level of self-rated health. [1]Annual household income groups: very low (<£18,000), low (£18,000–£30,999), middle (£31,000–£51,999), high (£52,000–£100,000) and very high (>£100,000). [2]also includes 'other professional qualifications'.

Psychosocial factors

Table S44. **Psychosocial factors associated with self-rated health stratified by sex**										
	All participants			Male			Female			Interaction term	
Term	OR	Bonferroni-corrected CI		OR	Bonferroni-corrected CI		OR	Bonferroni-corrected CI		$p_{Bonf.}$	p_{BH}
**Loneliness**											
Not lonely	Ref	–	–	Ref	–	–	Ref	–	–	–	–
Lonely	0.4921	0.4678	0.5176	0.4901	0.4564	0.5263	0.4971	0.4626	0.5342	0.0172	0.0009
**Social isolation**											
Not isolated	Ref	–	–	Ref	–	–	Ref	–	–	–	–
Isolated	0.8593	0.8233	0.8970	0.8311	0.7821	0.8831	0.8805	0.8286	0.9358	<0.0001	<0.0001

Note: Estimates from Model 4 (i.e. including all explanatory variables). Bonferroni-adjusted (~99.9%) confidence intervals. OR = odds ratio; CI = confidence interval. For categorical explanatory variables the odds ratios indicate the changes in odds of reporting better self-rated health associated with the explanatory variable group relative to the reference group.

Table S45. **Lifestyle factors associated with self-rated health stratified by sex**

	All participants			Male			Female			Interaction term	
Term	OR	Bonferroni-corrected CI		OR	Bonferroni-corrected CI		OR	Bonferroni-corrected CI		$p_{\text{Bonf.}}$	p_{BH}
**Sleep duration** (hours/day)	1.0730	1.0609	1.0852	1.0685	1.0510	1.0864	1.0812	1.0644	1.0983	>0.9999	0.1448
**Physical activity** (days/week)[1]											
Walking	1.0522	1.0454	1.0591	1.0477	1.0382	1.0573	1.0577	1.0477	1.0677	0.1603	0.0070
Moderate activity	1.0132	1.0070	1.0195	1.0256	1.0164	1.0350	1.0050	0.9966	1.0134	<0.0001	<0.0001
Vigorous activity	1.1668	1.1585	1.1751	1.1766	1.1648	1.1886	1.1506	1.1390	1.1624	<0.0001	<0.0001
**Stair climbing frequency**											
None	Ref	–	–	Ref	–	–	Ref	–	–	–	–
1-5/day	1.0533	1.0019	1.1074	1.0459	0.9744	1.1226	1.0578	0.9854	1.1355	>0.9999	0.5955
6-10/day	1.2293	1.1733	1.2878	1.2207	1.1422	1.3045	1.2290	1.1511	1.3121	0.4963	0.0191
11-15/day	1.2892	1.2260	1.3557	1.3136	1.2220	1.4119	1.2642	1.1785	1.3562	0.0001	<0.0001
16-20/day	1.2991	1.2258	1.3767	1.2994	1.1944	1.4135	1.3008	1.2004	1.4097	0.0168	0.0009
20+/day	1.3652	1.2828	1.4529	1.4561	1.3287	1.5956	1.3054	1.1986	1.4217	<0.0001	<0.0001
**Alcohol intake frequency**											
Never	0.6624	0.6285	0.6981	0.7264	0.6688	0.7890	0.6123	0.5717	0.6558	0.0033	0.0002
Special occasions	0.7565	0.7240	0.7904	0.7772	0.7214	0.8373	0.7253	0.6861	0.7666	>0.9999	0.6945
1-3/month	0.9201	0.8821	0.9597	0.9173	0.8591	0.9795	0.9101	0.8610	0.9621	>0.9999	0.4250
1-2/week	Ref	–	–	Ref	–	–	Ref	–	–	–	–
3-4/week	1.0520	1.0178	1.0875	1.0396	0.9926	1.0888	1.0700	1.0203	1.1222	>0.9999	0.5955
Daily/almost daily	1.0264	0.9913	1.0628	0.9998	0.9535	1.0484	1.0609	1.0074	1.1172	>0.9999	0.3959
**BMI** (kg/m^2)	0.9045	0.9020	0.9069	0.8908	0.8870	0.8946	0.9119	0.9087	0.9150	<0.0001	<0.0001
**Smoking status**											
Never	Ref	–	–	Ref	–	–	Ref	–	–	–	–
Former	0.8128	0.7919	0.8342	0.7768	0.7484	0.8063	0.8619	0.8309	0.8941	<0.0001	<0.0001
Current	0.5029	0.4825	0.5242	0.5007	0.4733	0.5297	0.5005	0.4706	0.5322	0.0015	0.0001

Note: Estimates from Model 4 (i.e. including all explanatory variables). Bonferroni-adjusted (~99.9%) confidence intervals. OR = odds ratio; CI = confidence interval; BMI = body mass index. For categorical explanatory variables the odds ratios indicate the changes in odds of reporting better self-rated health associated with the explanatory variable group relative to the reference group. Odds ratios for continuous explanatory variables indicate proportional odds ratios for a 1-unit increase in the explanatory variable on level of self-rated health. [1]number of days per week engaging in these activities for 10+ minutes continuously.

Table S46. **Environmental exposures associated with self-rated health stratified by sex**

	All participants			Male			Female			Interaction term	
Term	OR	Bonferroni-corrected CI		OR	Bonferroni-corrected CI		OR	Bonferroni-corrected CI		$p_{Bonf.}$	p_{BH}
**$PM_{2.5}$**	1.0283	1.0047	1.0525	1.0267	0.9933	1.0612	1.0289	0.9959	1.0630	0.0031	0.0002
**PM_{10}**	0.9979	0.9904	1.0055	0.9959	0.9852	1.0067	1.0003	0.9898	1.0110	0.2851	0.0119
**NO_2**	0.9948	0.9910	0.9986	0.9955	0.9901	1.0009	0.9945	0.9892	0.9998	0.3695	0.0148
**L_{den}**	1.0025	0.9993	1.0058	1.0016	0.9970	1.0063	1.0035	0.9989	1.0080	>0.9999	0.1412
**Greenspace** 1000m	1.0007	0.9998	1.0015	1.0010	0.9998	1.0023	1.0004	0.9992	1.0016	>0.9999	0.0889

Note: Estimates from Model 4 (i.e. including all explanatory variables). Bonferroni-adjusted (~99.9%) confidence intervals. OR = odds ratio; CI = confidence interval; PM = particulate matter; NO_2 = nitrogen dioxide; L_{den} = day-evening-night noise level. Odds ratios indicate proportional odds ratios for a 1-unit increase in the explanatory variable on level of self-rated health.

Figures S19-S21. Confidence interval plots self-rated health stratified by sex

Sociodemographic and psychosocial factors

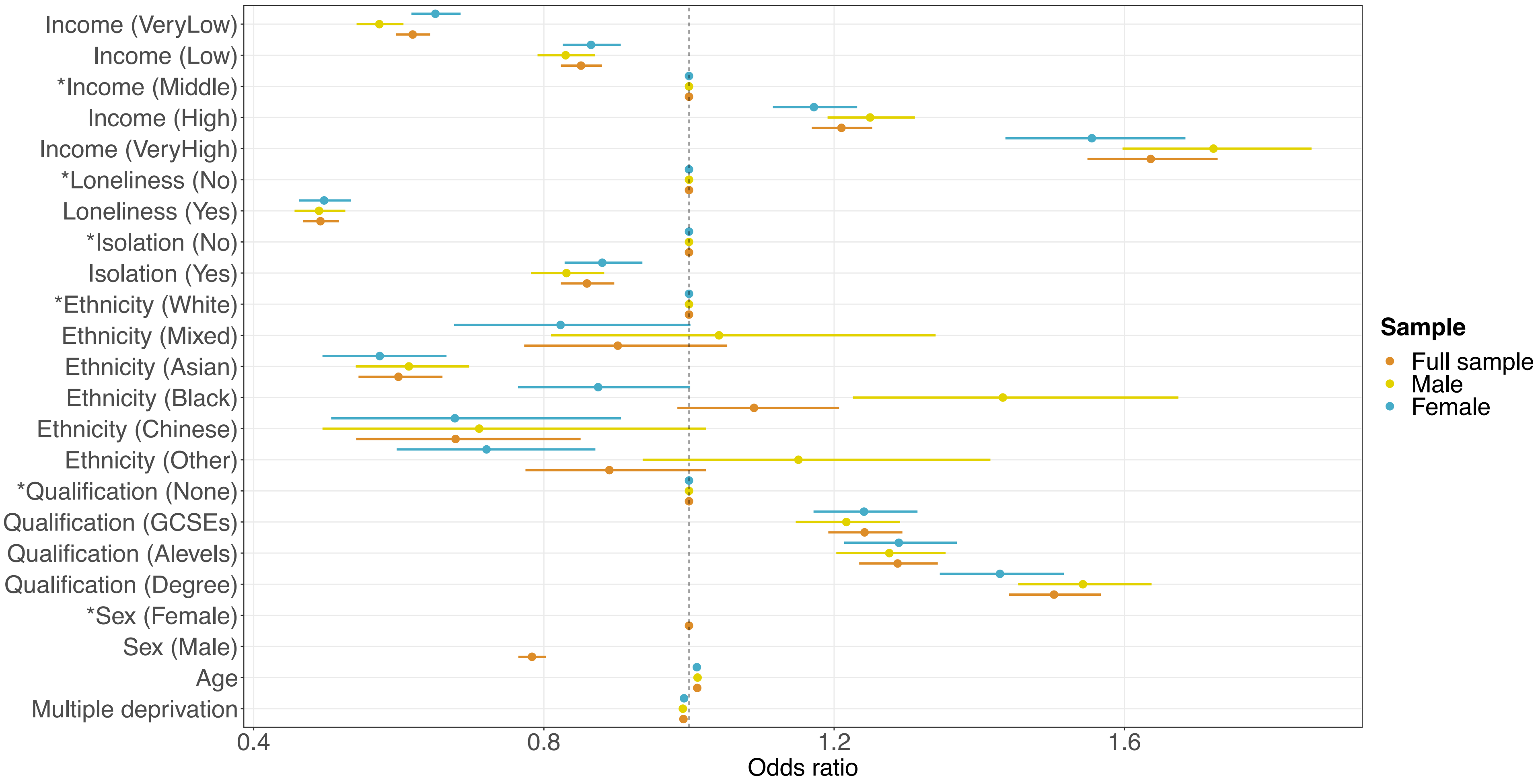

Figure S19. Sociodemographic characteristics and psychosocial factors associated with self-rated health, stratified by sex. Confidence interval plot (odds ratio ± Bonferroni-adjusted (~99.9%) confidence intervals) for Model 4 (i.e. including all explanatory variables). GCSEs = general certificate of secondary education. *Indicates reference group for categorical explanatory variables. Annual household income groups: very low (<£18,000), low (£18,000–£30,999), middle (£31,000–£51,999), high (£52,000–£100,000) and very high (>£100,000). 'GCSEs' also includes O levels and certificate of secondary education (CSE). 'A levels' also includes national vocational qualification (NVQ), higher national diploma (HND), higher national certificate (HNC) and 'other professional qualifications'.

Lifestyle factors

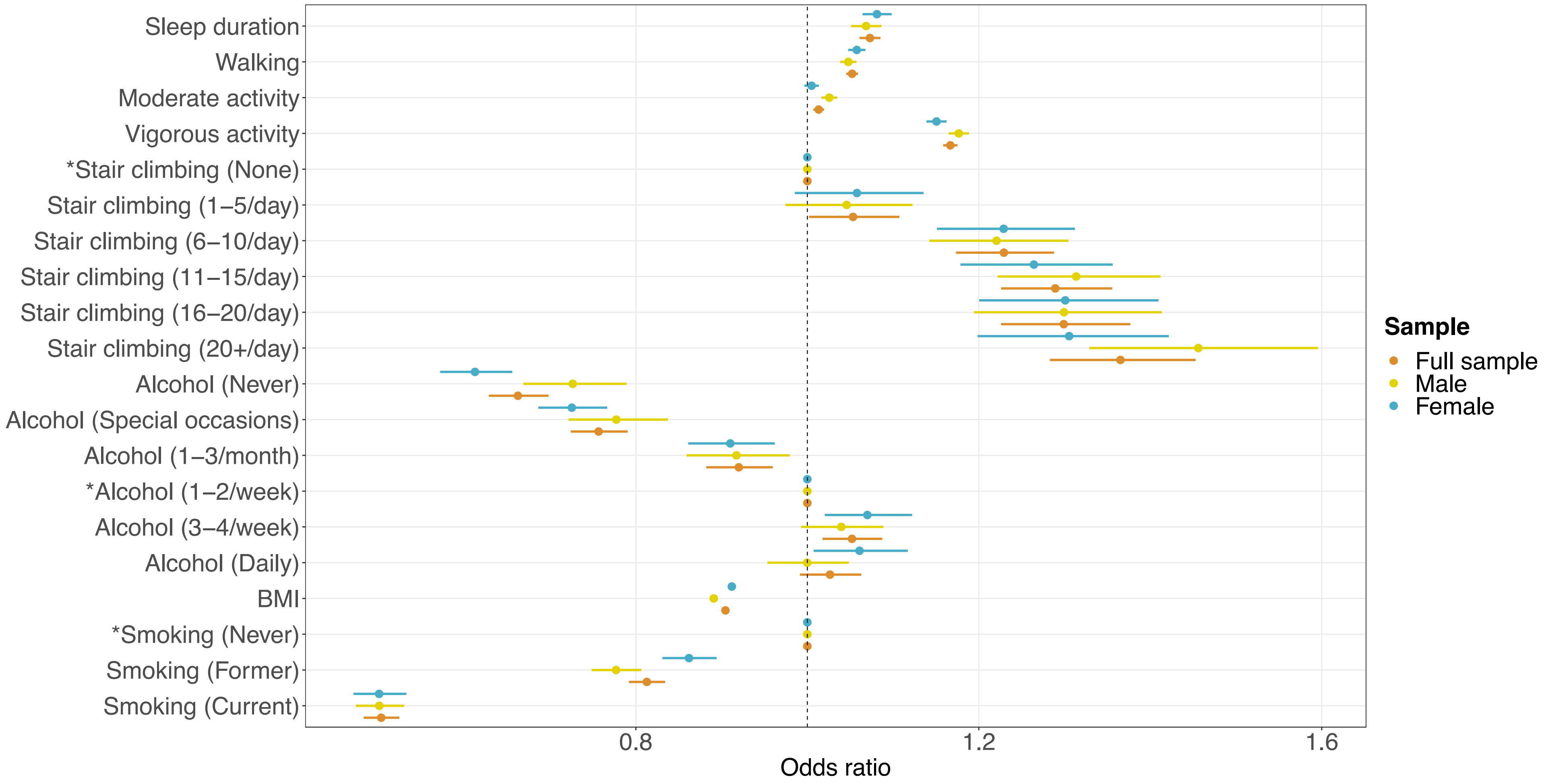

Figure S20. Lifestyle factors associated with self-rated health, stratified by sex. Confidence interval plot (odds ratio ± Bonferroni-adjusted (~99.9%) confidence intervals) for Model 4 (i.e. including all explanatory variables). BMI = body mass index. *Indicates reference group for categorical explanatory variables.

Environmental exposures

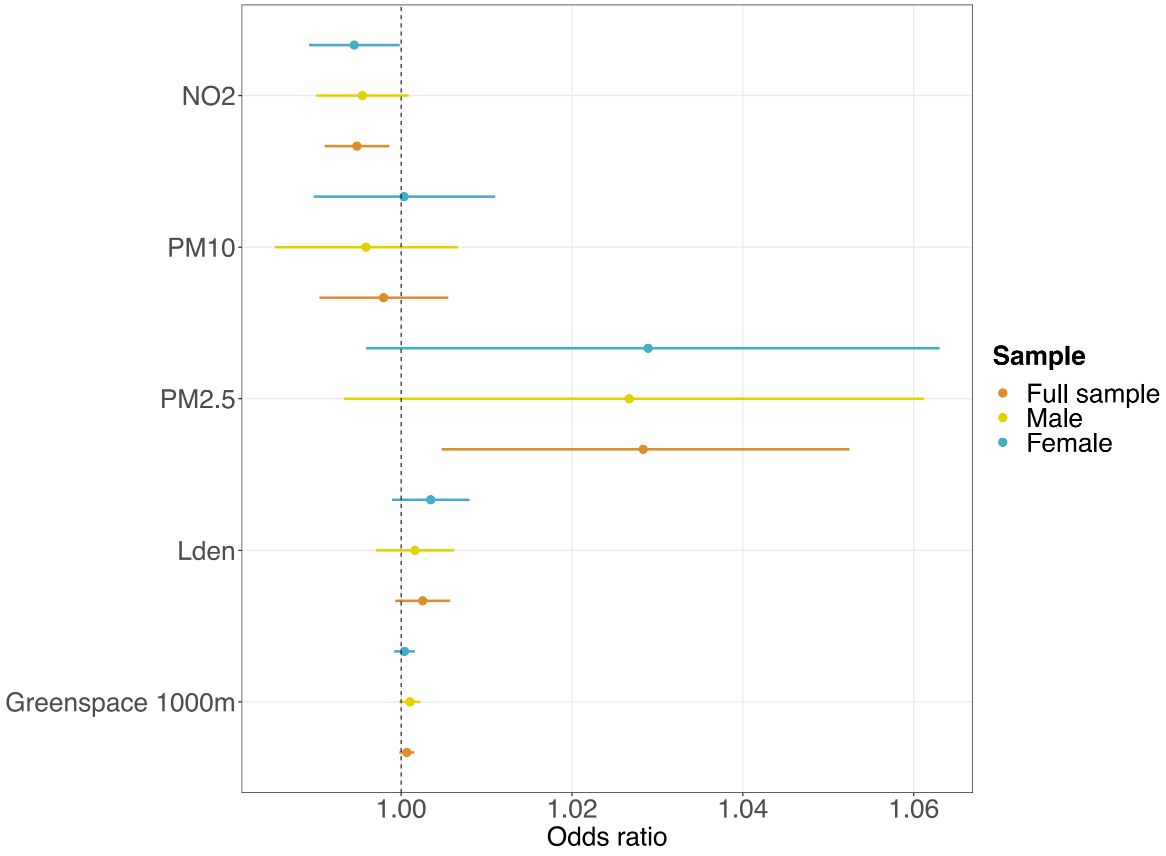

Figure S21. Environmental exposures associated with self-rated health, stratified by sex. Confidence interval plot (odds ratio ± Bonferroni-adjusted (~99.9%) confidence intervals) for Model 4 (i.e. including all explanatory variables). PM = particulate matter; NO_2 = nitrogen dioxide; L_{den} = day-evening-night noise level.

Tables S47-S50. Regression tables self-rated health stratified by age

Sociodemographic characteristics

Table S47. **Sociodemographic characteristics associated with self-rated health stratified by age**

	All participants			Below 65			65 and above			Interaction term	
Term	OR	Bonferroni-corrected CI		OR	Bonferroni-corrected CI		OR	Bonferroni-corrected CI		$p_{Bonf.}$	p_{BH}
**Household income**[1]											
Very low	0.6192	0.5960	0.6432	0.5873	0.5627	0.6130	0.7057	0.6442	0.7729	<0.0001	<0.0001
Low	0.8512	0.8234	0.8799	0.8561	0.8255	0.8879	0.8614	0.7922	0.9367	0.6464	0.0249
Middle	Ref	–	–	Ref	–	–	Ref	–	–	–	–
High	1.2102	1.1691	1.2526	1.2062	1.1635	1.2504	1.1177	0.9844	1.2691	0.0288	0.0014
Very high	1.6364	1.5491	1.7286	1.6386	1.5483	1.7341	1.2259	0.9692	1.5498	0.0004	<0.0001
**Sex**											
Female	Ref	–	–	Ref	–	–	Ref	–	–	–	–
Male	0.7836	0.7648	0.8030	0.7808	0.7601	0.8019	0.8118	0.7643	0.8622	<0.0001	<0.0001
**Age**	1.0113	1.0097	1.0130	1.0120	1.0010	1.0140	0.9978	0.9789	1.0171	–	–
**Multiple deprivation**	0.9923	0.9913	0.9933	0.9922	0.9911	0.9933	0.9938	0.9913	0.9963	>0.9999	0.8416
**Ethnicity**											
White	Ref	–	–	Ref	–	–	Ref	–	–	–	–
Mixed-race	0.9019	0.7729	1.0527	0.9212	0.7851	1.0811	0.7125	0.3969	1.2876	>0.9999	0.3609
Asian	0.5995	0.5445	0.6602	0.6297	0.5680	0.6983	0.4443	0.3399	0.5817	<0.0001	<0.0001
Black	1.0896	0.9839	1.2069	1.1503	1.0343	1.2796	0.6069	0.4243	0.8709	<0.0001	<0.0001
Chinese	0.6783	0.5413	0.8506	0.6780	0.5366	0.8573	0.6584	0.2825	1.5576	>0.9999	0.5891
Other	0.8903	0.7746	1.0235	0.9262	0.8005	1.0719	0.6205	0.3924	0.9868	<0.0001	<0.0001
**Highest qualification**											
None	Ref	–	–	Ref	–	–	Ref	–	–	–	–
O levels/GCSEs/CSEs	1.2420	1.1920	1.2941	1.2790	1.2187	1.3423	1.2593	1.1587	1.3688	<0.0001	<0.0001
A levels/NVQ/HND/HNC[2]	1.2876	1.2345	1.3429	1.3358	1.2713	1.4036	1.2424	1.1423	1.3514	<0.0001	<0.0001
Degree	1.5031	1.4412	1.5676	1.5655	1.4905	1.6443	1.4066	1.2878	1.5364	<0.0001	<0.0001

Note: Estimates from Model 4 (i.e. including all explanatory variables). Bonferroni-adjusted (~99.9%) confidence intervals. OR = odds ratio; CI = confidence interval; GCSEs = general certificate of secondary education; CSE = certificate of secondary education; NVQ = national vocational qualification; HND = higher national diploma; HNC = higher national certificate. For categorical explanatory variables the odds ratios indicate the changes in odds of reporting better self-rated health associated with the explanatory variable group relative to the reference group. Odds ratios for continuous explanatory variables indicate proportional odds ratios for a 1-unit increase in the explanatory variable on level of self-rated health. [1]Annual household income groups: very low (<£18,000), low (£18,000–£30,999), middle (£31,000–£51,999), high (£52,000–£100,000) and very high (>£100,000). [2]also includes 'other professional qualifications'.

Table S48. **Psychosocial factors associated with self-rated health stratified by age**

	All participants			Below 65			65 and above			Interaction term	
Term	OR	Bonferroni-corrected CI		OR	Bonferroni-corrected CI		OR	Bonferroni-corrected CI		$p_{\text{Bonf.}}$	p_{BH}
**Loneliness**											
Not lonely	Ref	–	–	Ref	–	–	Ref	–	–	–	–
Lonely	0.4921	0.4678	0.5176	0.4853	0.4594	0.5127	0.5460	0.4798	0.6216	0.0016	0.0001
**Social isolation**											
Not isolated	Ref	–	–	Ref	–	–	Ref	–	–	–	–
Isolated	0.8593	0.8233	0.8970	0.8528	0.8137	0.8938	0.9057	0.8149	1.0067	0.0334	0.0015

Note: Estimates from Model 4 (i.e. including all explanatory variables). Bonferroni-adjusted (~99.9%) confidence intervals. OR = odds ratio; CI = confidence interval. For categorical explanatory variables the odds ratios indicate the changes in odds of reporting better self-rated health associated with the explanatory variable group relative to the reference group.

Table S49. **Lifestyle factors associated with self-rated health stratified by age**

	All participants			Below 65			65 and above			Interaction term	
Term	OR	Bonferroni-corrected CI		OR	Bonferroni-corrected CI		OR	Bonferroni-corrected CI		$p_{Bonf.}$	p_{BH}
**Sleep duration** (hours/day)	1.0730	1.0609	1.0852	1.0790	1.0654	1.0927	1.0453	1.0185	1.0727	0.0193	0.0010
**Physical activity** (days/week)[1]											
Walking	1.0522	1.0454	1.0591	1.0474	1.0400	1.0548	1.0836	1.0652	1.1024	0.0005	<0.0001
Moderate activity	1.0132	1.0070	1.0195	1.0112	1.0044	1.0181	1.0218	1.0073	1.0365	>0.9999	0.3609
Vigorous activity	1.1668	1.1585	1.1751	1.1745	1.1652	1.1839	1.1352	1.1168	1.1540	<0.0001	<0.0001
**Stair climbing frequency**											
None	Ref	–	–	Ref	–	–	Ref	–	–	–	–
1-5/day	1.0533	1.0019	1.1074	1.0797	1.0192	1.1437	0.9617	0.8660	1.0679	0.0006	<0.0001
6-10/day	1.2293	1.1733	1.2878	1.2422	1.1767	1.3114	1.2165	1.1086	1.3348	0.4794	0.0192
11-15/day	1.2892	1.2260	1.3557	1.2980	1.2249	1.3755	1.3092	1.1794	1.4532	>0.9999	0.0663
16-20/day	1.2991	1.2258	1.3767	1.3031	1.2198	1.3921	1.3533	1.1920	1.5364	>0.9999	0.3178
20+/day	1.3652	1.2828	1.4529	1.3693	1.2767	1.4687	1.4508	1.2551	1.6770	>0.9999	0.0405
**Alcohol intake frequency**											
Never	0.6624	0.6285	0.6981	0.6645	0.6265	0.7048	0.6660	0.5922	0.7490	0.0324	0.0015
Special occasions	0.7565	0.7240	0.7904	0.7546	0.7189	0.7921	0.7807	0.7037	0.8662	>0.9999	0.0689
1-3/month	0.9201	0.8821	0.9597	0.9123	0.8716	0.9549	0.9756	0.8743	1.0887	0.8692	0.0322
1-2/week	Ref	–	–	Ref	–	–	Ref	–	–	–	–
3-4/week	1.0520	1.0178	1.0875	1.0384	1.0017	1.0765	1.1298	1.0374	1.2304	>0.9999	0.3609
Daily/almost daily	1.0264	0.9913	1.0628	1.0044	0.9665	1.0437	1.1712	1.0773	1.2732	<0.0001	<0.0001
**BMI** (kg/m^2)	0.9045	0.9020	0.9069	0.9025	0.8999	0.9052	0.9180	0.9116	0.9245	<0.0001	<0.0001
**Smoking status**											
Never	Ref	–	–	Ref	–	–	Ref	–	–	–	–
Former	0.8128	0.7919	0.8342	0.8170	0.7938	0.8409	0.7808	0.7343	0.8302	>0.9999	0.6825
Current	0.5029	0.4825	0.5242	0.4964	0.4748	0.5188	0.5844	0.5196	0.6574	<0.0001	<0.0001

Note: Estimates from Model 4 (i.e. including all explanatory variables). Bonferroni-adjusted (~99.9%) confidence intervals. OR = odds ratio; CI = confidence interval; BMI = body mass index. For categorical explanatory variables the odds ratios indicate the changes in odds of reporting better self-rated health associated with the explanatory variable group relative to the reference group. Odds ratios for continuous explanatory variables indicate proportional odds ratios for a 1-unit increase in the explanatory variable on level of self-rated health. [1]number of days per week engaging in these activities for 10+ minutes continuously.

Table S50. **Environmental exposures associated with self-rated health stratified by age**

	All participants			Below 65			65 and above			Interaction term	
Term	OR	Bonferroni-corrected CI		OR	Bonferroni-corrected CI		OR	Bonferroni-corrected CI		$p_{Bonf.}$	p_{BH}
**$PM_{2.5}$**	1.0283	1.0047	1.0525	1.0290	1.0031	1.0555	1.0195	0.9631	1.0793	0.0001	<0.0001
**PM_{10}**	0.9979	0.9904	1.0055	0.9959	0.9877	1.0042	1.0074	0.9889	1.0264	>0.9999	0.4056
**NO_2**	0.9948	0.9910	0.9986	0.9959	0.9917	1.0000	0.9908	0.9816	1.0000	<0.0001	<0.0001
**L_{den}**	1.0025	0.9993	1.0058	1.0032	0.9996	1.0067	0.9996	0.9917	1.0076	0.0007	<0.0001
**Greenspace** 1000m	1.0007	0.9998	1.0015	1.0008	0.9999	1.0018	1.0000	0.9978	1.0021	0.0566	0.0024

Note: Estimates from Model 4 (i.e. including all explanatory variables). Bonferroni-adjusted (~99.9%) confidence intervals. OR = odds ratio; CI = confidence interval; PM = particulate matter; NO_2 = nitrogen dioxide; L_{den} = day-evening-night noise level. Odds ratios indicate proportional odds ratios for a 1-unit increase in the explanatory variable on level of self-rated health.

Figures S22-S24. Confidence interval plots self-rated health stratified by age

Sociodemographic and psychosocial factors

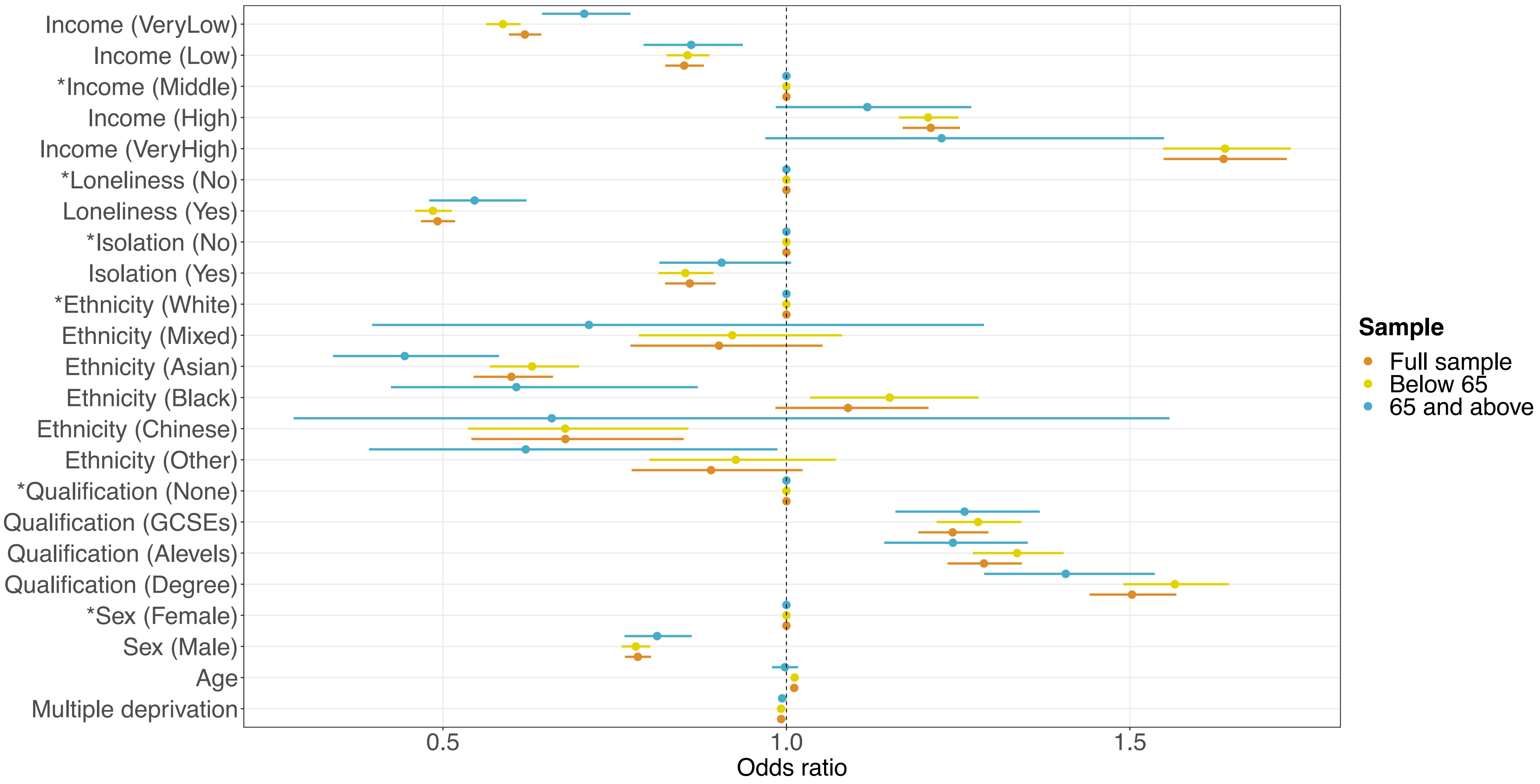

Figure S22. Sociodemographic characteristics and psychosocial factors associated with self-rated health, stratified by age. Confidence interval plot (odds ratio ± Bonferroni-adjusted (~99.9%) confidence intervals) for Model 4 (i.e. including all explanatory variables). GCSEs = general certificate of secondary education. *Indicates reference group for categorical explanatory variables. Annual household income groups: very low (<£18,000), low (£18,000–£30,999), middle (£31,000–£51,999), high (£52,000–£100,000) and very high (>£100,000). 'GCSEs' also includes O levels and certificate of secondary education (CSE). 'A levels' also includes national vocational qualification (NVQ), higher national diploma (HND), higher national certificate (HNC) and 'other professional qualifications'.

Lifestyle factors

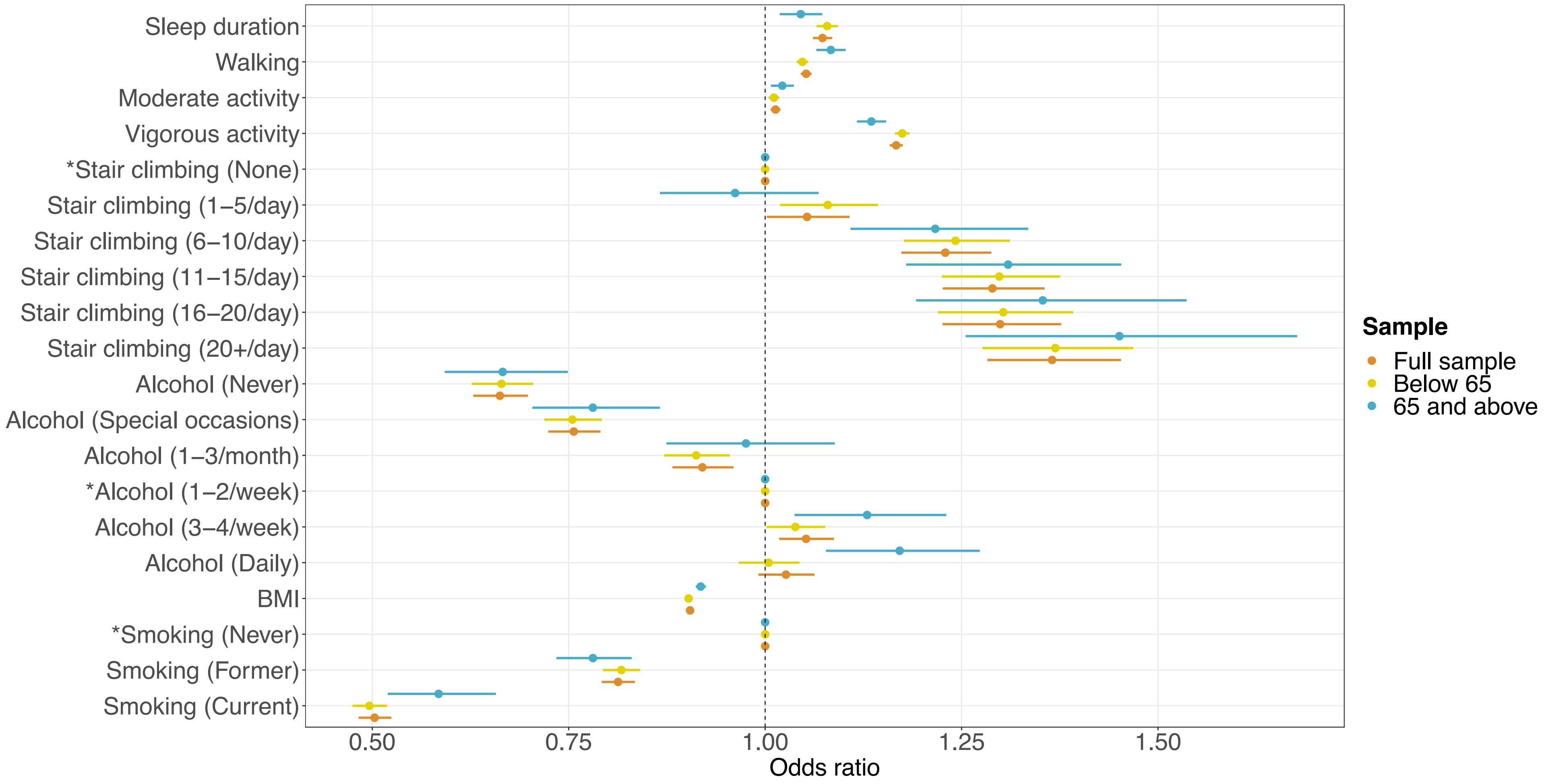

Figure S23. Lifestyle factors associated with self-rated health, stratified by age. Confidence interval plot (odds ratio ± Bonferroni-adjusted (~99.9%) confidence intervals) for Model 4 (i.e. including all explanatory variables). BMI = body mass index. *Indicates reference group for categorical explanatory variables.

Environmental exposures

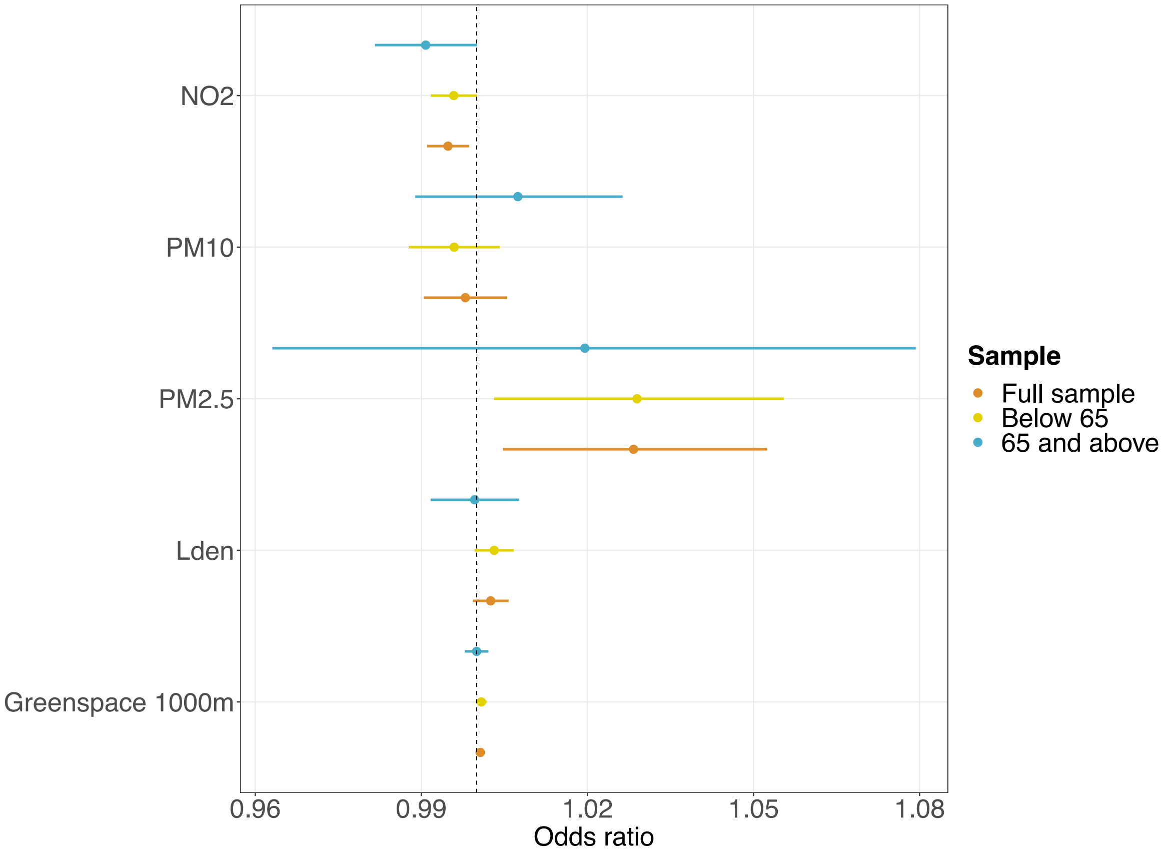

Figure S24. Environmental exposures associated with self-rated health, stratified by age. Confidence interval plot (odds ratio ± Bonferroni-adjusted (~99.9%) confidence intervals) for Model 4 (i.e. including all explanatory variables). PM = particulate matter; NO_2 = nitrogen dioxide; L_{den} = day-evening-night noise level.

Table S51. Baseline characteristics longitudinal samples

Table S51. **Baseline characteristics of longitudinal samples**

	Analytical sample (N = 307 378)	Follow-up t1 (N = 16 058)	Follow-up t2 (N = 32 617)
**Self-rated health**			
Poor	11 066 (3.6%)	469 (2.9%)	677 (2.1%)
Fair	59 169 (19.2%)	2 834 (17.6%)	5 197 (15.9%)
Good	182 699 (59.4%)	9 790 (61.0%)	20 653 (63.3%)
Excellent	54 444 (17.7%)	2 965 (18.5%)	6 090 (18.7%)
Sociodemographic characteristics			
**Age**			
Mean (SD)	56.10 (8.07)	56.92 (7.39)	54.84 (7.46)
Range	38.00-73.00	40.00-73.00	40.00-70.00
**Sex**			
Female	159 574 (51.9%)	7 951 (49.5%)	16 344 (50.1%)
Male	147 804 (48.1%)	8 107 (50.5%)	16 273 (49.9%)
**Ethnicity**			
White	293 565 (95.5%)	15 748 (98.1%)	31 768 (97.4%)
Mixed-race	1 766 (0.6%)	58 (0.4%)	139 (0.4%)
Black	4 257 (1.4%)	63 (0.4%)	191 (0.6%)
Asian	4 755 (1.5%)	84 (0.5%)	298 (0.9%)
Chinese	818 (0.3%)	37 (0.2%)	82 (0.3%)
Other	2 217 (0.7%)	68 (0.4%)	139 (0.4%)
**Highest qualification**			
None	39 828 (13.0%)	1 156 (7.2%)	1 854 (5.7%)
O levels/GCSEs/CSEs	84 448 (27.5%)	3 620 (22.5%)	7 748 (23.8%)
A levels/NVQ/HND/HNC[1]	72 584 (23.6%)	3 819 (23.8%)	7 608 (23.3%)
Degree	110 518 (36.0%)	7 463 (46.5%)	15 407 (47.2%)
**Household income**[2]			
Very low	82 338 (26.8%)	4 679 (29.1%)	9 910 (30.4%)
Low	63 099 (20.5%)	2 562 (16.0%)	3 824 (11.7%)
Medium	77 931 (25.4%)	4 412 (27.5%)	7 308 (22.4%)
High	66 106 (21.5%)	3 595 (22.4%)	9 231 (28.3%)
Very high	17 904 (5.8%)	810 (5.0%)	2 344 (7.2%)
**Multiple deprivation**			
Mean (SD)	16.77 (13.33)	16.51 (12.93)	15.49 (12.16)
Range	0.61-82.00	1.12-81.59	0.61-81.59
Psychosocial factors			
**Loneliness**			
Not lonely	289 901 (94.3%)	15 303 (95.3%)	31 145 (95.5%)
Lonely	17 477 (5.7%)	755 (4.7%)	1 472 (4.5%)
**Social isolation**			
Not isolated	280 931 (91.4%)	14 930 (93.0%)	30 337 (93.0%)
Isolated	26 447 (8.6%)	1 128 (7.0%)	2 280 (7.0%)
Lifestyle factors			
**Smoking status**			
Never	168 475 (54.8%)	9 478 (59.0%)	19 674 (60.3%)
Former	108 638 (35.3%)	5 596 (34.8%)	10 910 (33.4%)
Current	30 265 (9.8%)	984 (6.1%)	2 033 (6.2%)
**Stair climbing frequency**			
None	24 049 (7.8%)	1 042 (6.5%)	1 977 (6.1%)
1-5/day	58 267 (19.0%)	2 670 (16.6%)	5 476 (16.8%)
6-10/day	115 982 (37.7%)	6 165 (38.4%)	12 777 (39.2%)
11-15/day	60 315 (19.6%)	3 421 (21.3%)	6 939 (21.3%)
16-20/day	27 609 (9.0%)	1 600 (10.0%)	3 141 (9.6%)
20+/day	21 156 (6.9%)	1 160 (7.2%)	2 307 (7.1%)
**Alcohol intake frequency**			
Never	78 777 (25.6%)	3 978 (24.8%)	8 327 (25.5%)
Special occasions	20 423 (6.6%)	822 (5.1%)	1 457 (4.5%)
1-3/month	31 526 (10.3%)	1 343 (8.4%)	2 569 (7.9%)
1-2/week	33 798 (11.0%)	1 699 (10.6%)	3 514 (10.8%)
3-4/week	75 251 (24.5%)	4 496 (28.0%)	9 291 (28.5%)
Daily/almost daily	67 603 (22.0%)	3 720 (23.2%)	7 459 (22.9%)
**Sleep duration** (hours/day)			
Mean (SD)	7.16 (1.06)	7.20 (0.98)	7.16 (0.96)
Range	1.00-20.00	1.00-15.00	1.00-18.00
**BMI** (kg/m^2)			
Mean (SD)	27.27 (4.66)	26.83 (4.46)	26.68 (4.27)
Range	12.80-67.30	15.80-56.60	15.20-63.60
**Walking** (days/week)[3]			
Mean (SD)	5.36 (1.95)	5.17 (2.02)	5.21 (2.00)
Range	0.00-7.00	0.00-7.00	0.00-7.00
**Moderate activity** (days/week)[3]			
Mean (SD)	3.59 (2.32)	3.45 (2.28)	3.44 (2.26)
Range	0.00-7.00	0.00-7.00	0.00-7.00
**Vigorous activity** (days/week)[3]			
Mean (SD)	1.88 (1.94)	1.83 (1.85)	1.89 (1.84)
Range	0.00-7.00	0.00-7.00	0.00-7.00

Environmental exposures			
**$PM_{2.5}$**			
Mean (SD)	9.95 (1.04)	9.96 (1.03)	9.92 (1.04)
Range	8.17-21.25	8.17-19.65	8.17-19.65
**PM_{10}**			
Mean (SD)	16.19 (1.88)	15.92 (1.85)	16.00 (1.85)
Range	11.78-30.65	11.78-25.38	11.78-26.33
**NO_2**			
Mean (SD)	26.43 (7.56)	25.99 (6.68)	25.72 (7.15)
Range	12.93-108.49	12.93-89.08	12.93-89.55
**L_{den}**			
Mean (SD)	56.01 (4.24)	56.12 (4.33)	55.98 (4.19)
Range	51.55-89.29	51.57-81.36	51.56-81.67
**Greenspace** 1000m			
Mean (SD)	45.52 (21.77)	48.37 (20.62)	47.95 (21.77)
Range	4.49-99.19	8.90-98.29	4.96-98.98

Note: All variables assessed at baseline except self-rated health in the third and fourth column assessed at t1 and t2, respectively. GCSEs = general certificate of secondary education; CSE = certificate of secondary education; NVQ = national vocational qualification; HND = higher national diploma; HNC = higher national certificate; BMI = body mass index; PM = particulate matter; NO_2 = nitrogen dioxide; L_{den} = day-evening-night noise level. [1]also includes 'other professional qualifications'. [2]Annual household income groups: very low (<£18,000), low (£18,000–£30,999), middle (£31,000–£51,999), high (£52,000–£100,000) and very high (>£100,000). [3]number of days per week engaging in these activities for 10+ minutes continuously.

Tables S52-S55. Regression tables self-rated health t1

Sociodemographic characteristics

Table S52. **Sociodemographic characteristics at baseline associated with self-rated health at t1**

	Model 1			Model 2			Model 3			Model 4		
Term	OR	Bonferroni-corrected CI		OR	Bonferroni-corrected CI		OR	Bonferroni-corrected CI		OR	Bonferroni-corrected CI	
**Household income**[1]												
Very low	0.6337	0.5411	0.7421	0.6173	0.5256	0.7251	0.6989	0.5919	0.8253	0.7396	0.6234	0.8773
Low	0.8404	0.7346	0.9613	0.8254	0.7201	0.9461	0.8649	0.7539	0.9922	0.8789	0.7645	1.0103
Middle	Ref	–	–	Ref	–	–	Ref	–	–	Ref	–	–
High	1.3217	1.1460	1.5244	1.3423	1.1625	1.5501	1.2605	1.0901	1.4577	1.2876	1.1111	1.4924
Very high	1.7091	1.3381	2.1817	1.7355	1.3573	2.2179	1.5644	1.2200	2.0046	1.5147	1.1769	1.9488
**Sex**												
Female	Ref	–	–	Ref	–	–	Ref	–	–	Ref	–	–
Male	0.8676	0.7842	0.9598	–	–	–	0.8471	0.7647	0.9384	0.8864	0.7965	0.9862
**Age**	0.9905	0.9837	0.9974	–	–	–	1.0019	0.9943	1.0095	1.0006	0.9927	1.0085
**Multiple deprivation**	0.9853	0.9814	0.9892	0.9847	0.9808	0.9887	0.9898	0.9857	0.9939	0.9964	0.9920	1.0009
**Ethnicity**												
White	Ref	–	–	Ref	–	–	Ref	–	–	Ref	–	–
Mixed-race	0.5693	0.2410	1.3718	0.5348	0.2258	1.2915	0.5679	0.2393	1.3722	0.7006	0.2942	1.6803
Asian	0.6917	0.3451	1.4062	0.6640	0.3309	1.3514	0.6670	0.3333	1.3501	0.6824	0.3359	1.3994
Black	0.9514	0.4177	2.1879	0.8885	0.3899	2.0446	1.1738	0.5123	2.7163	1.5064	0.6503	3.5222
Chinese	0.8760	0.3190	2.4515	0.8202	0.2984	2.2975	0.8894	0.3215	2.5053	0.7685	0.2734	2.1899
Other	0.6262	0.2924	1.3657	0.6158	0.2876	1.3422	0.5860	0.2735	1.2727	0.6499	0.2965	1.4386
**Highest qualification**												
None	Ref	–	–	Ref	–	–	Ref	–	–	Ref	–	–
O levels/GCSEs/CSEs	1.2827	1.0335	1.5911	1.2291	0.9870	1.5296	1.1175	0.8956	1.3935	1.0969	0.8765	1.3722
A levels/NVQ/HND/HNC[2]	1.3799	1.1130	1.7098	1.3360	1.0748	1.6597	1.1622	0.9321	1.4484	1.1282	0.9018	1.4110
Degree	1.8163	1.4821	2.2247	1.7446	1.4179	2.1455	1.4018	1.1328	1.7340	1.2253	0.9856	1.5230

Note: Bonferroni-adjusted (~99.9%) confidence intervals. OR = odds ratio; CI = confidence interval; GCSEs = general certificate of secondary education; CSE = certificate of secondary education; NVQ = national vocational qualification; HND = higher national diploma; HNC = higher national certificate. For categorical explanatory variables the odds ratios indicate the changes in odds of reporting better self-rated health associated with the explanatory variable group relative to the reference group. Odds ratios for continuous explanatory variables indicate proportional odds ratios for a 1-unit increase in the explanatory variable on level of self-rated health.
[1]Annual household income groups: very low (<£18,000), low (£18,000–£30,999), middle (£31,000–£51,999), high (£52,000–£100,000) and very high (>£100,000). [2]also includes 'other professional qualifications'.
Model 1 – only individual explanatory variables; Model 2 – adjusted for age and sex; Model 3 – age, sex and all sociodemographic characteristics. Model 4 – all explanatory variables. *All models adjusted for number of days between t0 and t1.

Psychosocial factors

Table S53. **Psychosocial factors at baseline associated with self-rated health at t1**

	Model 1			Model 2			Model 3			Model 4		
Term	OR	Bonferroni-corrected CI		OR	Bonferroni-corrected CI		OR	Bonferroni-corrected CI		OR	Bonferroni-corrected CI	
**Loneliness**												
Not lonely	Ref	–	–	Ref	–	–	Ref	–	–	Ref	–	–
Lonely	0.4145	0.3277	0.5249	0.4120	0.3256	0.5218	0.4342	0.3427	0.5508	0.5321	0.4178	0.6782
**Social isolation**												
Not isolated	Ref	–	–	Ref	–	–	Ref	–	–	Ref	–	–
Isolated	0.5918	0.4851	0.7225	0.5928	0.4857	0.7240	0.6339	0.5187	0.7751	0.8688	0.7065	1.0688

Note: Bonferroni-adjusted (~99.9%) confidence intervals. OR = odds ratio; CI = confidence interval. Odds ratios indicate the changes in odds of reporting better self-rated health associated with the explanatory variable group relative to the reference group.
Model 1 – only individual explanatory variables.
Model 2 – adjusted for age and sex.
Model 3 – age, sex and all psychosocial factors.
Model 4 – all explanatory variables.
*All models adjusted for number of days between t0 and t1.

Table S54. **Lifestyle factors at baseline associated with self-rated health at t1**

	Model 1			Model 2			Model 3			Model 4		
Term	OR	Bonferroni-corrected CI		OR	Bonferroni-corrected CI		OR	Bonferroni-corrected CI		OR	Bonferroni-corrected CI	
**Sleep duration** (hours/day)	1.0941	1.0380	1.1534	1.0998	1.0431	1.1595	1.0957	1.0391	1.1553	1.1018	1.0447	1.1621
**Physical activity** (days/week)[1]												
Walking	1.0986	1.0711	1.1268	1.1016	1.0740	1.1300	1.0518	1.0233	1.0812	1.0589	1.0300	1.0886
Moderate activity	1.0855	1.0615	1.1100	1.0893	1.0651	1.1141	0.9987	0.9718	1.0264	1.0083	0.9809	1.0364
Vigorous activity	1.1803	1.1478	1.2138	1.1857	1.1528	1.2195	1.1389	1.1023	1.1768	1.1313	1.0947	1.1691
**Stair climbing frequency**												
None	Ref	–	–	Ref	–	–	Ref	–	–	Ref	–	–
1-5/day	0.9367	0.7417	1.1823	0.9110	0.7204	1.1514	0.9620	0.7593	1.2185	0.9228	0.7272	1.1707
6-10/day	1.3028	1.0521	1.6122	1.2673	1.0224	1.5701	1.1913	0.9592	1.4790	1.1083	0.8904	1.3791
11-15/day	1.5277	1.2186	1.9146	1.4856	1.1840	1.8635	1.2798	1.0175	1.6093	1.1678	0.9259	1.4725
16-20/day	1.5523	1.2038	2.0013	1.5070	1.1676	1.9445	1.2351	0.9544	1.5981	1.1308	0.8714	1.4673
20+/day	1.7904	1.3621	2.3528	1.7285	1.3137	2.2738	1.3282	1.0055	1.7543	1.2175	0.9191	1.6126
**Alcohol intake frequency**												
Never	0.6707	0.5239	0.8592	0.6636	0.5182	0.8506	0.6424	0.5005	0.8247	0.7134	0.5541	0.9188
Special occasions	0.6566	0.5369	0.8034	0.6363	0.5197	0.7793	0.6974	0.5682	0.8562	0.7828	0.6362	0.9634
1-3/month	0.7801	0.6480	0.9392	0.7642	0.6346	0.9204	0.7803	0.6470	0.9411	0.8287	0.6864	1.0006
1-2/week	Ref	–	–	Ref	–	–	Ref	–	–	Ref	–	–
3-4/week	1.1624	1.0113	1.3362	1.1800	1.0265	1.3567	1.1079	0.9619	1.2760	1.0416	0.9034	1.2010
Daily/almost daily	1.0895	0.9415	1.2607	1.1385	0.9828	1.3189	1.1093	0.9548	1.2889	1.0369	0.8910	1.2067
**BMI** (kg/m^2)	0.8924	0.8820	0.9029	0.8931	0.8827	0.9036	0.9063	0.8954	0.9172	0.9091	0.8981	0.9202
**Smoking status**												
Never	Ref	–	–	Ref	–	–	Ref	–	–	Ref	–	–
Former	0.7472	0.6705	0.8327	0.7679	0.6880	0.8570	0.7844	0.7007	0.8779	0.8176	0.7296	0.9161
Current	0.4688	0.3785	0.5811	0.4708	0.3799	0.5839	0.4588	0.3688	0.5711	0.5221	0.4186	0.6516

Note: Bonferroni-adjusted (~99.9%) confidence intervals. OR = odds ratio; CI = confidence interval; BMI = body mass index. For categorical explanatory variables the odds ratios indicate the changes in odds of reporting better self-rated health associated with the explanatory variable group relative to the reference group. Odds ratios for continuous explanatory variables indicate proportional odds ratios for a 1-unit increase in the explanatory variable on level of self-rated health. [1]number of days per week engaging in these activities for 10+ minutes continuously.
Model 1 – only individual explanatory variables; Model 2 – adjusted for age and sex; Model 3 – age, sex and all lifestyle factors; Model 4 – all explanatory variables. *All models adjusted for number of days between t0 and t1.

Table S55. **Environmental exposures at baseline associated with self-rated health at t1**

	Model 1			Model 2			Model 3			Model 4		
Term	OR	Bonferroni-corrected CI		OR	Bonferroni-corrected CI		OR	Bonferroni-corrected CI		OR	Bonferroni-corrected CI	
**$PM_{2.5}$**	0.9111	0.8667	0.9579	0.9046	0.8603	0.9511	0.9980	0.8929	1.1158	1.0122	0.9023	1.136
**PM_{10}**	0.9648	0.9386	0.9918	0.9636	0.9374	0.9906	0.9868	0.9536	1.0213	0.9942	0.9601	1.030
**NO_2**	0.9844	0.9766	0.9923	0.9831	0.9753	0.9910	0.9716	0.9536	0.9899	0.9896	0.9703	1.009
**L_{den}**	0.9928	0.9814	1.0044	0.9924	0.9810	1.0040	1.0078	0.9943	1.0215	1.0025	0.9888	1.016
**Greenspace** 1000m	1.0015	0.9989	1.0040	1.0018	0.9992	1.0044	0.9956	0.9917	0.9995	0.9975	0.9935	1.001

Note: Bonferroni-adjusted (~99.9%) confidence intervals. OR = odds ratio; CI = confidence interval; PM = particulate matter; NO_2 = nitrogen dioxide; L_{den} = day-evening-night noise level. Odds ratios for continuous explanatory variables indicate proportional odds ratios for a 1-unit increase in the explanatory variable on level of self-rated health.
Model 1 – only individual explanatory variables.
Model 2 – adjusted for age and sex.
Model 3 – age, sex and all environmental exposures.
Model 4 – all explanatory variables.
*All models adjusted for number of days between t0 and t1.

Tables S56-S59. Regression tables self-rated health t2

Sociodemographic characteristics

Table S56. **Sociodemographic characteristics at baseline associated with self-rated health at t2**

	Model 1			Model 2			Model 3			Model 4		
Term	OR	Bonferroni-corrected CI		OR	Bonferroni-corrected CI		OR	Bonferroni-corrected CI		OR	Bonferroni-corrected CI	
**Household income**[1]												
Very low	0.6433	0.5680	0.7288	0.6003	0.5291	0.6812	0.6968	0.6120	0.7934	0.7042	0.6166	0.8041
Low	0.8741	0.7905	0.9666	0.8314	0.7510	0.9204	0.8841	0.7981	0.9795	0.8647	0.7795	0.9591
Middle	Ref	–	–	Ref	–	–	Ref	–	–	Ref	–	–
High	1.2214	1.1114	1.3423	1.2625	1.1482	1.3884	1.1683	1.0612	1.2863	1.1531	1.0460	1.2713
Very high	1.7805	1.5338	2.0665	1.8603	1.6013	2.1608	1.6382	1.4068	1.9073	1.5583	1.3344	1.8196
**Sex**												
Female	Ref	–	–	Ref	–	–	Ref	–	–	Ref	–	–
Male	0.8796	0.8184	0.9453	–	–	–	0.8331	0.7742	0.8964	0.9112	0.8441	0.9836
**Age**	1.0027	0.9978	1.0076	–	–	–	1.0106	1.0053	1.0160	1.0121	1.0065	1.0176
**Multiple deprivation**	0.9833	0.9804	0.9862	0.9833	0.9803	0.9862	0.9890	0.9859	0.9921	0.9947	0.9914	0.9981
**Ethnicity**												
White	Ref	–	–	Ref	–	–	Ref	–	–	Ref	–	–
Mixed-race	0.9761	0.5584	1.7081	0.9690	0.5542	1.6963	1.0192	0.5869	1.7728	1.0936	0.6267	1.9098
Asian	0.5379	0.3710	0.7837	0.5578	0.3844	0.8132	0.5255	0.3613	0.7673	0.5071	0.3454	0.7469
Black	0.6076	0.3797	0.9797	0.6139	0.3831	0.9911	0.7166	0.4453	1.1607	0.8968	0.5543	1.4566
Chinese	0.6796	0.3389	1.3796	0.6817	0.3399	1.3840	0.6796	0.3376	1.3832	0.5738	0.2805	1.1857
Other	0.8768	0.5001	1.5421	0.8729	0.4976	1.5361	0.8645	0.4934	1.5187	0.8945	0.5040	1.5911
**Highest qualification**												
None	Ref	–	–	Ref	–	–	Ref	–	–	Ref	–	–
O levels/GCSEs/CSEs	1.1908	1.0069	1.4077	1.2177	1.0275	1.4425	1.0974	0.9246	1.3019	1.0646	0.8946	1.2666
A levels/NVQ/HND/HNC[2]	1.3584	1.1481	1.6066	1.3899	1.1732	1.6460	1.1957	1.0069	1.4194	1.1558	0.9706	1.3762
Degree	1.8206	1.5513	2.1357	1.8744	1.5941	2.2032	1.4749	1.2486	1.7418	1.3026	1.0988	1.5439

Note: Bonferroni-adjusted (~99.9%) confidence intervals. OR = odds ratio; CI = confidence interval; GCSEs = general certificate of secondary education; CSE = certificate of secondary education; NVQ = national vocational qualification; HND = higher national diploma; HNC = higher national certificate. For categorical explanatory variables the odds ratios indicate the changes in odds of reporting better self-rated health associated with the explanatory variable group relative to the reference group. Odds ratios for continuous explanatory variables indicate proportional odds ratios for a 1-unit increase in the explanatory variable on level of self-rated health.
[1]Annual household income groups: very low (<£18,000), low (£18,000–£30,999), middle (£31,000–£51,999), high (£52,000–£100,000) and very high (>£100,000). [2]also includes 'other professional qualifications'.
Model 1 – only individual explanatory variables; Model 2 – adjusted for age and sex; Model 3 – age, sex and all sociodemographic characteristics; Model 4 – all explanatory variables. *All models adjusted for number of days between t0 and t2.

Table S57. **Psychosocial factors at baseline associated with self-rated health at t2**

	Model 1			Model 2			Model 3			Model 4		
Term	OR	Bonferroni-corrected CI		OR	Bonferroni-corrected CI		OR	Bonferroni-corrected CI		OR	Bonferroni-corrected CI	
**Loneliness**												
Not lonely	Ref	–	–	Ref	–	–	Ref	–	–	Ref	–	–
Lonely	0.3934	0.3318	0.4669	0.3950	0.3331	0.4687	0.4144	0.3492	0.4921	0.5103	0.4281	0.6086
**Social isolation**												
Not isolated	Ref	–	–	Ref	–	–	Ref	–	–	Ref	–	–
Isolated	0.6071	0.5271	0.6995	0.6099	0.5295	0.7027	0.6512	0.5650	0.7508	0.8303	0.7172	0.9615

Note: Bonferroni-adjusted (~99.9%) confidence intervals. OR = odds ratio; CI = confidence interval. For categorical explanatory variables the odds ratios indicate the changes in odds of reporting better self-rated health associated with the explanatory variable group relative to the reference group.
Model 1 – only individual explanatory variables.
Model 2 – adjusted for age and sex.
Model 3 – age, sex and all psychosocial factors.
Model 4 – all explanatory variables.
*All models adjusted for number of days between t0 and t2.

Table S58. **Lifestyle factors at baseline associated with self-rated health at t2**

	Model 1			Model 2			Model 3			Model 4		
Term	OR	Bonferroni-corrected CI		OR	Bonferroni-corrected CI		OR	Bonferroni-corrected CI		OR	Bonferroni-corrected CI	
**Sleep duration** (hours/day)	1.1006	1.0589	1.1438	1.0969	1.0553	1.1401	1.0767	1.0358	1.1193	1.0756	1.0345	1.1183
**Physical activity** (days/week)[1]												
Walking	1.0756	1.0561	1.0953	1.0736	1.0542	1.0934	1.0221	1.0021	1.0425	1.0289	1.0087	1.0495
Moderate activity	1.0788	1.0617	1.0963	1.0777	1.0604	1.0952	0.9927	0.9733	1.0125	1.0029	0.9832	1.0230
Vigorous activity	1.1661	1.1431	1.1896	1.1723	1.1491	1.1961	1.1383	1.1119	1.1653	1.1278	1.1015	1.1547
**Stair climbing frequency**												
None	Ref	–	–	Ref	–	–				Ref	–	–
1-5/day	0.9418	0.7937	1.1173	0.9570	0.8059	1.1362	1.0167	0.8549	1.2089	0.9539	0.8009	1.1360
6-10/day	1.1940	1.0202	1.3973	1.2108	1.0336	1.4180	1.1587	0.9879	1.3590	1.0481	0.8920	1.2315
11-15/day	1.3456	1.1398	1.5883	1.3606	1.1517	1.6072	1.2022	1.0160	1.4224	1.0756	0.9072	1.2752
16-20/day	1.5273	1.2671	1.8408	1.5393	1.2762	1.8563	1.3133	1.0867	1.5871	1.1764	0.9715	1.4245
20+/day	1.5307	1.2540	1.8683	1.5464	1.2657	1.8891	1.2657	1.0334	1.5501	1.1442	0.9325	1.4038
**Alcohol intake frequency**												
Never	0.7111	0.5895	0.8583	0.6996	0.5798	0.8446	0.7176	0.5940	0.8671	0.8048	0.6646	0.9748
Special occasions	0.6698	0.5781	0.7762	0.6456	0.5568	0.7487	0.7173	0.6181	0.8326	0.7999	0.6880	0.9300
1-3/month	0.8298	0.7278	0.9461	0.8148	0.7145	0.9293	0.8548	0.7489	0.9756	0.9051	0.7924	1.0338
1-2/week	Ref	–	–	Ref	–	–				Ref	–	–
3-4/week	1.1309	1.0254	1.2472	1.1380	1.0318	1.2552	1.0713	0.9702	1.1831	1.0085	0.9126	1.1145
Daily/almost daily	1.0750	0.9689	1.1927	1.0902	0.9816	1.2107	1.0647	0.9567	1.1848	0.9799	0.8795	1.0917
**BMI** (kg/m^2)	0.8848	0.8771	0.8926	0.8846	0.8769	0.8924	0.8960	0.8879	0.9041	0.8992	0.8911	0.9074
**Smoking status**												
Never	Ref	–	–	Ref	–	–				Ref	–	–
Former	0.7758	0.7176	0.8385	0.7744	0.7158	0.8378	0.7985	0.7367	0.8654	0.8330	0.7680	0.9035
Current	0.4923	0.4228	0.5733	0.5010	0.4302	0.5837	0.5104	0.4369	0.5965	0.5724	0.4893	0.6698

Note: Bonferroni-adjusted (~99.9%) confidence intervals. OR = odds ratio; CI = confidence interval; BMI = body mass index. For categorical explanatory variables the odds ratios indicate the changes in odds of reporting better self-rated health associated with the explanatory variable group relative to the reference group. Odds ratios for continuous explanatory variables indicate proportional odds ratios for a 1-unit increase in the explanatory variable on level of self-rated health. [1]number of days per week engaging in these activities for 10+ minutes continuously.
Model 1 – only individual explanatory variables; Model 2 – adjusted for age and sex; Model 3 – age, sex and all lifestyle factors; Model 4 – all explanatory variables. *All models adjusted for number of days between t0 and t2.

Table S59. **Environmental exposures at baseline associated with self-rated health at t2**

	Model 1			Model 2			Model 3			Model 4		
Term	OR	Bonferroni-corrected CI		OR	Bonferroni-corrected CI		OR	Bonferroni-corrected CI		OR	Bonferroni-corrected CI	
**$PM_{2.5}$**	0.9205	0.8889	0.9531	0.9213	0.8896	0.9540	0.9251	0.8595	0.9957	0.9855	0.9140	1.063
**PM_{10}**	0.9821	0.9631	1.0014	0.9825	0.9635	1.0018	1.0105	0.9872	1.0343	1.0174	0.9936	1.042
**NO_2**	0.9890	0.9840	0.9940	0.9891	0.9841	0.9942	0.9955	0.9834	1.0079	0.9998	0.9872	1.013
**L_{den}**	0.9920	0.9835	1.0006	0.9921	0.9836	1.0007	0.9978	0.9881	1.0076	0.9976	0.9878	1.008
**Greenspace** 1000m	1.0021	1.0004	1.0037	1.0021	1.0004	1.0037	0.9989	0.9963	1.0016	1.0004	0.9978	1.003

Note: Bonferroni-adjusted (~99.9%) confidence intervals. OR = odds ratio; CI = confidence interval; PM = particulate matter; NO_2 = nitrogen dioxide; L_{den} = day-evening-night noise level. Odds ratios for continuous explanatory variables indicate proportional odds ratios for a 1-unit increase in the explanatory variable on level of self-rated health.
Model 1 – only individual explanatory variables.
Model 2 – adjusted for age and sex.
Model 3 – age, sex and all environmental exposures.
Model 4 – all explanatory variables.
*All models adjusted for number of days between t0 and t2.

Table S60. Descriptive statistics additional analyses

Descriptive statistics

Table S60. **Descriptive statistics additional analyses**

Variable	Statistics
**Body fat percentage**	Mean (SD)
	30.85 (8.46)
	Range
	5-65.2
**Alcohol intake frequency**	*n* (%)
Lifetime abstainers	10 599 (3.45)
Current abstainers	9 792 (3.19)
Special occasions	31 526 (10.26)
1-3/month	33 798 (11.00)
1-2/week	78 777 (25.63)
3-4/week	75 251 (24.48)
Daily/almost daily	67 603 (22.00)
**Current tobacco smoking**	*n* (%)
"Yes, on most or all days"	21 778 (7.09)
"Only occasionally"	8 487 (2.76)
"No"	277 107 (90.15)
**Metabolic Equivalent Task minutes/week**	Median (IQR)
Walking	693 (1089)
Moderate activity	480 (1080)
Vigorous activity	240 (960)
**$PM_{2.5}$**	*n* (%)
≤10 $\mu g/m^3$	167 631 (54.54)
>10 $\mu g/m^3$	139 747 (45.46)
**PM_{10}**	*n* (%)
≤20 $\mu g/m^3$	290 684 (94.57)
>20 $\mu g/m^3$	16 694 (5.43)
**NO_2**	*n* (%)
≤40 $\mu g/m^3$	294 402 (95.78)
>40 $\mu g/m^3$	12 976 (4.22)
**L_{den}**	*n* (%)
≤53 decibels	61 017 (19.85)
>53 decibels	246 361 (80.15)
**Greenspace 300m**	Mean (SD)
	35.65 (23.46)
	Range
	0.227-99.180

Note: SD = standard deviation; IQR = interquartile range; PM = particulate matter; NO_2 = nitrogen dioxide; L_{den} = day-evening-night noise level.

Figure S25. Generalised additive models

To identify potential non-linear associations between continuous explanatory variables and health status, we fitted binomial generalised additive models (presented below).

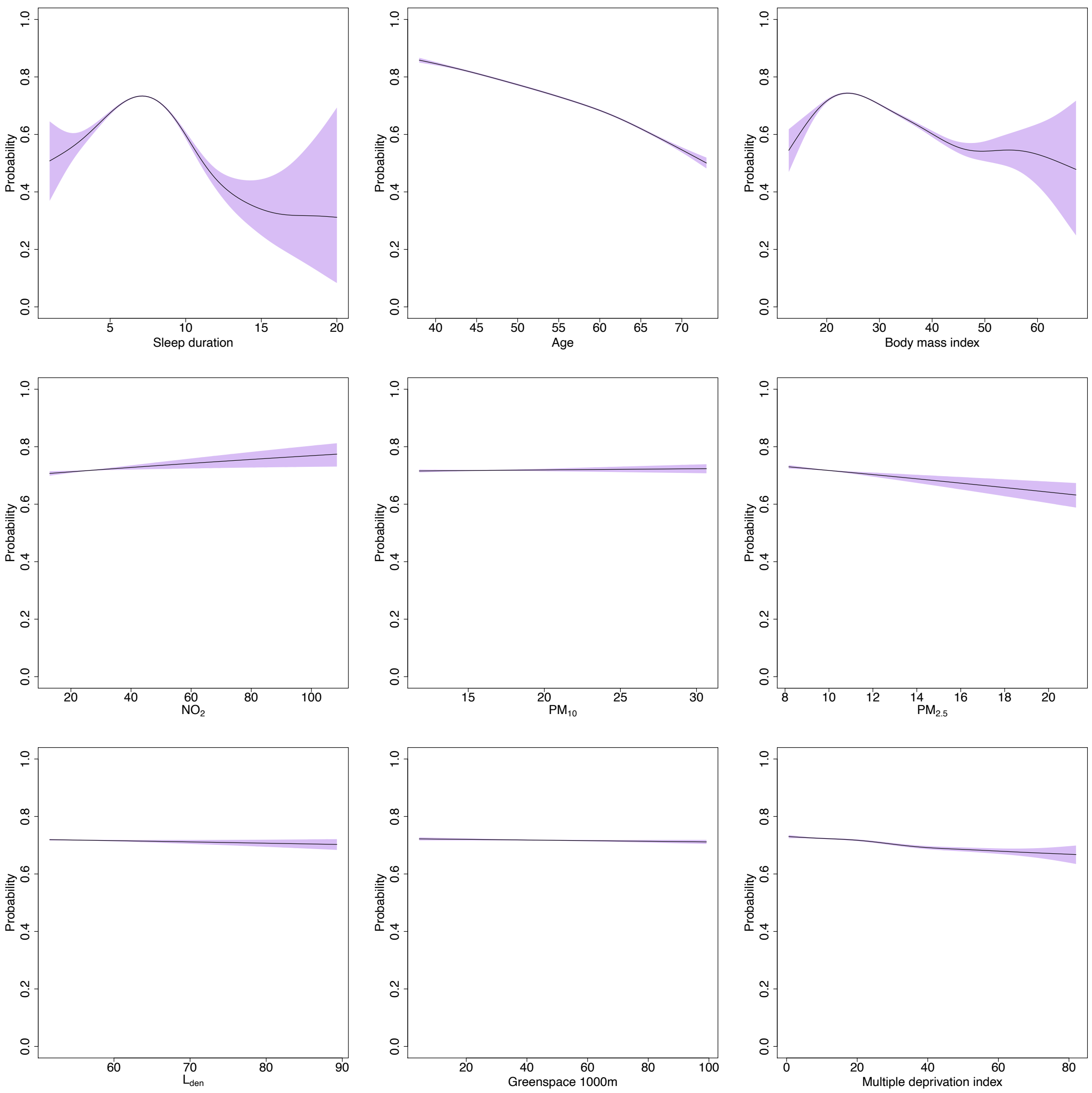

Figure S25. Probabilities of favourable health status (healthy: ≥ 0.50; unhealthy <0.50) by continuous explanatory variables. Results are from a binomial generalised additive model that included all explanatory variables of the full multivariable logistic regression model (Model 4). The shaded areas correspond to approximate 95% confidence intervals (± 2 × standard error). L_{den} = day-evening-night noise level; PM = particulate matter; NO_2 = nitrogen dioxide.
